# Authentication of Maltese Pork Meat Unveiling Insights Through ATR-FTIR and Chemometric Analysis

**DOI:** 10.3390/foods14203510

**Published:** 2025-10-15

**Authors:** Frederick Lia, Mark Caffari, Malcom Borg, Karen Attard

**Affiliations:** 1 BKEA BioKimika Extrakta Analytika Ltd., RBT 3140 Rabat, Malta; 2 Institute of Applied Sciences (IAS), The Malta College of Arts, Science and Technology (MCAST), PLA 9032 Paola, Malta; mark.caffari.f32093@mcast.edu.mt; 3 Centre of Agriculture, Aquatics and Animal Sciences, Institute of Applied Sciences (IAS), The Malta College of Arts, Science and Technology (MCAST), QRM 9075 Qormi, Malta; malcolm.b.borg@mcast.edu.mt

**Keywords:** FTIR spectroscopy, pork authentication, chemometrics, PLSR, SVM, artificial neural networks

## Abstract

Ensuring the authenticity of meat products is a critical issue for consumer protection, regulatory compliance, and the integrity of local food systems. In this study, attenuated total reflectance Fourier-transform infrared (ATR-FTIR) spectroscopy combined with chemometric and machine learning models was applied to differentiate Maltese from non-Maltese pork. Spectral datasets were subjected to a range of preprocessing techniques, including Savitzky–Golay first and second derivatives, detrending, orthogonal signal correction (OSC), and standard normal variate (SNV). Linear methods such as principal component analysis–linear discriminant analysis (PCA-LDA), the soft independent modeling of class analogy (SIMCA), and partial least squares regression (PLSR) were compared against nonlinear approaches, namely support vector machine regression (SVMR) and artificial neural networks (ANNs). The results revealed that derivative preprocessing consistently enhanced spectral resolution and model robustness, with the fingerprint region (1800–600 cm^−1^) yielding the highest discriminative power. While PCA-LDA, SIMCA, and PLSR achieved high accuracy, SVMR and ANN models provided a superior predictive performance, with accuracies exceeding 0.99 and lower misclassification rates under external validation. These findings highlight the potential of FTIR spectroscopy combined with nonlinear chemometrics as a rapid, non-destructive, and cost-effective strategy for meat authentication, supporting both consumer safety and sustainable food supply chains.

## 1. Introduction

Global concerns regarding food fraud have intensified, particularly in the meat sector, where issues such as species substitution, origin misrepresentation, and mislabeling of halal or organic claims are increasingly reported [1,2]. Economically motivated adulteration poses risks that extend beyond consumer deception, encompassing religious sensitivities, nutritional misrepresentation, and even potential toxicological hazards. For example, pork and its derivatives are considered haram in Muslim communities, making reliable detection crucial for halal certification [3]. At the same time, adulteration undermines local economies when high-value meats such as beef or indigenous pork are replaced with cheaper alternatives.

Traditional methods for meat authentication rely on DNA-based assays, such as a polymerase chain reaction (PCR), and protein-based techniques like an enzyme-linked immunosorbent assay (ELISA). While sensitive and specific, these techniques are often hindered by high operational costs, a laborious sample preparation, and reduced applicability to processed or thermally treated meat, where DNA and proteins may degrade [4]. These limitations have motivated the development of vibrational spectroscopic approaches, particularly Fourier-transform infrared (FTIR) spectroscopy, as rapid, non-destructive, and cost-efficient alternatives [5]. FTIR spectroscopy works by measuring the absorption of infrared (IR) radiation by molecular bonds, producing characteristic spectral fingerprints. Its operational range spans the near-infrared (14,000–4000 cm^−1^), mid-infrared (4000–400 cm^−1^), and far-infrared (400–50 cm^−1^) regions, of which the mid-infrared (MIR) region is most relevant for food authentication, since it contains the fundamental vibrational frequencies of lipids, proteins, and nucleic acids [6,7]. Modern FTIR instruments utilize interferometers, typically based on the Michelson design, which allow the multiplexing of wavelengths and improve resolution and signal-to-noise ratios compared to dispersive IR systems [8]. Importantly, the application of attenuated total reflectance (ATR)-FTIR has simplified sample handling by allowing the direct measurement of intact or heterogeneous samples without extensive preprocessing. Crystals such as diamond, ZnSe, or Ge facilitate the penetration of IR radiation into the sample surface, enabling the analysis of soft tissues, lipids, and protein-rich matrices [7].

In meat analysis, ATR-FTIR spectra commonly exhibit diagnostic peaks: 3000–2800 cm^−1^ (lipid CH stretching), ~1745 cm^−1^ (triglyceride carbonyls), 1650–1540 cm^−1^ (protein Amide I and II bands), and 1200–1000 cm^−1^ (nucleic acids and phospholipids). The application of chemometrics has significantly advanced the interpretability of FTIR spectra. Chemometrics refers to the integration of mathematical and statistical tools into chemical analysis to extract meaningful information from complex datasets [8]. Techniques such as principal component analysis (PCA) and partial least squares (PLS) regression have been used to classify species, identify adulterants, and even quantify adulteration levels in meat products. For example, studies demonstrated that ATR-FTIR combined with PLS regression achieved correlation coefficients (R^2^) greater than 0.99 in quantifying lard in butter and differentiating beef sausages adulterated with pork fat [9,10]. Other approaches such as PLS-DA, SIMCA, and support vector machines (SVM) have further enhanced the classification accuracy in multi-class meat authentication problems, sometimes reaching accuracies above 98% [11]. Internationally, the use of ATR-FTIR combined with chemometrics has extended beyond meat to fats, oils, dairy, and functional foods. A comprehensive review of over two decades of studies revealed that edible fats and oils were among the most adulterated food categories, with FTIR emerging as one of the most reliable fingerprinting tools when coupled with multivariate analysis [12]. In meat applications, FTIR has been applied for detecting pork adulteration in beef meatballs, lamb sausages, and mixed minced meats, with detection limits often as low as a 1–2% substitution [13,14]. Importantly, portable ATR-FTIR and diffuse reflectance (DR)-FTIR devices have recently been evaluated for on-site authenticity testing, achieving classification accuracies of up to 100% when coupled with SVM models [15].

In Malta, the case of pork authentication carries unique socio-economic and cultural significance. Historically, pork was a dietary staple, and its supply was severely disrupted during outbreaks of African Swine Fever. Although the sector has since recovered, a new challenge has emerged: competition from imported pork products that are often cheaper but of a lower quality. Current slaughter rates in Malta stand at approximately 1600 pigs per week, a sharp decline from 2400 in recent years, despite consumption levels remaining constant [16]. The shortfall has been filled by imports, raising concerns about both the quality and authenticity. For a small island nation, where pork holds cultural value and represents a critical component of local agriculture, the risks of adulteration—whether through species substitution, origin misrepresentation, or false labelling, have significant economic and consumer trust implications. Finally, although the European Pharmacopoeia has begun incorporating chemometric methods into analytical chapters, their routine adoption in European food industries remains limited [17]. This highlights a gap between methodological innovation and industrial practice. Addressing this gap in the Maltese context through the integration of ATR-FTIR with chemometrics can provide a rapid, non-destructive, and cost-effective solution for pork authentication. The present study, therefore, seeks to pioneer the application of these methods to Maltese pork, ensuring authenticity, strengthening regulatory oversight, and reinforcing consumer confidence in local meat production.

## 2. Materials and Methods

### 2.1. Pork Samples and Preparation

A total of 116 Maltese pork samples consisting of both loin and belly were directly sampled from KIM (Koperattiva Industijali tal-Majjal, Marsa, Malta). Samples were transported under chilled conditions (4 °C) to the laboratory to prevent degradation prior to analysis. Samples were then stored in a freezer at −15 °C before analysis. With respect to foreign pork samples, a total of 53 samples consisting of the loin and belly were sampled and stored at −15 °C before analysis. Before laboratory analysis, both local and foreign pork samples were freeze-dried (BioBase, BK-FD10PT, Jinan, China) for 3 days at −68 °C. After freeze-drying, visible skin, fat, and connective tissue were excised that could interfere in the analysis, and then about 100 g of meat was homogenized and ground in a ratio of 1:1 with dry ice as it minimizes unwanted heat generation due to friction.

### 2.2. ATR-FTIR Measurement

ATR-FTIR measurements were performed using an IRAffinity-1 Shimadzu spectrometer equipped with an attenuated total reflectance (ATR) accessory (Shimadzu, Kyoto, Japan). The instrument was switched on and allowed to stabilize for 30 min prior to analysis. A background spectrum was first acquired (45 scans), followed by measurement of the validation disk (45 scans) to confirm instrument stability and performance. Before each analysis, the ATR crystal surface was thoroughly cleaned with isopropyl alcohol (Biochem Chemopharma, Cosne-Cours-sur-Loire, France) and dried to prevent cross-contamination. Samples were then placed in firm contact with the ATR crystal to ensure optimal penetration of IR radiation. For each sample, spectra were recorded over the 400–5000 cm^−1^ wavenumber range at a resolution of 2 cm^−1^, with 45 co-added scans to improve the signal-to-noise ratio. To account for sample heterogeneity and improve reproducibility, three replicate spectra were collected per sample, with the sample being removed and repositioned on the crystal between replicates. After each measurement, the ATR surface was re-cleaned with isopropyl alcohol, dried before proceeding to the next sample, and a background scan was completed between different samples. To minimize spectral distortion, wavenumber regions associated with atmospheric CO_2_ (2390–2250 cm^−1^) and water vapor (3400–3200 cm^−1^) were excluded from further analysis. Two spectral datasets were prepared for chemometric analysis: Fingerprint region (1800–600 cm^−1^)—selected for its high specificity to functional group vibrations of proteins, lipids, and nucleic acids. Full mid-infrared (MIR) region (4000–500 cm^−1^) included broad spectral information while excluding CO_2_ and possible water interference zones.

### 2.3. Data Treatment

Raw FTIR spectra are inherently complex, containing overlapping peaks, baseline drifts, and scattering effects that can mask subtle chemical differences between meat samples. In order to optimize the discriminatory power of the spectroscopic dataset, a comprehensive suite of eleven spectral pre-processing transformations was systematically applied prior to chemometric modeling. These transformations were implemented in Unscrambler X (Camo Analytics, Mölndal, Sweden), following approaches widely reported in FTIR–chemometric applications for meat and edible fats [18,19]. The applied pre-processing methods included the following: Savitzky–Golay first derivative (SG 1st der.) enhances spectral resolution and minimizes baseline offsets by calculating the first derivative of absorbance values with a polynomial fitting algorithm. Savitzky–Golay second derivative (SG 2nd der.) emphasizes subtle differences in overlapping bands and improves peak resolution, particularly within the protein Amide I and II regions [11]. Dersolve (derivative with smoothing) combines differentiation with noise filtering, balancing detail enhancement with signal stability. Detrend correction removes linear baseline shifts and compensates for scattering effects caused by surface irregularities. Median filter smoothing (5 point) reduces high-frequency noise by replacing each spectral point with the median of its neighbors. Multiplicative Scatter Correction (MSC) corrects multiplicative and additive light scattering variations due to heterogeneous particle sizes and pathlength differences. Orthogonal Signal Correction (OSC) removes spectral variance unrelated to the dependent variable (class membership), improving model robustness [12]. Quantile normalization was also carried out to standardize intensity distributions across spectra, improving comparability. Raw spectra (no treatment) were included as a baseline reference and ATR correction was used to evaluate the added value of pre-processing. Standard Normal Variate (SNV) corrected for scatter and pathlength differences by scaling each spectrum individually. SNV + Detrend combined both SNV scaling and baseline correction to improve reproducibility.

Each pre-processed dataset was structured into a data matrix

(1) X=n×p

where n corresponds to the number of samples (Maltese and foreign pork replicates) after averaging signal from the three independent replicates and p represents the number of spectral variables (wavenumber points). Supervised and unsupervised chemometric methods were carried in Python 3.11 (Python Software Foundation, Wilmington, DE, USA) using the scikit-learn machine learning library, along with NumPy, pandas, and Matplotlib (version 3.7.2) for data processing and visualization.

### 2.4. Principal Component Analysis

Principal Component Analysis (PCA) is a dimensionality reduction technique which is used to transform high-dimensional data into a lower-dimensional space while preserving the variance in the dataset. PCA is useful as it deals with large datasets with thousands of variables in common. PCA works by finding new axes that maximize variance in the data, involving computing the eigenvalues and eigenvectors of the covariance matrix [20]. The mathematical equation of PCA is shown in equation

(2) $$X=TP^{T}+E$$

in which X represents the original data matrix with n observations and p variables, T represents the score matrix in terms of principal components (PCs), P represents the loading matrix containing the eigenvectors that define how the original variables contribute to each principal component, and E represents the residual matrix capturing unexplained variance or noise after projection [20]. In this research, PCA was used to explain the variance within the ATR-FTIR dataset and to visualize clustering trends in relation to pork origin. The extracted PCA scores provided a summary of the sample grouping based on origin, while the PCA loadings provided an insight into the variability of spectral features contributing to differences within the pork profile. Outlier Detection was carried out using two statistical tests: Hotelling’s T^2^ statistic, which measures the leverage of sample i in the score space:

(3) $${T^{2}}_{i}={t_{i}}^{T}\;{S_{t}}^{-1}\;t_{i}$$

where t_i_ is the score vector of sample i and S_t_ is the covariance matrix of the scores, and Q-residuals (Squared Prediction Error), which quantify the variance not captured by the PCA model:

(4) $$Q_{i}={\left\|x_{i}-{\hat{x}}_{i}\right\|}^{2}$$

where x_i_ is the original spectrum and ${\hat{x}}_{i}$ = t_i_ P^T^ is the PCA reconstruction. Samples exceeding the empirical threshold (mean + three standard deviations of the distribution) for either statistic were flagged as outliers and excluded from subsequent classification steps.

### 2.5. The Soft Independent Modeling of Class Analogy (SIMCA)

The Soft Independent Modeling of Class Analogy (SIMCA) algorithm was utilized as a supervised classification method for the spectral datasets. In SIMCA, distinct PCA models are created independently for each predefined class, allowing for the modeling of within-class variance while preserving class-specific structure. Unknown samples are then projected into each class model and their class membership is assessed by calculating the residual distances between the original spectrum and its PCA reconstruction. The validation of the SIMCA models, along with all other supervised models, was performed using three approaches. Training accuracy is determined by the classification of samples within the calibration set. Leave-One-Out (LOO) cross-validation involves excluding each sample one at a time and reclassifying it using models developed from the remaining data. Excluded-row validation, also known as Venetian blind cross validation, systematically omits every 3rd sample from training and classifies it independently. Each unknown spectrum was classified into the class with the lowest residual distance:

(5) $${\hat{y}}_{i}=arg\;min_{k}\;d_{k,i}$$

where d_k,i_ is the residual distance of sample i to class model k. For two-class comparisons, Coomans plots were constructed to visualize sample positions relative to both class models, providing a graphical overview of membership, ambiguous cases, and potential outliers. The SIMCA performance was assessed using different parameters, namely accuracy, defined as the proportion of correctly classified samples relative to the total number of samples:

(6) $$Accuracy=\frac{TP+TN}{TP+TN+FP+FN}$$

where TP = true positives, TN = true negatives, FP = false positives, and FN = false negatives.

Specificity ability of the model to correctly identify negative samples (i.e., correctly rejecting samples from the other class):

(7) $$Specificity=\frac{TN}{TN+FP}$$

Selectivity, known as sensitivity, is defined as the ability of the model to correctly identify positive samples (i.e., correctly accepting samples belonging to the target class):

(8) $$Selectivity=\frac{TP}{TP+FN}$$

### 2.6. Multivariate Classification Using PCA-LDA and PLS-LDA

To investigate the discriminatory power of the spectral data and assess sample classification based on origin, two hybrid chemometric workflows were employed: Principal Component Analysis coupled with Linear Discriminant Analysis (PCA-LDA) and Partial Least Squares Regression coupled with Linear Discriminant Analysis (PLS-LDA). Both approaches combined dimensionality reduction with supervised classification, optimizing interpretability while minimizing model overfitting. All absorbance values were standardized using z-score normalization (mean-centered and scaled to unit variance) via Standard Scaler from scikit-learn, ensuring comparability across wavenumber intensities.

In the PCA-LDA, dimensionality reduction was first achieved by PCA. PCA was performed on the standardized spectral matrix, retaining a maximum of 10 principal components (PCs) or fewer, depending on dataset constraints. The selected PCs, which captured the majority of spectral variance, were then used as input features in Linear Discriminant Analysis (LDA). LDA is a supervised classification algorithm that seeks to maximize between-class variance while minimizing within-class variance in the transformed space. LDA was implemented using the Linear Discriminant Analysis class from scikit-learn and applied to the PCA scores. The resulting canonical scores were plotted to visualize class separation and classification performance was evaluated.

For the PLS-LDA approach, Partial Least Squares analysis (PLS) was first used to reduce data dimensionality by projecting the spectral matrix onto a new set of orthogonal latent variables (LVs) that are maximally correlated with the class labels (encoded as binary integers: 0 = non-Maltese, 1 = Maltese). A maximum of 10 LVs or fewer were extracted using the PLSRegression class from scikit-learn. The resulting PLS scores (X-scores) served as input features for LDA, implemented in the same manner as the PCA-LDA model. This approach leveraged both the variance in the spectral dataset and its covariance with class membership, potentially offering greater classification power when relevant discriminatory information is subtly embedded in the data structure. Confusion matrices were generated for training predictions and canonical score plots (LD1 vs. LD2) were produced to visualize class separation. Both loading plots and latent variable scores were also exported to aid interpretation of discriminant features. Model outputs and performance metrics were saved for both whole-spectrum and fingerprint-only preprocessing strategies for comparison purposes. The performance of the PCA-LDA and PLS-LDA classification models was assessed using three complementary validation approaches.

#### 2.6.1. Training Accuracy (Apparent Accuracy)

This metric quantifies the proportion of correctly classified samples within the calibration dataset used to train the model. While informative, it may overestimate performance due to overfitting, particularly in high-dimensional datasets with limited samples.

(9) $$AccuracyTrain=\frac{Total\;training\;samples}{Number\;of\;correct\;predictions}\times 100$$

#### 2.6.2. Leave-One-Out Cross-Validation (LOO-CV)

LOO-CV is a robust internal validation method where each sample is iteratively excluded from model training and used for testing. This approach reduces bias and provides more realistic estimate of the model’s predictive ability on unseen data. It is particularly suitable when the dataset is small, as it maximizes training size in each fold.

$$AccuracyLOO=\sum_{i=1}^{n}\frac{\delta \left(y_{i},y^{LOO},i\right)}{n}\times 100$$

(10) $$where\;\delta (a,b)=\left\{\begin{array}{c} 1\;if\;a=b\;\\ 0\;if\;a\;\neq b \end{array}\right.$$

Here, y_i_ is the true class label of the ith sample and y^LOO^i is the predicted class label obtained when the ith sample was excluded from model training.

#### 2.6.3. Excluded Sample Accuracy (Structured Venetian Blind Validation)

In addition to leave-one-out (LOO) cross-validation, a Venetian blinds approach was employed for excluded-sample validation, as this strategy leaves out systematic blocks of spectra rather than single observations, thereby providing a more realistic estimate of prediction error and reducing the tendency of LOO to overestimate error in small samples. In this study, every third sample in the dataset was systematically excluded prior to model training and used solely for model evaluation. This form of stratified sampling ensures that each excluded observation is not adjacent or strongly correlated to those used for training, thereby mimicking an external validation set and avoiding overly optimistic estimates caused by temporal or batch autocorrelation. Specifically, 33% of the samples (every 3rd entry) were withheld and not used during model training. The remaining 67% formed the training set and were used to build the PCA-LDA and PLS-LDA models. Predictions were then generated for the excluded subset and classification accuracy was computed based on the proportion of correctly predicted labels:

(11) $$AccuracyExcluded=\frac{Correct\;predictions\;on\;Venetian\;blind\;excluded\;samples}{Total\;excluded\;samples}\times 100$$

### 2.7. Partial Least Squares Regression (PLSR)

Partial Least Squares Regression (PLSR) was performed using the PLSRegression class from the scikit-learn sklearn.cross_decomposition module. Although the response variable in this study is non-continuous, PLSR was applied to evaluate the variability in classification performance across different spectral transformations and regions by calculating the root mean square error (RMSE). The maximum number of latent variables (LVs) was defined as the minimum between n − 1 (where n is the number of samples) and the number of spectral variables. The optimal number of LVs was selected by minimizing the RMSE obtained from Leave-One-Out (LOO) cross-validation. RMSE values were computed for both the training set and the LOO validation set to assess model performance and reduce the risk of overfitting. In this framework, the binary class response was modeled as a continuous variable rather than a discrete categorical outcome. Class labels were encoded as dummy variables, assigning a value of 1 to Maltese samples and 0 to foreign samples. Predicted values generated by the PLSR model were interpreted probabilistically: samples with predicted values >0.5 were classified as foreign, while those ≤0.5 were classified as Maltese.

Regression coefficients for each wavenumber were extracted from the PLSR model using the optimal number of LVs. Additionally, Variable Importance in Projection (VIP) scores were calculated to assess the relative contribution of each spectral variable to the model. VIP scores were computed following the approach of Wold et al. (2001) [21] using the formula:

(12) $${VIP}_{j}=\sqrt{p\cdot \frac{\sum_{a=1}^{A}\left(w_{j,a}^{2}\cdot s_{a}\right)}{\sum_{a=1}^{A}s_{a}}}$$

where p is the number of variables, w_j,a_ is the weight of variable j on LV a, S_a_ is the amount of variance in y explained by LV a, and A is the number of LVs retained.

PLSR score plots were used to visualize class separation in latent variable space, with samples color-coded by origin (red = foreign, black = Maltese). Regression coefficients and VIP scores were plotted against the original wavenumber axis for interpretability. Model performance was evaluated using the Root Mean Squared Error (RMSE)

(13) $$RMSE=\sqrt{\frac{1}{n}\sum_{i=1}^{n}{\left(y_{i}-\hat{y_{i}}\right)}^{2}}$$

where y_i_ is the reference class label (0 for foreign, 1 for Maltese), $\hat{y_{i}}$ is the corresponding predicted value (continuous output from the PLSR model), and n is the total number of samples evaluated. RMSE was computed for the training set, leave-one-out cross-validation (LOOCV), and excluded rows validation (ERV) to evaluate the accuracy and robustness of the model under different validation strategies.

### 2.8. Support Vector Machine Regression (SVMR) Modeling

Support Vector Machine Regression (SVMR) was implemented using a radial basis function (RBF) kernel via the scikit-learn library. Similar to PLSR, the response was modeled as a continuous variable rather than a discrete categorical outcome. Model hyperparameters were optimized through an exhaustive grid search combined with five-fold cross-validation, using the coefficient of determination (R^2^) as the selection criterion. The hyperparameter space explored included C (regularization parameter): {0.1, 1, 10, 100}; ε (insensitive loss): {0.01, 0.1, 0.5, 1.0}; and γ (kernel coefficient): {‘scale’, ‘auto’}. Model performance was evaluated using the RMSE and coefficient of determination (R^2^) for the training set using leave-one-out cross-validation (LOOCV) and excluded rows validation. To interpret the relative contribution of spectral variables to the SVMR model, permutation importance analysis was performed using 10 randomized repetitions. The top 30 most informative wavenumbers were ranked based on their mean importance scores and visualized for biochemical interpretation.

### 2.9. Artificial Neural Network (ANN) Modeling

A supervised feed-forward Artificial Neural Network (ANN) was employed to classify the geographical origin of the FTIR spectra. The ANN was implemented as a multilayer perceptron (MLP) with rectified linear unit (ReLU) activation functions and optimized using the Adam algorithm hidden layer configurations including single-layer networks with 50 and 100 nodes, two-layer networks (50–20, 100–50), and a three-layer network (50–30–10) combined with maximum iteration limits of 1000, 2000, and 3000. Early stopping based on validation loss was applied in all models to prevent overfitting and reduce computational cost. Classification performance was assessed using accuracy, precision, recall, specificity, F1-score, misclassification rate, cross-entropy loss, and the area under the receiver operating characteristic curve (AUC).

## 3. Results

### 3.1. Spectral Assignments, Peak Identification, and Difference Between Classes

**Figure 1:**
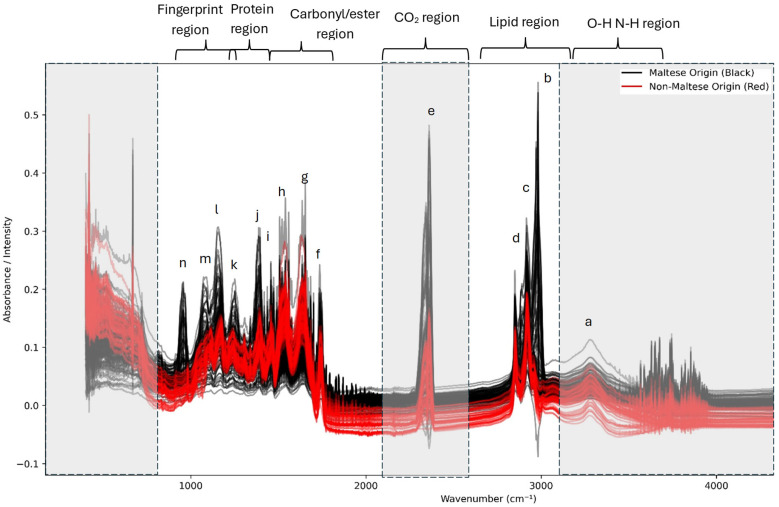
Overlay of ATR-FTIR spectra of Maltese (black) and non-Maltese (red) pork. Characteristic peaks (a–n) are assigned to lipid, protein, ester, and carbohydrate vibrational modes. Major differences are observed in the lipid region (2925, 2853, and 1745 cm^−1^), which is more intense, and in protein bands (1655, 1540, and 1315–1230 cm^−1^), which are stronger in Maltese samples, reflecting higher relative protein content. Gray areas show the excluded regions from model building.

A representative spectrum obtained from the two categories of meat (Maltese versus foreign pork) is shown in Figure 1 and Table 1. The spectra obtained under different transformations can be found in the Supplementary Material Figure S1.

The grey-out regions present in Figure 1 represent the regions which were excluded from the analysis, which included the O-H and N-H region, CO_2_ region, and the last part of the fingerprint region. Overall, the spectra obtained exhibit notable similarities within the overall MIR region (4000–650 cm^−1^); however, several distinct differences can be identified: The O-H and N-H region (a) due to Amide A (the N–H stretching of proteins, with a contribution from the O–H stretching of polysaccharides). The lipid region (3000–2800 cm^−1^) (b,c,d)—prominent CH_2_ asymmetric (~2925 cm^−1^) and symmetric (~2854 cm^−1^) stretching, in addition to CH_3_ asymmetric (~2956–2960 cm^−1^) bands. Variations in the intensity are evident between the classes, reflecting differences in the intramuscular fat composition and saturation levels. The carbonyl/ester region (1745–1740 cm^−1^) (f)—a clear ester C=O stretching band, primarily derived from triglycerides and phospholipids. Intensity variations indicate class-specific differences in the lipid ester content. The protein (Amide I and II) region (1700–1500 cm^−1^) (g,h)—Amide I (~1655 cm^−1^; the C=O stretching of peptide bonds) and Amide II (~1540 cm^−1^; N–H bending and C–N stretching). Both bands are present across all classes, but differ in their relative intensity, suggesting variations in the protein secondary structure profile (α-helix versus β-sheet composition). The fingerprint region (1500–900 cm^−1^) (i,j,k,l,m,n)—CH_2_ bending (~1465 cm^−1^) and CH_3_ bending (~1377 cm^−1^). Phosphate vibrations (~1240–1230 cm^−1^; associated with nucleic acids/phospholipids). C–O stretching vibrations (1200–1000 cm^−1^) from carbohydrates, glycogen, and phospholipids. Subtle yet consistent differences between the classes are observed, particularly around 1240 cm^−1^ and 1080 cm^−1^, which are frequently noted as discriminant regions in studies of pork authenticity [3,9].

### 3.2. Principle Component Analysis

**Figure 2:**
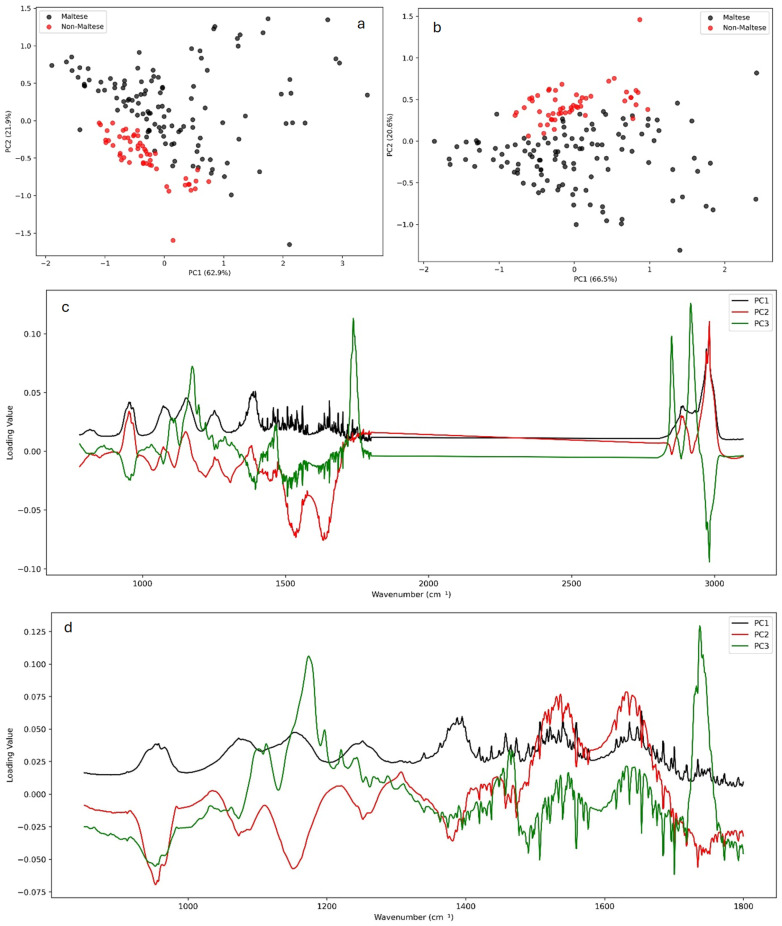
PCA of ATR-FTIR spectra for Maltese (black) and non-Maltese (red) pork. Score plots based on the whole spectrum (**a**) and the fingerprint region (**b**) show clustering between groups, while corresponding loadings (**c**,**d**) highlight key discriminatory bands in proteins, lipids, and carbohydrates. Full PCA results for all preprocessing transformations are provided in the Supplementary Information (Figures S2–S5).

The Principal Component Analysis (PCA) of the ATR-FTIR spectra revealed clear clustering trends between Maltese and non-Maltese pork samples. Using the whole spectral range (4000–650 cm^−1^), Maltese samples grouped apart from non-Maltese, though with some overlap (Figure 2a). Restricting the analysis to the fingerprint region (1800–850 cm^−1^) improved the separation, indicating that biochemical signatures within this range provide greater discriminatory power (Figure 2b). The examination of the PCA loadings (Figure 2c,d) highlighted the spectral variables most responsible for class differentiation, including protein-associated Amide I and II bands (1700–1500 cm^−1^), lipid CH_2_ and CH_3_ stretching/bending modes (3000–2800 and ~1460 cm^−1^), and carbohydrate/phosphate-related vibrations (1200–900 cm^−1^). These regions have previously been reported [7,10] as key markers for meat authentication, where lipid carbonyl (~1745 cm^−1^) and amide absorptions are particularly sensitive to the species origin and processing [3,4,9]. The stronger discriminatory power of the fingerprint region is consistent with earlier studies, showing that multivariate models based on the 1800–900 cm^−1^ yield enhanced the classification of pork, beef, and poultry products. Overall, the separation observed here reflects underlying biochemical differences in muscle protein secondary structures and lipid distribution between Maltese and non-Maltese pork, in agreement with published FTIR–chemometric studies on meat speciation. Full PCA results for all preprocessing transformations are provided in the Supplementary Information (Figures S2–S5).

Table 2 shows the proportion of variance explained by the first two components (PC1 and PC2) which varied considerably depending on the transformation. For the fingerprint region, the 2nd Savitzky–Golay derivative yielded the highest explained variance (PC1 = 95.5%, PC2 = 1.7%), followed by the 1st derivative (PC1 = 89.1%, PC2 = 3.3%). Other transformations such as Deresolve, the Median filter, and Raw spectra explained moderate variance (PC1 ~66%), whereas Detrend, MSC, SNV, and Quantile normalization performed less strongly (PC1 = 53–56%).

A similar pattern was observed for the whole spectrum, where the 2nd derivative again explained the highest variance (PC1 = 92.4%) although lower than the fingerprint. These results confirm that derivative-based preprocessing coupled with the use of the fingerprint region maximizes the discriminatory information in the pork FTIR spectra, particularly within the fingerprint region, where subtle biochemical differences between Maltese and non-Maltese samples were best captured.

### 3.3. The Soft Independent Modeling of Class Analogy (SIMCA)

The SIMCA classification models shown in Table 3 demonstrated a consistently high performance across both whole and fingerprint spectral regions. In the whole spectrum, several preprocessing methods including Deresolve, Detrend, OSC, Raw, Median Filter, and Quantile Normalization achieved 100% excluded sample accuracy, with Deresolve emerging as the most efficient transformation given the lowest number of outliers (n = 5). Similarly, the fingerprint spectra produced excellent results, with the 2nd derivative achieving perfect classification (100%), although at the cost of seven removed outliers. Outlier analysis using Hotelling’s T^2^ plots and Q residuals can be found in Supplementary Material Figures S6 and S7. Detrend provided a more balanced outcome, with a high excluded accuracy (98.2%) and the lowest outlier count (n = 4). Overall, while both spectral ranges yielded strong discrimination between Maltese and foreign pork, the whole spectrum offered more routes to perfect classification, whereas the fingerprint region, although slightly less robust, highlighted the discriminative power of derivative-based preprocessing. A representative analysis of the specificity, selectivity, and accuracy can be found in Table 3 and is visualized in Figure S9.

**Figure 3:**
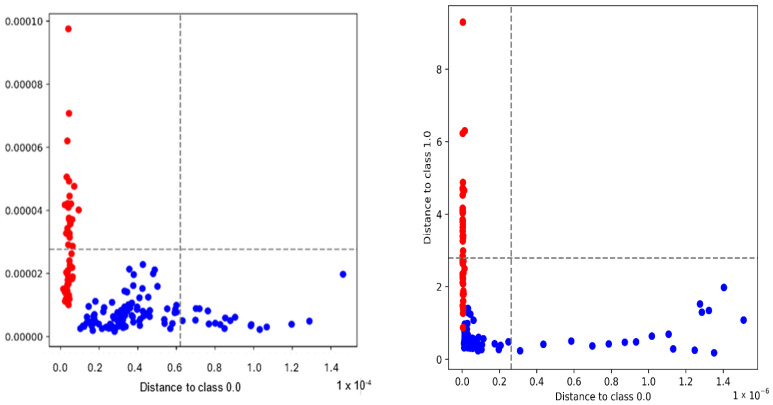
Coomans plots of SIMCA classification models. (**Left**) Whole spectrum after Deresolve preprocessing, showing the separation between Maltese (red) and foreign pork samples (blue). (**Right**) Fingerprint spectrum after 2nd derivative preprocessing, illustrating improved class separation with minimal overlap between classes.

The representative Coomans plots illustrated in Figure 3 and Figure S8 show the discriminatory performance of SIMCA models for Maltese and foreign pork samples. In the whole spectrum with Deresolve preprocessing (left), a clear separation is observed, with the majority of samples correctly clustered within their respective class boundaries and only a few outliers detected. The fingerprint spectrum with 2nd-derivative preprocessing (right) further enhanced the resolution between classes, as indicated by the sharper distinction between Maltese (red) and foreign (blue) samples, although a slightly higher sensitivity to outliers was noted. These results confirm that both preprocessing strategies provided effective class modeling, with the fingerprint region offering the improved interpretability of biochemical variation despite increased outlier sensitivity.

### 3.4. Multivariate Classification Using PCA-LDA and PLS-LDA

**Figure 4:**
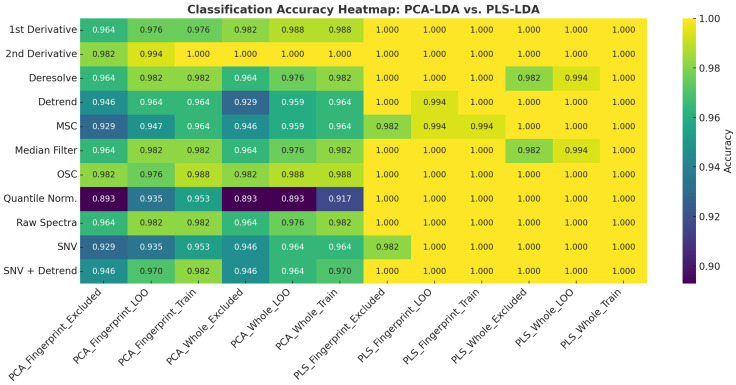
Heatmap visualization of classification accuracies for PCA-LDA and PLS-LDA models applied to whole and fingerprint FTIR spectra of pork samples under different preprocessing methods. Each cell represents the classification accuracy for training, leave-one-out (LOO), or excluded validation sets. Darker shades indicate lower accuracies, while yellow regions indicate perfect classification (100%).

**Figure 5:**
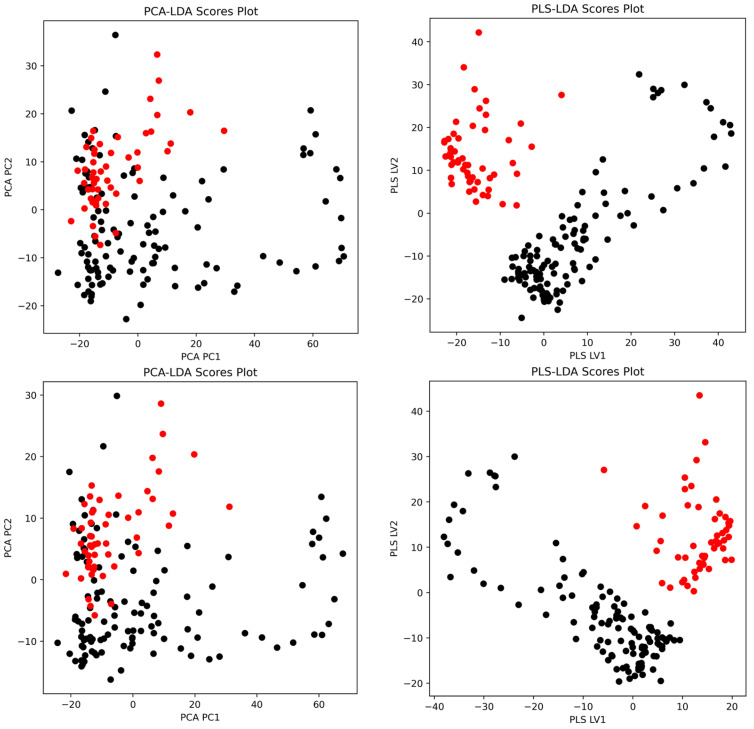
Representative classification score plots comparing PCA-LDA and PLS-LDA models for pork authentication. Top row: (**left**) PCA-LDA scores plot using second-derivative whole spectrum and (**right**) PLS-LDA scores plot using 2nd-derivative whole spectrum. Bottom row: (**left**) PCA-LDA scores plot using second-derivative fingerprint region and (**right**) PLS-LDA scores plot using second-derivative fingerprint region. Black points represent Maltese pork samples and red points represent non-Maltese pork samples.

The classification performances of the PCA-LDA and PLS-LDA models for pork authentication using FTIR spectral data are summarized and visualized in Figure 4, Figure 5, and Figure S10. Across all preprocessing methods, PLS-LDA achieved perfect accuracies (100%) for both the whole spectra and fingerprint regions, regardless of training, leave-one-out (LOO), or excluded validation sets. In contrast, PCA-LDA yielded slightly lower accuracies depending on the preprocessing method. For example, quantile normalization reduced the PCA-LDA performance (whole: 91.7% training, 89.3% LOO, 89.3% excluded), whereas first and second derivatives improved the classification (≥98% across all datasets). SNV and detrending combinations also maintained accuracies above 94%. These results highlight that PLS-LDA consistently outperformed PCA-LDA in terms of their robustness, particularly when dealing with excluded validation samples.

The classification scores plots (Figure 5) demonstrate the separation achieved by PCA-LDA and PLS-LDA using second-derivative spectral preprocessing. For the whole spectral range, PCA-LDA showed a partial overlap between Maltese (black) and non-Maltese (red) pork samples, whereas PLS-LDA achieved clearer class separation along the first two latent variables. A similar trend was observed in the fingerprint region, where PLS-LDA provided a more distinct clustering pattern compared to PCA-LDA, confirming its superior discriminatory power.

### 3.5. Partial Least Squares Regression (PLSR)

Partial Least Squares Regression (PLSR) analysis was conducted on both the whole FTIR spectra and the fingerprint region across a range of preprocessing methods (Table 4). The results demonstrated a strong predictive performance with coefficients of determination (R^2^) exceeding 0.95 for most transformations. Among the whole-spectrum models, the 1st derivative (R^2^ = 0.993, RMSE Train = 0.039) and 2nd derivative (R^2^ = 0.988, RMSE Train = 0.050) exhibited the highest calibration performance with relatively low excluded-sample error values (0.081 and 0.076, respectively). For the fingerprint region, the 2nd derivative achieved the strongest performance (R^2^ = 0.996, RMSE Train = 0.029), indicating that this spectral domain captured the most discriminative information. Conversely, quantile normalization and SNV + Detrend produced lower R^2^ values (<0.90 in the whole spectra), reflecting a weaker predictive capacity compared to derivative and smoothing-based approaches. Overall, derivative preprocessing methods (1st and 2nd derivative) consistently enhanced the calibration accuracy and model robustness across both spectral ranges.

Across all preprocessing methods, the root mean square error of cross-validation (RMSE LOO) values were consistently higher than the corresponding training and excluded-sample errors. LOO-CV generally yields higher RMSE than external test sets because each sample is predicted by a model trained without it, providing a stricter estimate of generalization error [21]. In contrast, holdout partitions can underestimate the error if test samples remain correlated with the training set [21]. Moreover, the high overlap between training folds in LOO increases the variance in error estimates, inflating RMSE compared with independent validation [20].

**Figure 6:**
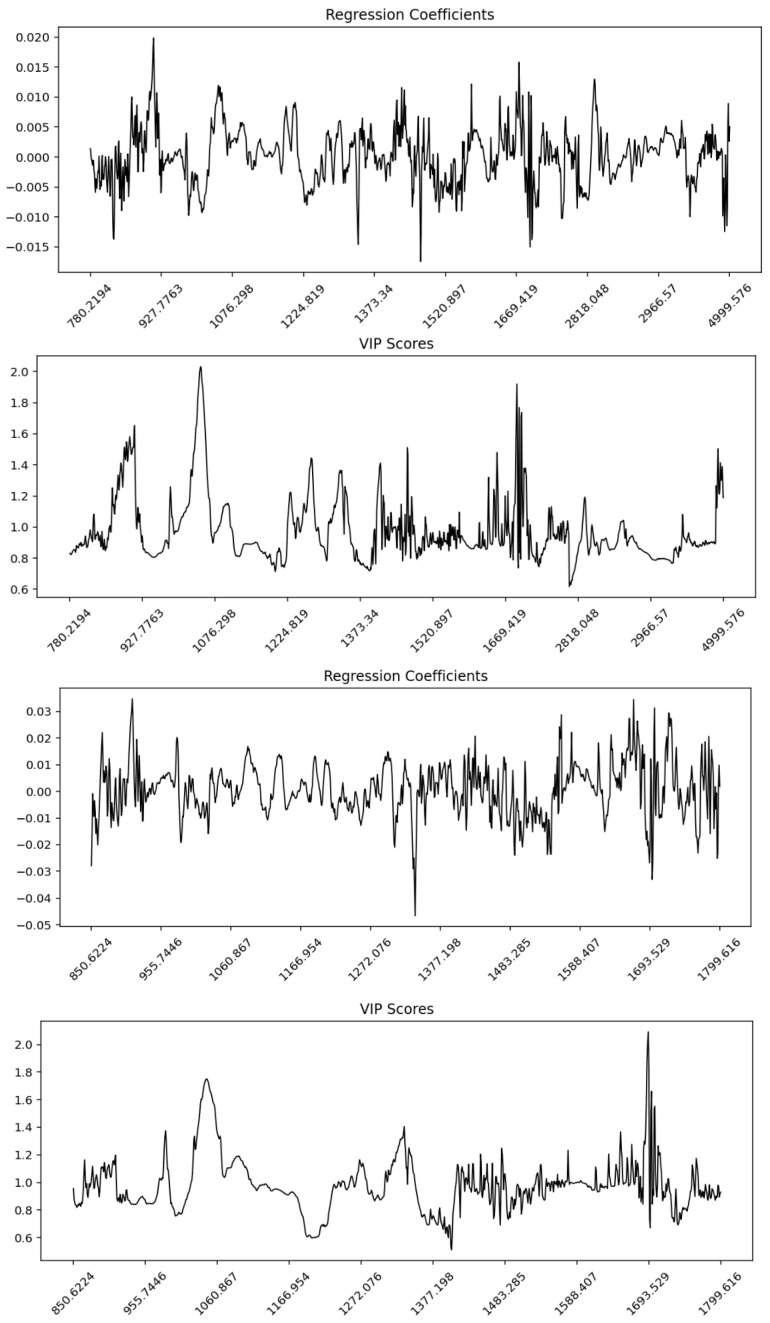
Partial Least Squares Regression (PLSR) regression coefficients and Variable Importance in Projection (VIP) profiles for Maltese and non-Maltese pork samples based on ATR-FTIR spectra. (**top panels**) correspond to the whole spectral range (4000–650 cm^−1^) and (**bottom panels**) to the fingerprint region (1800–850 cm^−1^). The regression coefficient plots indicate the relative contribution and direction of each wavenumber toward class prediction, while the VIP score plots highlight the most influential spectral variables (VIP > 1). Key discriminative bands are observed around 1650 cm^−1^ (Amide I), 1540 cm^−1^ (Amide II), 1200–1000 cm^−1^ (carbohydrate/phosphate vibrations), and 2950–2850 cm^−1^ (CH_2_/CH_3_ lipid stretching), confirming that protein, lipid, and phospholipid structures drive the differentiation between pork origins.

The β-regression coefficient and Variable Importance in Projection (VIP) plots for the whole spectrum and fingerprint region revealed distinct regions contributing most strongly to the PLSR models (Figure 6 and Figures S11–S13). In the whole spectrum, the regression coefficients showed distributed contributions across the mid-infrared range, with higher weights observed in the lipid-associated CH stretching region (~3000–2800 cm^−1^) and the protein-related Amide I and II bands (~1700–1500 cm^−1^). The VIP plot highlighted sharp peaks above the threshold (VIP > 1) in these same regions, indicating their importance for discrimination. In the fingerprint region, both regression coefficients and VIP scores emphasized bands associated with protein secondary structures (Amide I and II) and carbohydrate/lipid vibrations in the 1200–900 cm^−1^ range. These findings suggest that the fingerprint region provided more localized discriminative information compared to the broader distribution observed in the whole spectrum.

### 3.6. Support Vector Machine Regression (SVMR) Modeling

Support Vector Machine Regression (SVMR) models were developed for both the whole FTIR spectra and the fingerprint region under different preprocessing conditions (Table 5). In general, the models showed an extremely high predictive performance, with R^2^ values exceeding 0.97 across all methods and reaching 0.9995–0.9996 for most preprocessing strategies. The 1st and 2nd derivative methods consistently provided the lowest training errors (RMSE Train ≈ 0.009–0.010) and strong generalization, with excluded-sample errors as low as 0.081 in the fingerprint region. Conversely, MSC, SNV, and quantile normalization yielded higher cross-validation errors (RMSE LOO > 0.15) and larger excluded-sample deviations (>0.18), indicating reduced robustness despite excellent calibration fits. The OSC pre-processed models showed a stable performance, particularly in the fingerprint region, where RMSE Excluded was as low as 0.104.

**Figure 7:**
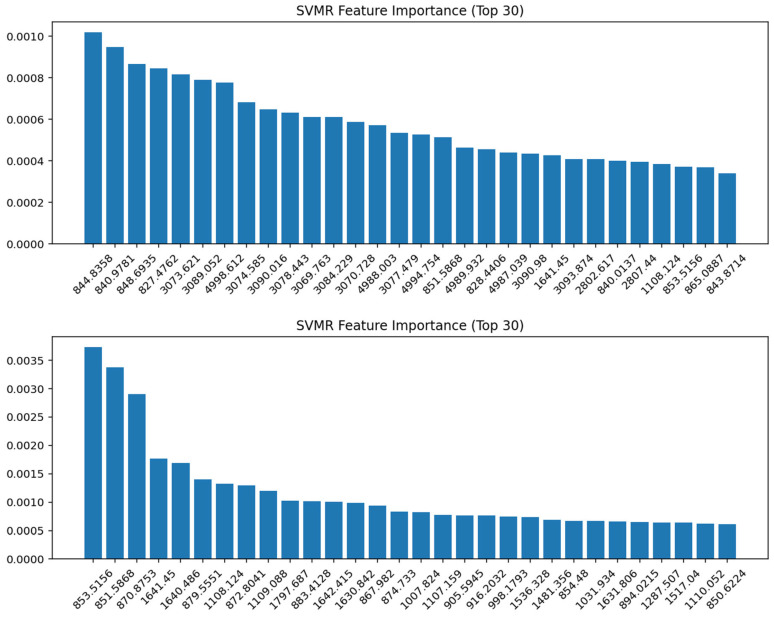
SVMR feature importance (Top 30 variables) for the 1st derivative spectra. (**Top panel**): whole spectrum; (**bottom panel**): fingerprint region. The most discriminative variables were observed around 840–870 cm^−1^, with additional contributions in the Amide I region (1640–1650 cm^−1^) and polysaccharide/phospholipid bands near 1100 cm^−1^, highlighting the biochemical basis for model discrimination.

Feature importance analysis of the SVMR models identified the spectral variables contributing most strongly to the prediction (represented by the 1st derivative in Figure 7 whilst the remaining transformations are presented in Figure S14). For the whole spectrum (top panel), the most influential features were located in the lower wavenumber region around 840–850 cm^−1^, with additional contributions spanning 3000–3100 cm^−1^. In the fingerprint region (bottom panel), the most important wavenumbers were also concentrated in the 850–870 cm^−1^ range, along with clear contributions from the protein Amide I–II region (1640–1650 cm^−1^) and several bands around 1100 cm^−1^ and 1500–1600 cm^−1^. These findings indicate that SVMR placed a higher weight on fine-scale vibrational features within the fingerprint region compared to the broader distribution observed in the full spectrum.

### 3.7. Artificial Neural Network (ANN) Modeling

Artificial Neural Network (ANN) models were developed on all spectral transformations to assess their capacity for pork authentication (Table 6). Training accuracies were generally high, with several preprocessing methods achieving near-perfect performance. The 2nd derivative model achieved the strongest training performance (Accuracy = 1.000, AUC = 1.000), followed by SNV (Accuracy = 0.988, AUC = 0.999) and OSC (Accuracy = 0.976, AUC = 0.998). In contrast, MSC and detrend methods yielded lower calibration accuracies (<0.91) and higher misclassification rates (>9%). Validation on excluded samples showed more variability. The OSC (Accuracy = 0.965, AUC = 0.996) and Median Filter (Accuracy = 0.947, AUC = 0.999) models maintained high predictive accuracy with relatively low misclassification rates (<6%). Conversely, raw and deresolve preprocessing performed less effectively, with excluded accuracies around 0.77 and misclassification rates exceeding 22%. These results demonstrate that derivative-based, OSC, and median-filter preprocessing provided the most robust ANN models, while simple or normalization-only approaches were less effective.

**Figure 8:**
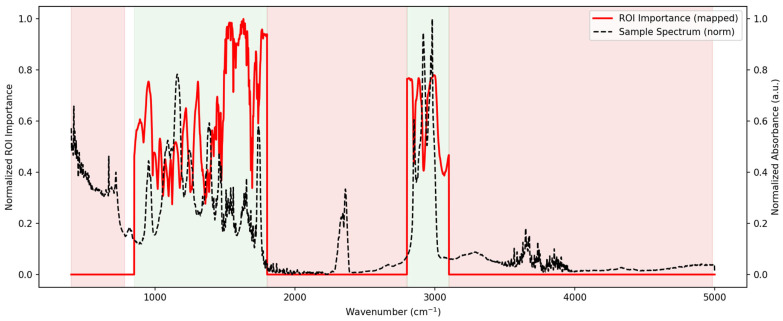
Region of Importance (ROI) mapping for the ANN model applied to pork authentication. The black dashed line represents a normalized mean spectrum, while the red trace indicates normalized ROI importance across the wavenumber axis. Red parts of the spectrum are the regions excluded; grey areas are the regions included in the analysis.

The region of importance (ROI) mapping represented in Figure 8 by a normalized spectrum and Figure S17 obtained from the ANN models highlighted the spectral intervals contributing most strongly to the classification between Maltese and non-Maltese pork. The confusion matrices are also represented in Figure S15, together with the Receiver Operating Characteristic (ROC) curves presented in Figure S16. The fingerprint region (~900–1800 cm^−1^) showed the highest concentration of discriminative features, particularly within the protein Amide I–II bands (~1650 and ~1550 cm^−1^) and carbohydrate/lipid-associated vibrations between 1000 and 1200 cm^−1^. Additional peaks of relevance were observed around the lipid CH stretching bands (2800–3000 cm^−1^), while regions above 3500 cm^−1^ contributed minimally. These findings confirm that the ANN relied on chemically meaningful features within the mid-infrared spectrum for accurate discrimination.

## 4. Discussion

The ATR-FTIR spectra revealed clear biochemical differences between Maltese and non-Maltese pork across protein-, lipid-, ester-, and carbohydrate-associated regions previously identified by other authors [3,4,6]. In the high wavenumber region, the broad Amide A band (~3290 cm^−1^), corresponding to the N–H stretching of proteins with O–H contributions from polysaccharides, appeared slightly more intense in Maltese pork; however, due to the possible water overlap, this peak was excluded from the analysis. In the lipid region (3000–2800 cm^−1^), Maltese pork displayed more pronounced CH_3_ and CH_2_ stretching vibrations. Both the CH_3_ asymmetric stretching (~2956 cm^−1^) and the CH_2_ asymmetric stretching (~2925 cm^−1^) bands were stronger, as was the CH_2_/CH_3_ symmetric stretching region (~2872–2853 cm^−1^). These peaks reflect intramuscular lipids, phospholipids, and neutral lipids, indicating that Maltese pork exhibits relatively stronger methyl and methylene vibrational contributions [3,4,5,7]. In contrast, non-Maltese pork exhibited stronger carbonyl and protein-related absorptions. The C=O stretching vibration at ~1715 cm^−1^, associated with fatty acids and aromatic esters, was more defined in non-Maltese samples, suggesting higher levels of free fatty acids or oxidation products [3,4,9]. Similarly, the Amide I (~1655 cm^−1^) and Amide II (~1540 cm^−1^) bands were more intense in non-Maltese pork, indicating higher contributions from structural proteins or differences in secondary structures [3,6,7]. This contrasts with the higher Amide A intensity observed in Maltese pork, suggesting possible differences in protein conformations or hydration states between the two groups [3]. Further differences were evident in the fingerprint region (1500–900 cm^−1^). Non-Maltese pork exhibited stronger CH_2_ bending vibrations around ~1465 cm^−1^, together with more intense signals in the ~1412–1418 cm^−1^ region associated with cis-olefinic rocking and C–N stretching. The COO^−^ symmetric stretching band at ~1392 cm^−1^, a marker for fatty acid composition, was also stronger in non-Maltese pork. These absorptions are consistent with a greater lipid bending intensity, higher fatty acid unsaturation, and compositional differences in fatty acid profiles [3,4,7].

In contrast, Maltese pork showed more pronounced signals in the Amide III region (~1315–1230 cm^−1^), which also overlaps with PO_2_^−^ asymmetric stretching from phospholipids and nucleic acids [3,4,7]. Additional differences were observed in the 1170–1150 cm^−1^ region, corresponding to the C–O stretching of serine, threonine, and tyrosine residues, and in the 1080–1030 cm^−1^ range, assigned to PO_2_^−^ symmetric stretching and C–O vibrations of carbohydrates and glycogen. These stronger absorptions in Maltese pork indicate a higher contribution from structural proteins, phospholipids, and carbohydrate-related biomolecules [3,7]. Taken together, these spectral observations suggest that Maltese pork is distinguished by stronger Amide A, Amide III, and phosphate/carbohydrate-associated vibrations, alongside pronounced CH_2_/CH_3_ stretching bands. Non-Maltese pork, on the other hand, is characterized by stronger Amide I–II absorptions, more defined carbonyl stretching, and greater lipid bending and fatty acid-associated peaks [7]. These compositional differences are likely rooted in production practices: Maltese pork, typically derived from small-scale systems with balanced feeding and shorter supply chains, shows stronger signatures of structural proteins and phospholipids, whereas non-Maltese pork, associated with intensive farming and energy-dense diets, exhibits higher levels of free fatty acids, lipid unsaturation, and protein signals linked to leaner carcass development.

Chemometric modeling confirmed that these spectral features formed the basis for robust classification. The application of Savitzky–Golay derivatives improved the resolution of overlapping peaks in the amide and lipid regions, allowing subtle yet systematic differences between the groups to be emphasized. The superior performance of second-derivative preprocessing in PCA clustering mirrors earlier findings in meat authenticity studies, where derivative treatments consistently enhanced separation [6,8,15]. Supervised classifiers further improved the classification accuracy. PLS-LDA achieved 100% accuracy across preprocessing methods, outperforming PCA-LDA, which does not explicitly optimize for class-related variance. This agrees with earlier studies showing that PLS-DA and SVM consistently outperform PCA-based models in meat species and origin authentication [6,15]. Although whole-spectrum models achieved high accuracy, the fingerprint region (1800–900 cm^−1^) emerged as the most chemically meaningful. It captures the amide bands, lipid bending modes, and phosphate/carbohydrate absorptions that directly reflect protein-to-lipid ratios and cellular composition. This reinforces the literature consensus that the fingerprint region provides the richest biochemical information for species and origin discrimination [6,7,8]. Nevertheless, second derivative models showed increased outlier sensitivity, suggesting that complementary preprocessing strategies such as detrend or OSC may offer a more stable balance between accuracy and robustness. These observations are summarized in Table 7, which compares the different preprocessing techniques applied in this study, highlighting their relative advantages, limitations, and impact on the spectral resolution and model performance.

Regression modeling further highlighted the discriminatory power of the fingerprint region. PLSR models performed best with derivative preprocessing, though inflated leave-one-out (LOO) errors reflected the known limitations of this validation strategy in small datasets. Nonlinear regression approaches such as SVMR provided stronger predictive robustness, capturing subtle biochemical patterns beyond the linear structure of PLSR. Feature importance from SVMR and region of importance from ANN consistently highlighted Amide I (~1650 cm^−1^), CH_2_/CH_3_ bending (~1465 cm^−1^), and carbohydrate/phosphate bands (~1117–1031 cm^−1^) as the most discriminative, fully matching the biochemical assignments of the spectra. ANN models also performed strongly when derivative or OSC preprocessing was applied, corroborating recent evidence that deep learning approaches enhance classification power in FTIR–chemometric workflows [6,8,15].

These results confirm that Maltese and non-Maltese pork can be reliably differentiated based on their FTIR fingerprints. Maltese pork is defined by stronger protein- and phosphate-associated absorptions, while non-Maltese pork is characterized by more pronounced lipid- and ester-associated signals. When coupled with derivative preprocessing and supervised classifiers, ATR-FTIR provides a rapid, non-destructive, and cost-effective strategy for pork origin authentication. Spectral acquisition required approximately 3 min per sample, with negligible reagent consumption, thereby offering a markedly more economical alternative to conventional molecular or proteomic approaches. DNA-based authentication (e.g., PCR or qPCR) typically entails 2–4 h of sample preparation, amplification, and analysis, in addition to recurring expenses for extraction kits and enzymes, while proteomic or mass-spectrometric methods frequently exceed these temporal and financial requirements [26]. Relative to such methods, ATR-FTIR reduces per-sample reagent and consumable costs by an estimated ≥70% and lowers total analytical expenditure to roughly 5–10% of that associated with a conventional workflow [27]. These findings are concordant with previous demonstrations of the robustness of FTIR–chemometric strategies for meat traceability and halal verification [6,7,14,26,27] and, together with reports of the successful deployment of portable ATR-FTIR instrumentation, highlight the feasibility of implementing this approach for rapid, on-site regulatory and industrial monitoring.

## 5. Conclusions

This study demonstrated the successful application of ATR-FTIR spectroscopy coupled with advanced chemometric and machine learning approaches for the authentication of Maltese versus non-Maltese pork. A comprehensive evaluation of classification and regression strategies revealed that data preprocessing plays a pivotal role in extracting chemically meaningful information from complex FTIR spectra. Derivative transformations, particularly the Savitzky–Golay first and second derivatives, consistently enhanced spectral resolution and improved model robustness across all workflows.

Linear models such as PCA-LDA, SIMCA, and PLSR provided high levels of accuracy and interpretability, with the fingerprint region (1800–600 cm^−1^) emerging as the most discriminative spectral domain due to its rich representation of proteins, lipids, and nucleic acids. However, these methods were more sensitive to sample variability and exhibited inflated errors under stringent cross-validation. Nonlinear approaches, especially Support Vector Machine Regression (SVMR) and Artificial Neural Networks (ANNs), delivered a superior predictive performance, with accuracies exceeding 0.99 and lower misclassification rates under external validation. The ANN models, when combined with appropriate preprocessing (2nd derivative, OSC, or median filtering), provided the most powerful classification framework, highlighting the capacity of deep learning to capture subtle, nonlinear spectral features.

Collectively, these findings confirm that FTIR spectroscopy coupled with chemometrics, and machine learning provides a rapid, cost-effective, and non-destructive tool for meat authenticity assessments. The strong performance of nonlinear models underscores their potential for real-world deployment in quality control and regulatory enforcement. Importantly, the results also emphasize that the careful choice of the preprocessing and validation strategy is essential to prevent overfitting and to ensure model generalizability.

## Figures and Tables

**Table 1:** Comparative ATR-FTIR spectra of Maltese (black) and foreign (red) pork, showing peak assignments (a–n) and relative intensity differences linked to lipids, proteins, and carbohydrates.

Peak	Description/Band Assignment	Functional Group/Bond	Wavenumber (cm^−1^)	Intensity Trend	Sample Origin Difference
a	Broad band	O–H/N–H stretch (water, proteins, lipids)	~3270–3300	Medium in both, slightly higher in foreign	Foreign pork shows slightly stronger water/protein broadening
b	Sharp band	C–H stretch (asym. CH_2_/CH_3_, lipids)	~2955	Strong, higher in Maltese	Maltese pork shows more pronounced lipid CH_2_/CH_3_ vibrations
c	Sharp band	C–H stretch (sym. CH_2_ lipids)	~2920	Strong in both	Comparable, but Maltese marginally stronger
d	Shoulder band	C–H stretch (sym. CH_3_, alkanes)	~2850	Medium, higher in Maltese	Indicates higher lipid/methylenic content in Maltese pork
e	Strong band	C=O stretch (Amide I, proteins)	~1650	Very strong in both	No major difference—protein backbone vibration
f	Medium band	Amide II (N–H bend and C–N stretch, proteins)	~1540	Medium, higher in Maltese	Maltese samples have stronger amide II band
g	Shoulder	Amide III (C–N, N–H bend, proteins)	~1450	Weak–Medium	Slightly stronger in foreign samples
h	Sharp band	CH_2_ scissoring (lipids)	~1400	Medium, higher in Maltese	Suggests higher lipid content
i	Weak band	COO^−^ sym. stretch (fatty acids)	~1330	Weak in both	No clear origin difference
j	Medium band	Amide III/C–N stretch (proteins)	~1240	Medium, slightly higher in foreign	Foreign pork shows stronger protein secondary structure vibration
k	Medium band	P=O stretch (phospholipids, nucleic acids)	~1080	Medium, slightly higher in Maltese	Maltese has stronger phospholipid features
l	Shoulder	C–O stretch (carbohydrates, collagen)	~1030	Weak–Medium, higher in foreign	Foreign pork shows stronger polysaccharide/collagen bands
m	Sharp band	C–O–C stretch (polysaccharides, lipids)	~970	Weak in both	Subtle, foreign slightly higher
n	Fingerprint vibration	C–H out-of-plane bend (aromatics)	~920	Weak, stronger in foreign	Foreign pork richer in aromatic vibrations

**Table 2:** Explained variation (%) of the first two principal components (PC1 and PC2) for different spectral pre-processing methods applied to ATR-FTIR spectra of pork (whole spectrum: 4000–650 cm^−1^; fingerprint region: 1800–850 cm^−1^), sorted by PC1 Fingerprint.

Pre-Processing Method	PC1 Whole (%)	PC2 Whole (%)	PC1 Fingerprint (%)	PC2 Fingerprint (%)
2nd SG derivative	92.4	3.5	95.5	1.7
1st SG derivative	79.4	10.1	89.1	3.3
Deresolve	63.1	22.0	66.8	20.7
Median filter	62.8	22.3	66.5	24.1
Raw spectra	62.9	21.9	66.5	20.6
OSC	64.5	16.4	59.1	13.6
SNV + Detrend	57.7	29.1	57.4	23.6
SNV	57.7	28.7	54.9	23.8
MSC	63.1	25.3	55.2	23.1
Detrend	48.6	32.3	55.6	28.1
Quantile normalization	55.6	28.4	53.2	25.0

**Table 3:** SIMCA classification performance.

**(a) Whole Spectrum**						
**Pre-Processing Method**	**Training Accuracy**	**Specificity**	**Selectivity**	**LOO Accuracy**	**Excluded Accuracy**	**Outliers Removed**
1st Derivative	0.963	0.946	1.000	0.951	0.889	6
2nd Derivative	0.970	0.964	0.981	0.927	0.796	6
Deresolve	1.000	1.000	1.000	1.000	1.000	5
Detrend	1.000	1.000	1.000	1.000	1.000	7
Median Filter	0.994	0.991	1.000	0.988	1.000	6
MSC	0.982	0.973	1.000	0.982	0.926	6
OSC	1.000	1.000	1.000	1.000	1.000	8
Quantile Norm.	0.981	0.973	1.000	0.957	1.000	8
Raw	1.000	1.000	1.000	1.000	1.000	6
SNV	0.988	0.982	1.000	0.970	0.963	6
SNV + Detrend	0.988	0.982	1.000	0.982	0.944	6
**(b) Fingerprint spectrum**						
**Pre-processing Method**	**Training Accuracy**	**Specificity**	**Selectivity**	**LOO Accuracy**	**Excluded Accuracy**	**Outliers Removed**
1st Derivative	0.970	0.955	1.000	0.957	0.981	6
2nd Derivative	1.000	1.000	1.000	1.000	1.000	7
Deresolve	0.994	0.991	1.000	0.982	0.964	5
Detrend	0.994	0.991	1.000	0.976	0.982	4
Median Filter	0.994	0.991	1.000	0.982	0.964	5
MSC	0.939	0.911	1.000	0.939	0.926	6
OSC	0.994	0.991	1.000	0.970	0.982	5
Quantile Norm.	0.927	0.893	1.000	0.896	0.944	6
Raw	0.994	0.991	1.000	0.982	0.964	5
SNV	0.927	0.893	1.000	0.927	0.944	6
SNV + Detrend	0.939	0.910	1.000	0.933	0.963	7

**Table 4:** PLSR regression performance.

**(a) Whole Spectrum**					
**Pre-Processing Method**	**R^2^**	**LV**	**RMSE Train**	**RMSE LOO**	**RMSE Excluded**
1st Derivative	0.993	13	0.039	0.604	0.081
2nd Derivative	0.988	7	0.050	0.611	0.076
Deresolve	0.957	15	0.096	0.591	0.101
Detrend	0.964	15	0.089	0.619	0.115
Median Filter	0.959	15	0.094	0.586	0.104
MSC	0.919	14	0.132	0.620	0.154
OSC	0.962	14	0.090	0.581	0.103
Quantile Norm.	0.859	10	0.173	0.622	0.316
Raw	0.960	15	0.094	0.591	0.103
SNV	0.932	15	0.121	0.609	0.155
SNV + Detrend	0.885	10	0.157	0.603	0.185
**(b) Fingerprint spectrum**					
**Pre-Processing Method**	**R^2^**	**LV**	**RMSE Train**	**RMSE LOO**	**RMSE Excluded**
1st Derivative	0.970	10	0.080	0.611	0.110
2nd Derivative	0.996	12	0.029	0.594	0.083
Deresolve	0.956	15	0.098	0.574	0.117
Detrend	0.964	15	0.088	0.598	0.127
Median Filter	0.964	15	0.089	0.577	0.141
MSC	0.910	13	0.139	0.620	0.174
OSC	0.965	15	0.087	0.572	0.129
Quantile Norm.	0.925	13	0.127	0.638	0.188
Raw	0.960	15	0.093	0.581	0.116
SNV	0.912	13	0.137	0.612	0.179
SNV + Detrend	0.906	12	0.142	0.603	0.180

**Table 5:** SVMR regression performance.

**(a) Whole Spectrum**				
**Pre-Processing Method**	**R^2^**	**RMSE Train**	**RMSE LOO**	**RMSE Excluded**
1st Derivative	0.9996	0.0096	0.104	0.112
2nd Derivative	0.9996	0.0097	0.089	0.081
Deresolve	0.9754	0.0730	0.118	0.123
Detrend	0.9996	0.0097	0.138	0.176
Median Filter	0.9759	0.0723	0.119	0.119
MSC	0.9996	0.0098	0.171	0.213
OSC	0.9996	0.0096	0.117	0.116
Quantile Norm.	0.9996	0.0095	0.159	0.202
Raw	0.9754	0.0729	0.118	0.098
SNV	0.9996	0.0097	0.179	0.209
SNV + Detrend	0.9996	0.0098	0.147	0.164
**(b) Fingerprint spectrum**				
**Pre-Processing Method**	**R^2^**	**RMSE Train**	**RMSE LOO**	**RMSE Excluded**
1st Derivative	0.9996	0.0096	0.109	0.129
2nd Derivative	0.9996	0.0094	0.095	0.081
Deresolve	0.9741	0.0745	0.129	0.133
Detrend	0.9697	0.0802	0.168	0.183
Median Filter	0.9747	0.0742	0.127	0.147
MSC	0.9995	0.0098	0.171	0.180
OSC	0.9798	0.0658	0.123	0.104
Quantile Norm.	0.9996	0.0097	0.161	0.264
Raw	0.9748	0.0740	0.128	0.176
SNV	0.9996	0.0098	0.177	0.194
SNV + Detrend	0.9996	0.0098	0.140	0.188

**Table 6:** ANN performance.

**(a) Training Set**								
**Pre-Processing**	**Accuracy**	**Precision**	**Recall**	**Specificity**	**F1 Score**	**Misclass. (%)**	**Entropy**	**AUC**
1st Derivative	0.976	0.975	0.991	0.943	0.983	2.37	0.123	0.998
2nd Derivative	1.000	1.000	1.000	1.000	1.000	0.00	0.098	1.000
Deresolve	0.970	0.983	0.974	0.962	0.978	2.96	0.097	0.997
Detrend	0.905	0.903	0.966	0.774	0.933	9.47	0.333	0.973
Median Filter	0.964	0.974	0.974	0.943	0.974	3.55	0.097	0.996
MSC	0.858	0.890	0.905	0.755	0.897	14.2	0.323	0.932
OSC	0.976	1.000	0.966	1.000	0.982	2.37	0.071	0.998
Quantile Norm.	0.905	0.955	0.905	0.906	0.929	9.47	0.324	0.946
Raw	0.964	0.974	0.974	0.943	0.974	3.55	0.101	0.996
SNV	0.988	0.991	0.991	0.981	0.991	1.18	0.081	0.999
SNV + Detrend	0.941	0.982	0.931	0.962	0.956	5.92	0.205	0.989
**(b) Excluded validation set**								
**Pre-Processing**	**Accuracy**	**Precision**	**Recall**	**Specificity**	**F1 Score**	**Misclass. (%)**	**Entropy**	**AUC**
1st Derivative	0.935	0.905	0.974	0.778	0.938	8.77	0.230	0.962
2nd Derivative	0.930	0.907	1.000	0.778	0.951	7.02	0.296	0.944
Deresolve	0.772	0.906	0.744	0.833	0.817	22.8	0.388	0.919
Detrend	0.877	0.881	0.949	0.722	0.914	12.3	0.366	0.942
Median Filter	0.947	0.929	1.000	0.833	0.963	5.26	0.118	0.999
MSC	0.876	1.000	0.872	1.000	0.932	8.77	0.209	0.969
OSC	0.965	0.974	0.944	0.944	0.974	3.51	0.092	0.996
Quantile Norm.	0.882	0.946	0.897	0.889	0.921	10.5	0.256	0.969
Raw	0.772	0.882	0.769	0.778	0.822	22.8	0.323	0.927
SNV	0.895	0.923	0.923	0.833	0.923	10.5	0.375	0.899
SNV + Detrend	0.947	0.974	0.949	0.944	0.961	5.26	0.155	0.994

**Table 7:** Comparison of spectral preprocessing techniques and their impact on FTIR–chemometric analysis [22,23,24,25].

Preprocessing Method	Main Purpose	Advantages Observed	Limitations
1st Derivative	Enhances resolution, reduces baseline	Improved clustering in PCA, high accuracy in PLS-LDA and PLSR	Sensitive to noise if not smoothed
2nd Derivative	Resolves overlapping bands, emphasizes subtle features	Best performance in PCA (PC1 > 95% variance), highest PLSR and ANN accuracy, improved separation in SIMCA	More outliers, higher sensitivity
Deresolve	Derivative + smoothing	Perfect SIMCA classification (100%), balanced performance	Slightly lower variance explained compared to derivatives
Detrend	Removes baseline shifts	Stable classification in SIMCA and PLSR, fewer outliers	Slightly lower variance capture
Median Filter	Reduces high-frequency noise	Robust ANN performance, moderate PCA variance explained	May over-smooth subtle peaks
MSC	Corrects scatter effects	Stable results in SIMCA and regression	Higher excluded RMSE in some models
OSC	Removes variance unrelated to class	Robust ANN and SVMR performance, balanced classification	May overcorrect in small datasets
SNV	Corrects scatter and pathlength	High accuracy in ANN and SIMCA	Moderate variance explained in PCA
SNV + Detrend	Combination of scaling + baseline correction	Balanced reproducibility, fewer misclassifications	Not as strong as derivatives for PCA
Quantile Normalization	Standardizes distributions	Useful for comparability	Lowest variance explained, weaker classification
Raw (no preprocessing)	Baseline reference	Still yielded strong SIMCA classification (100%)	Less robust compared to derivative-based preprocessing

## Data Availability

The data presented in this study are available on request from the corresponding author. The FTIR spectral datasets generated and analyzed during the current study are not publicly archived due to ongoing project work, but can be made available upon reasonable request for academic purposes. No additional publicly archived datasets were generated.

## Supplementary Materials

The following supporting information can be downloaded at: https://www.mdpi.com/article/10.3390/foods14203510/s1, Figure S1. Representative ATR-FTIR spectra of Maltese pork (black) and non-Maltese pork (red) after applying different spectral transformations. Rows correspond to preprocessing methods: Raw, 1st Derivative, 2nd Derivative, Deresolve, Detrend, Median Filter, Multiplicative Scatter Correction (MSC), Orthogonal Signal Correction (OSC), Quantile Normalization, Standard Normal Variate (SNV), and SNV + Detrend. Figure S2. PCA score plots of the full spectrum (4000–400 cm^−1^) for all spectral transformations. Figure S3. PCA loading plots of the full spectrum (4000–400 cm^−1^) for all spectral transformations. Figure S4. PCA score plots of the fingerprint region (1800–850 cm^−1^) for all spectral transformations. Figure S5. PCA loading plots of the fingerprint region (1800–850 cm^−1^) for all spectral transformations. Figure S6. Outlier analysis using Hotelling’s T^2^ plots for the whole spectrum (right) and fingerprint region (left) across all spectral transformations. Figure S7. Q-residual plots for the whole spectrum (right) and fingerprint region (left) across all spectral transformations. Figure S8. Coomans plots (Q-residuals vs. Hotelling’s T^2^) for all spectral transformations, comparing the whole spectrum (right) and the fingerprint region (left). Figure S9. SIMCA classification performance metrics (accuracy and specificity) comparing the whole spectrum and fingerprint region across different spectral transformations. Figure S10. PLS-DA score plots for the whole spectrum (right) and fingerprint region (left) across all spectral transformations. Figure S11. β-regression coefficient plots of the full spectrum (4000–400 cm^−1^) across all spectral transformations. Figure S12. β-regression coefficient plots of the fingerprint region across all spectral transformations. Figure S13. Variable Importance in Projection (VIP) plots of the fingerprint region for all spectral transformations, contrasting Maltese (black) and non-Maltese (red) pork. Figure S14. Top 30 feature importance plots for the whole spectrum (left) and fingerprint region (right) across all spectral transformations. Figure S15. Confusion matrices for classification of Maltese and non-Maltese pork using ANN models with different preprocessing methods. Figure S16. Receiver Operating Characteristic (ROC) curves with area-under-the-curve (AUC) values for ANN classification of Maltese vs. non-Maltese pork across preprocessing methods. Figure S17. Region of Importance (ROI) plots showing the most influential wavenumber regions for discrimination between Maltese and non-Maltese pork.

## Abbreviations

The following abbreviations are used in this manuscript:

Abbreviation	Definition
ANN	Artificial Neural Network
ATR	Attenuated Total Reflectance
AUC	Area Under the Curve
ELISA	Enzyme-Linked Immunosorbent Assay
FTIR	Fourier Transform Infrared Spectroscopy
IR	Infrared
KIM	Koperattiva Industijali tal-Majjal
LDA	Linear Discriminant Analysis
LOOCV (or LOO)	Leave-One-Out Cross-Validation
LV	Latent Variable
MIR	Mid-Infrared
MLP	Multilayer Perceptron
MSC	Multiplicative Scatter Correction
OSC	Orthogonal Signal Correction
PCA	Principal Component Analysis
PCA-LDA	Principal Component Analysis–Linear Discriminant Analysis
PCR	Polymerase Chain Reaction
PLS	Partial Least Squares
PLS-DA	Partial Least Squares–Discriminant Analysis
PLS-LDA	Partial Least Squares–Linear Discriminant Analysis
PLSR	Partial Least Squares Regression
RMSE	Root Mean Square Error
ROC	Receiver Operating Characteristic
SIMCA	Soft Independent Modeling of Class Analogy
SNV	Standard Normal Variate
SVM	Support Vector Machine
SVMR	Support Vector Machine Regression
VIP	Variable Importance in Projection
