# Supplementary material for: Authentication of Maltese Pork Meat Unveiling Insights Through ATR-FTIR and Chemometric Analysis
Source: Foods. 2025 Oct 15;14(20):3510. doi: 10.3390/foods14203510 (PMC12564418; doi:10.3390/foods14203510)
Supplement: Supplementary file 1 [file foods-14-03510-s001.zip › foods-3882582-supplementary.pdf]

Supplementary material

Figure S1: Representative ATR-FTIR spectra of Maltese pork (black) and non-Maltese pork (red) after applying different spectral transformations. Rows correspond to preprocessing methods: Raw, 1st Derivative, 2nd Derivative, Deresolve, Detrend, Median Filter, Multiplicative Scatter Correction (MSC), Orthogonal Signal Correction (OSC), Quantile Normalization, Standard Normal Variate (SNV), and SNV+Detrend.

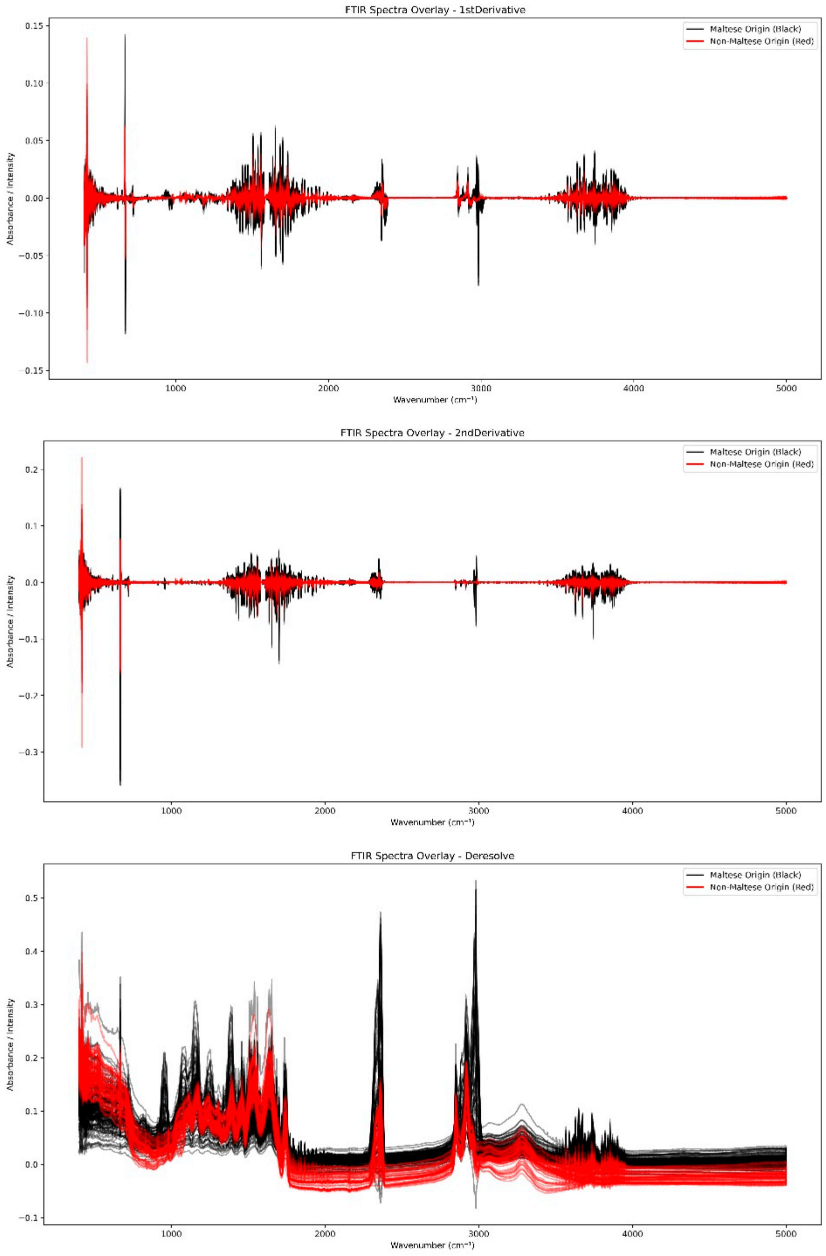

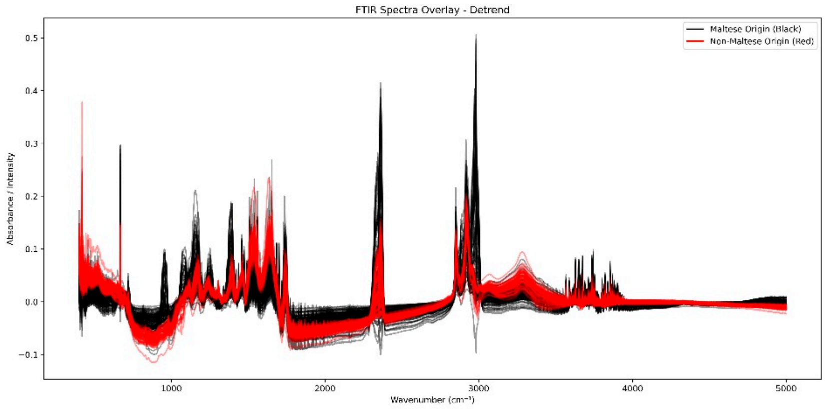
FTIR Spectra Overlay - Detrend
Maltese Origin (Black)
Non-Maltese Origin (Red)
Absorbance / Intensity
Wavenumber (cm⁻¹)

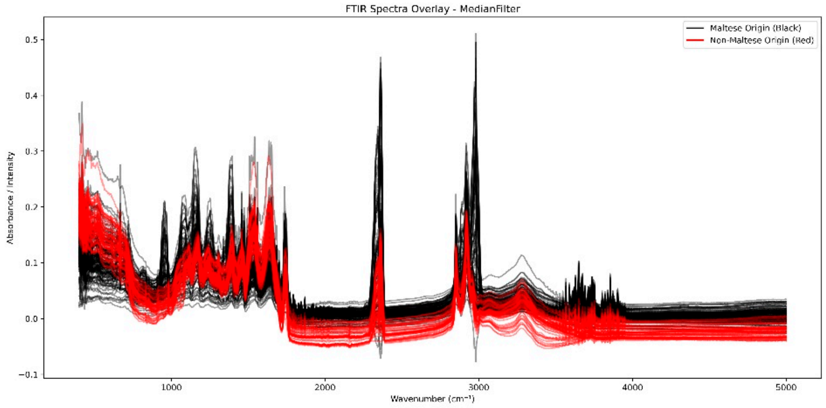
FTIR Spectra Overlay - MedianFilter
Maltese Origin (Black)
Non-Maltese Origin (Red)
Absorbance / Intensity
Wavenumber (cm⁻¹)

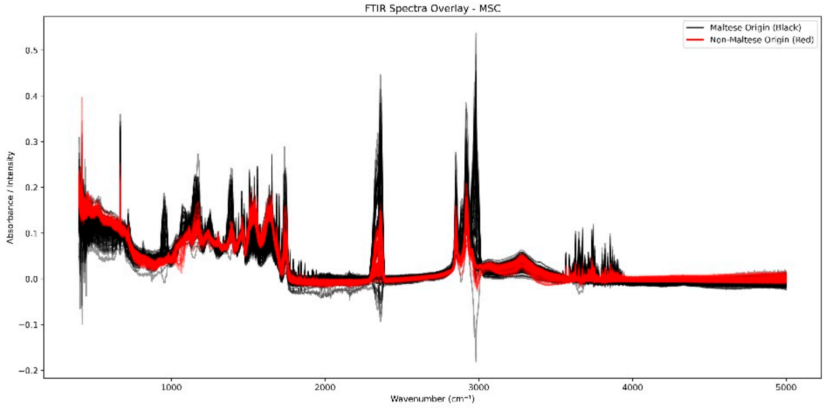
FTIR Spectra Overlay - MSC
Maltese Origin (Black)
Non-Maltese Origin (Red)
Absorbance / Intensity
Wavenumber (cm⁻¹)

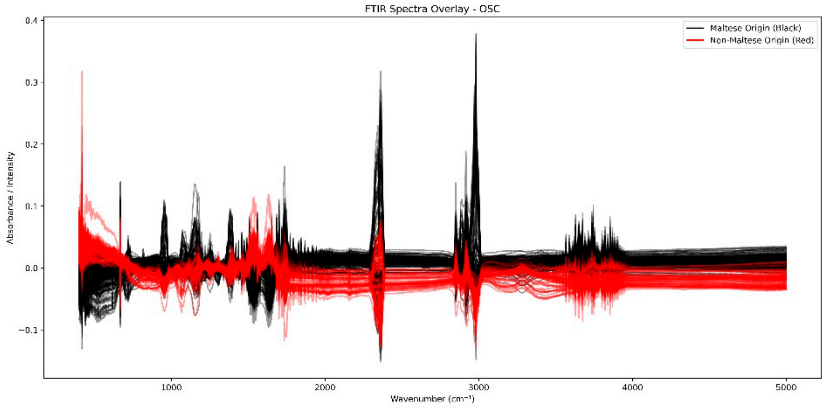
FTIR Spectra Overlay - OSC
Maltese Origin (Black)
Non-Maltese Origin (Red)
Absorbance / Intensity
Wavenumber (cm⁻¹)

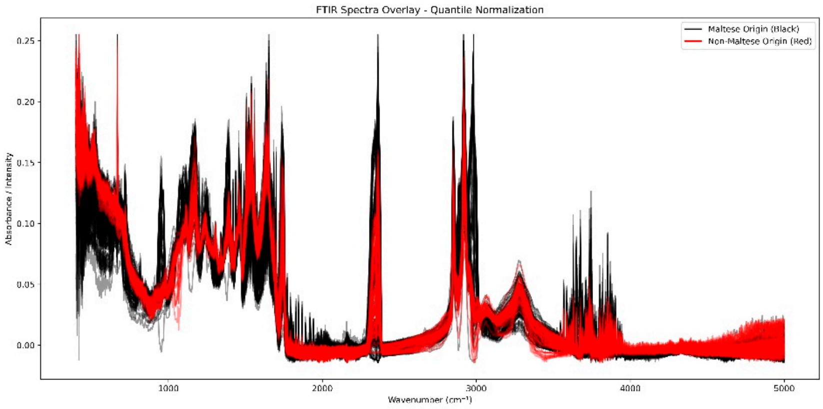
FTIR Spectra Overlay - Quantile Normalization
Maltese Origin (Black)
Non-Maltese Origin (Red)
Absorbance / Intensity
Wavenumber (cm⁻¹)

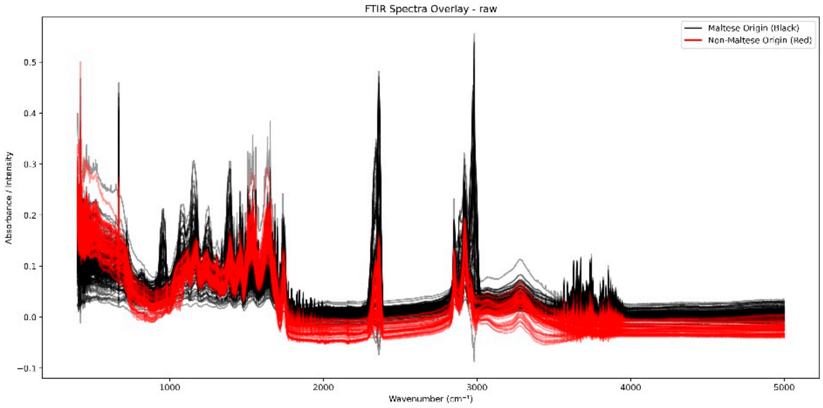
FTIR Spectra Overlay - raw
Maltese Origin (Black)
Non-Maltese Origin (Red)
Absorbance / Intensity
Wavenumber (cm⁻¹)

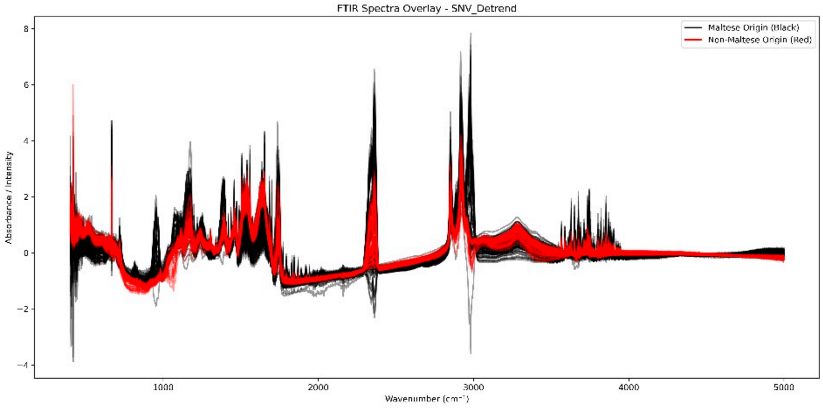
FTIR Spectra Overlay - SNV_Detrend
Maltese Origin (Black)
Non-Maltese Origin (Red)
Absorbance / Intensity
Wavenumber (cm⁻¹)

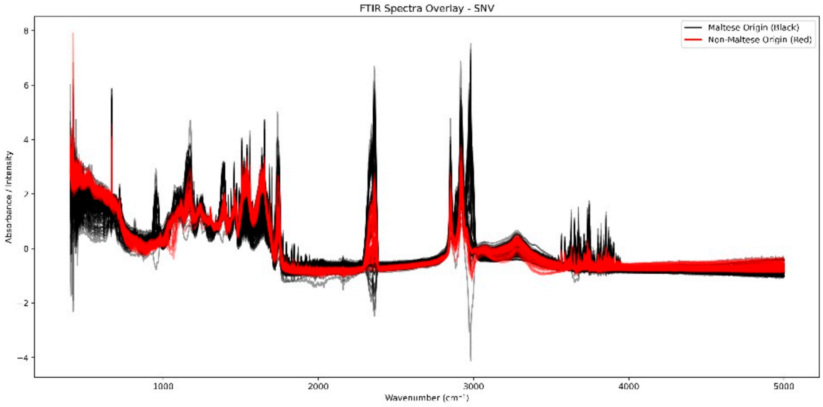
FTIR Spectra Overlay - SNV
Maltese Origin (Black)
Non-Maltese Origin (Red)
Absorbance / Intensity
Wavenumber (cm⁻¹)

Figure S2: PCA score plots of the full spectrum (4000–400 cm^{-1}) for all spectral transformations. Rows correspond to preprocessing methods: 1st Derivative, 2nd Derivative, Deresolve, Detrend, Median Filter, Multiplicative Scatter Correction (MSC), Orthogonal Signal Correction (OSC), Quantile Normalization, Raw, Standard Normal Variate (SNV), and SNV+Detrend

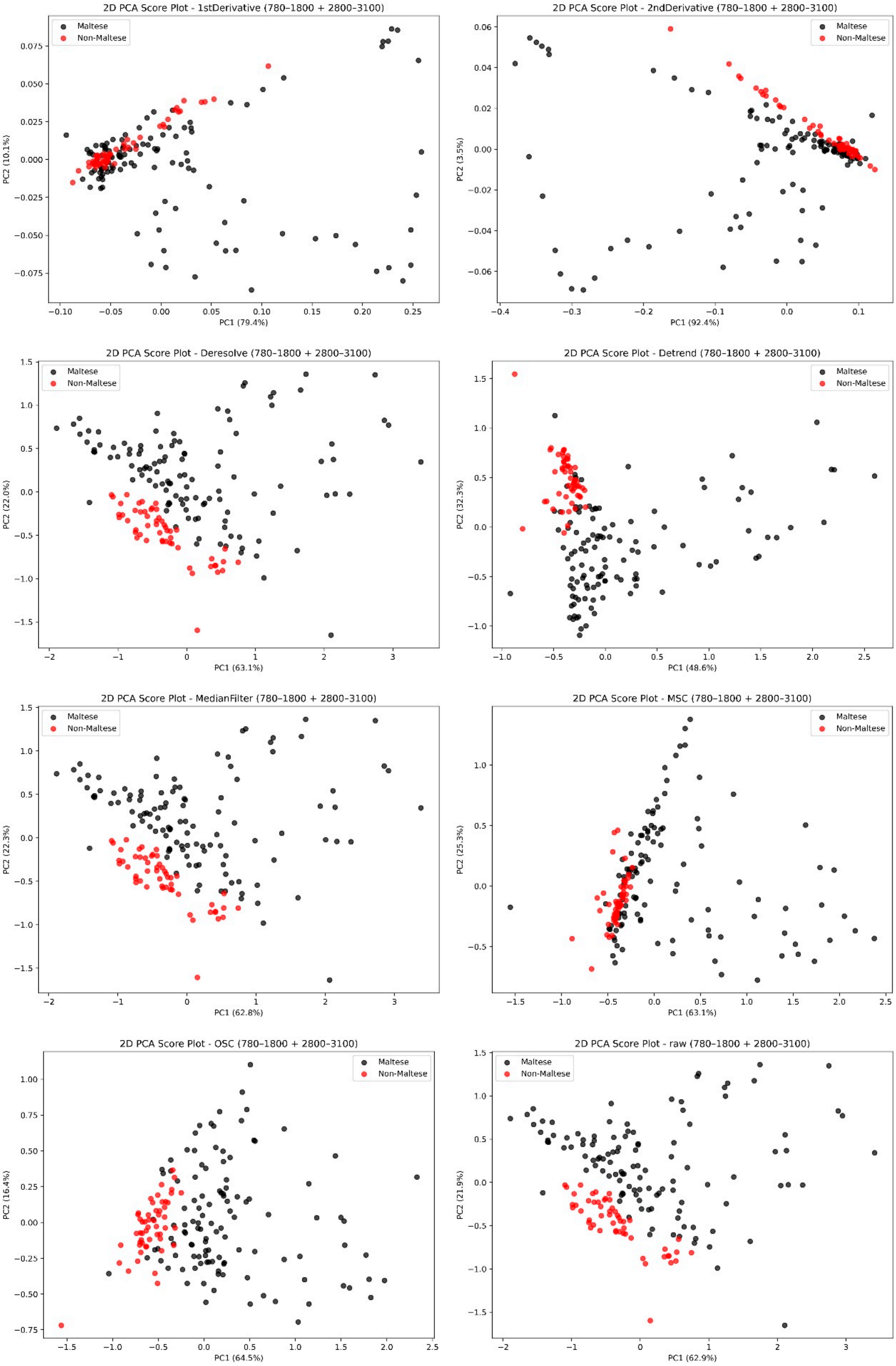

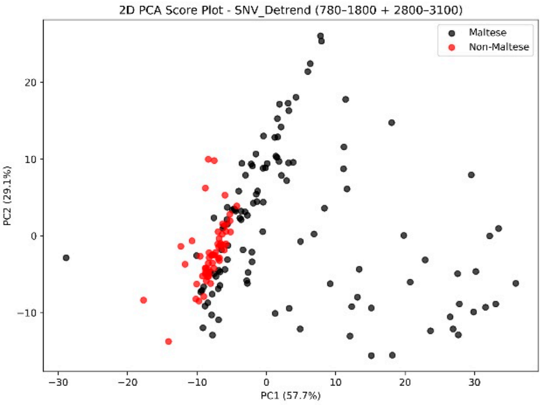
2D PCA Score Plot - SNV_Detrend (780–1800 + 2800–3100)
Maltese
Non-Maltese
PC2 (29.1%)
PC1 (57.7%)
20
10
0
−10
−30
−20
−10
0
10
20
30

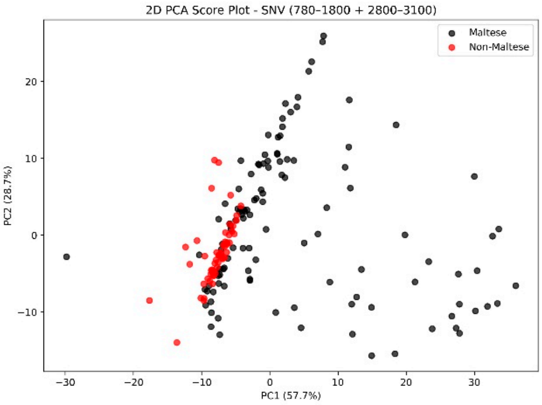
2D PCA Score Plot - SNV (780–1800 + 2800–3100)
Maltese
Non-Maltese
PC2 (28.7%)
PC1 (57.7%)
20
10
0
−10
−30
−20
−10
0
10
20
30

Figure S3: PCA loading plots of the full spectrum (4000–400 cm^{-1}) for all applied spectral transformations. Rows correspond to preprocessing methods: 1st Derivative, 2nd Derivative, Deresolve, Detrend, Median Filter, Multiplicative Scatter Correction (MSC), Orthogonal Signal Correction (OSC), Quantile Normalization, Raw, Standard Normal Variate (SNV), and SNV+Detrend.

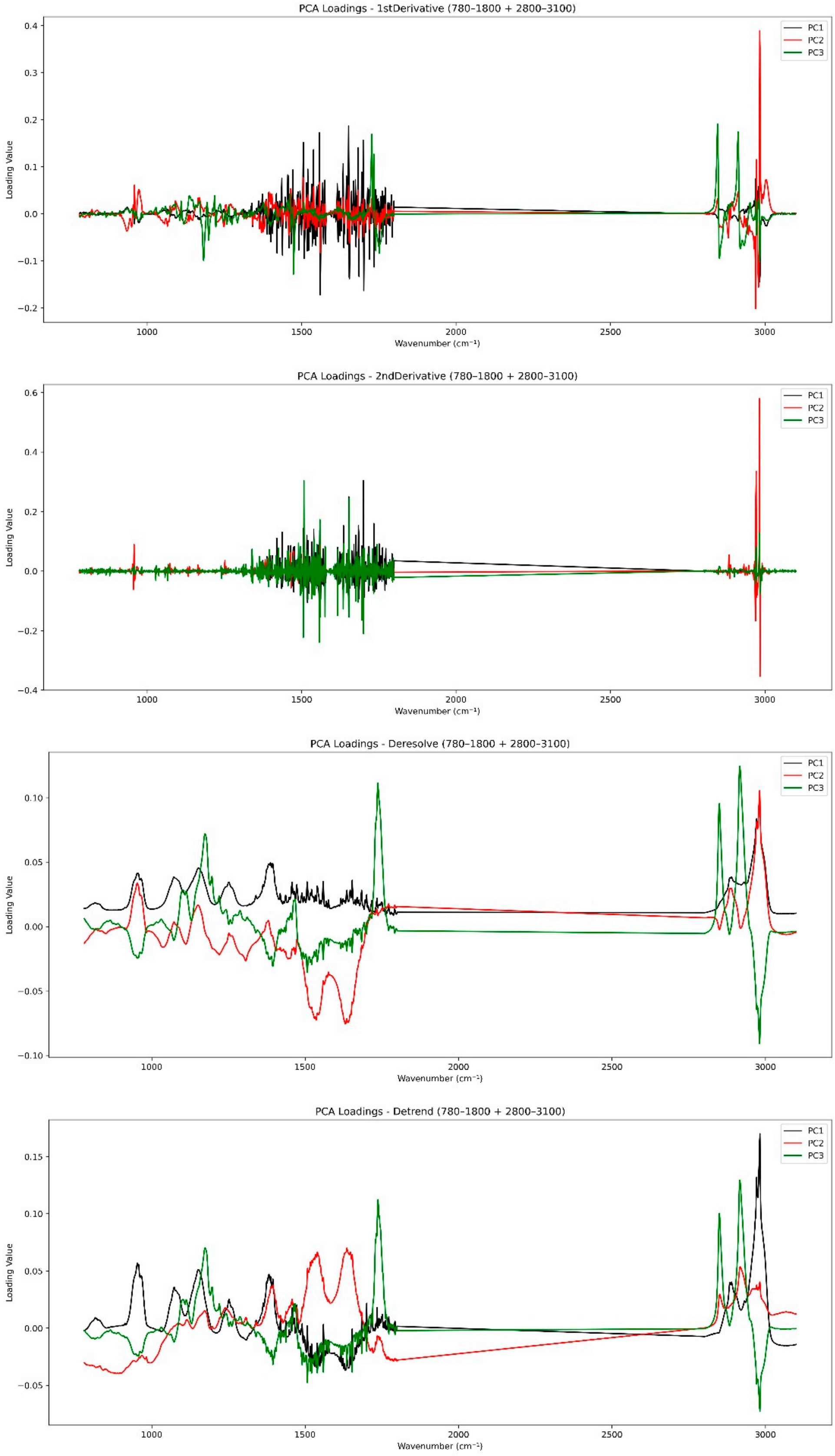

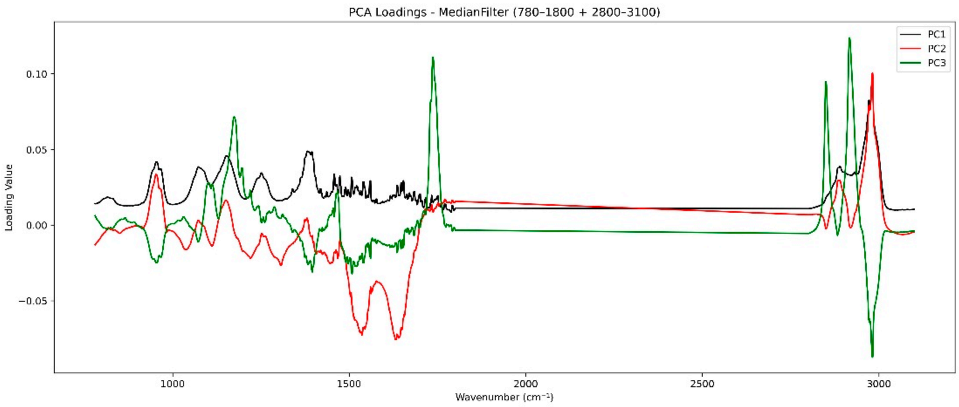
PCA Loadings - MedianFilter (780–1800 + 2800–3100)
PC1
PC2
PC3
Loading Value
Wavenumber (cm⁻¹)

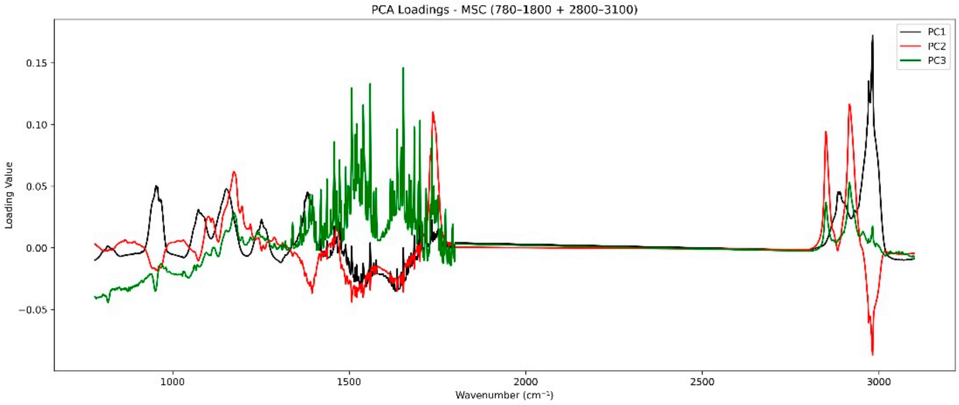
PCA Loadings - MSC (780–1800 + 2800–3100)
PC1
PC2
PC3
Loading Value
Wavenumber (cm⁻¹)

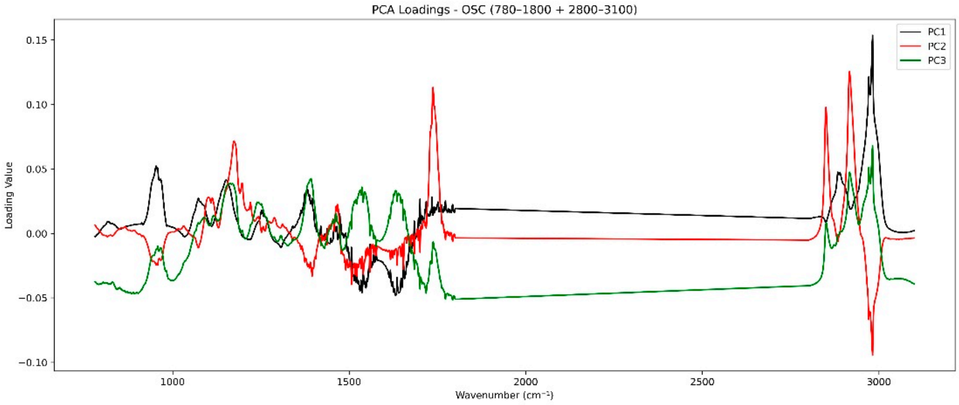
PCA Loadings - OSC (780–1800 + 2800–3100)
PC1
PC2
PC3
Loading Value
Wavenumber (cm⁻¹)

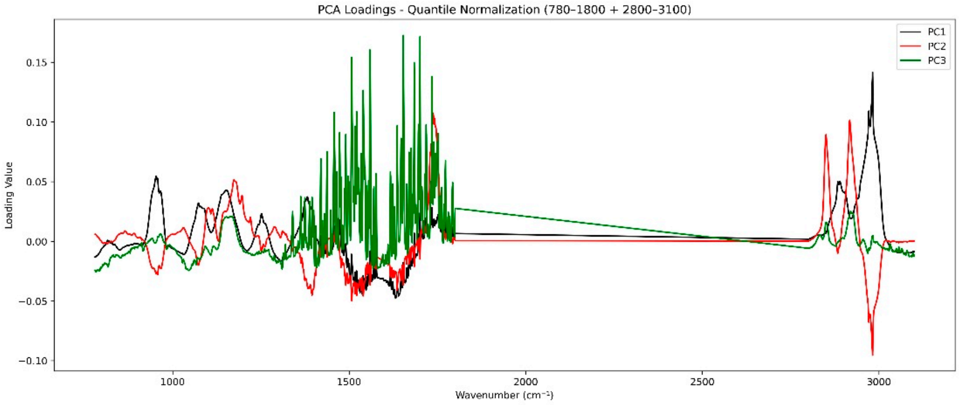
PCA Loadings - Quantile Normalization (780–1800 + 2800–3100)
PC1
PC2
PC3
Loading Value
Wavenumber (cm⁻¹)

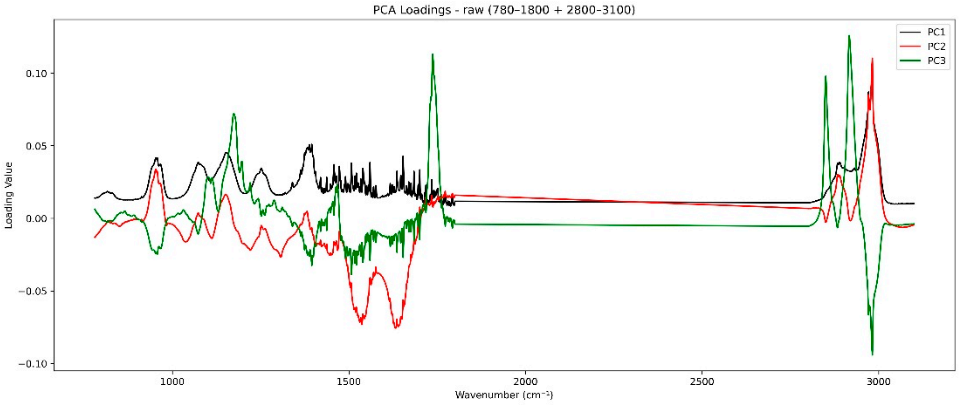
PCA Loadings - raw (780–1800 + 2800–3100)
PC1
PC2
PC3
Loading Value
Wavenumber (cm⁻¹)

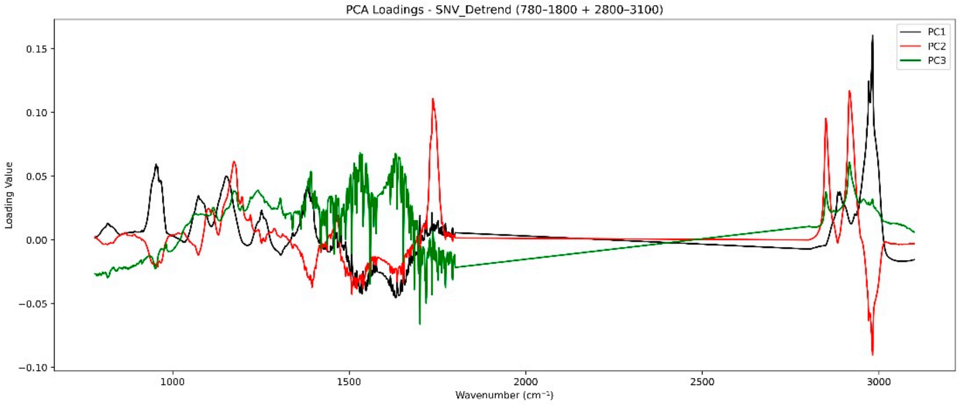
PCA Loadings - SNV_Detrend (780–1800 + 2800–3100)
PC1
PC2
PC3
Loading Value
Wavenumber (cm⁻¹)

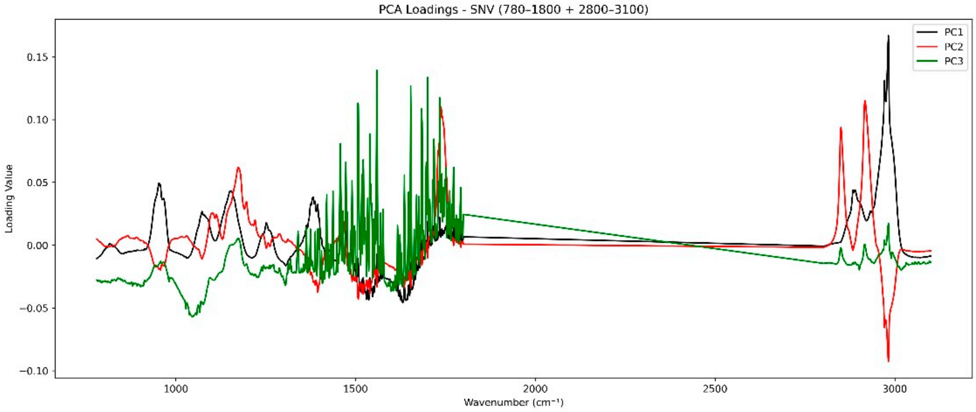
PCA Loadings - SNV (780–1800 + 2800–3100)
PC1
PC2
PC3
Loading Value
Wavenumber (cm⁻¹)

Figure S4: PCA score plots of the fingerprint region (1800–850 cm^{-1}) for all spectral transformations. Rows correspond to the different preprocessing methods applied: 1st Derivative, 2nd Derivative, Deresolve, Detrend, Median Filter, Multiplicative Scatter Correction (MSC), Orthogonal Signal Correction (OSC), Quantile Normalization, Raw, Standard Normal Variate (SNV), and SNV+Detrend

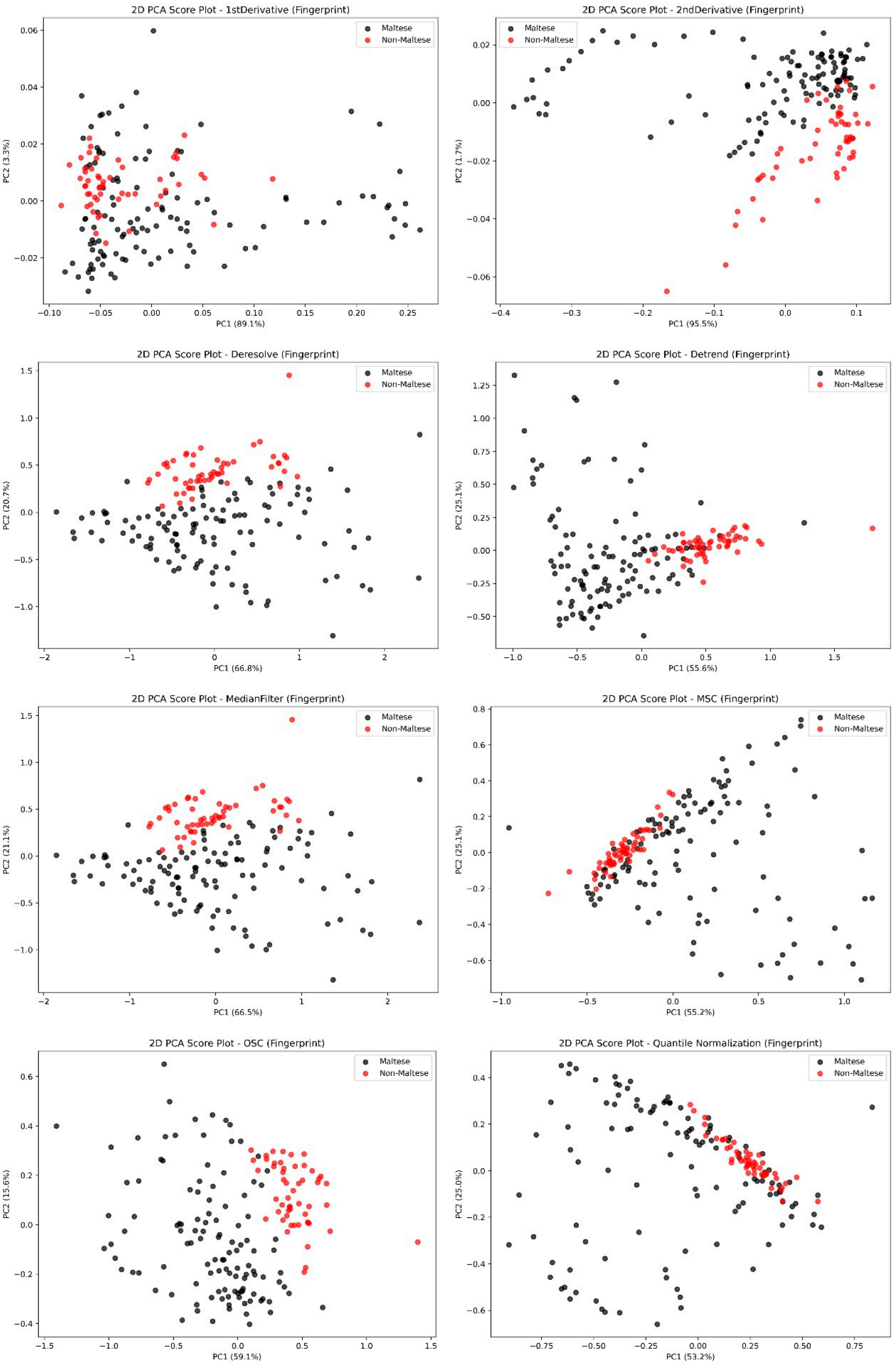

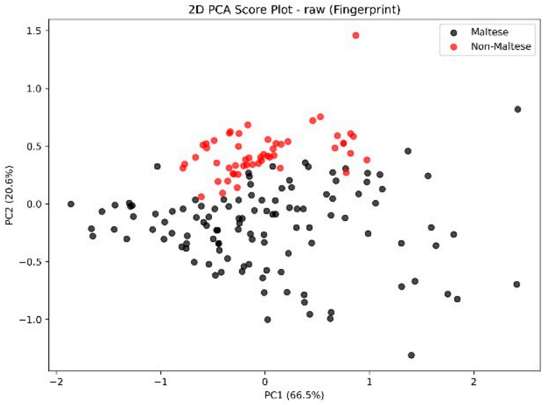
2D PCA Score Plot - raw (Fingerprint)
Maltese
Non-Maltese
PC1 (66.5%)
PC2 (20.6%)

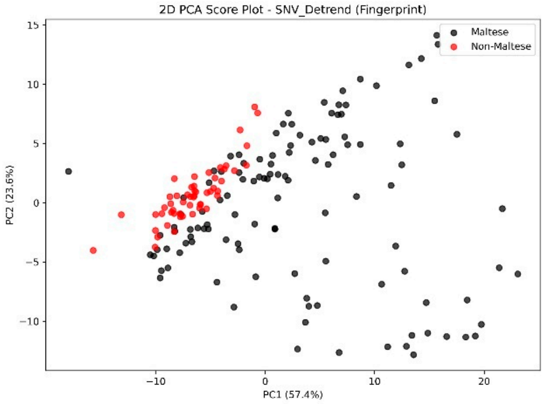
2D PCA Score Plot - SNV_Detrend (Fingerprint)
Maltese
Non-Maltese
PC1 (57.4%)
PC2 (23.6%)

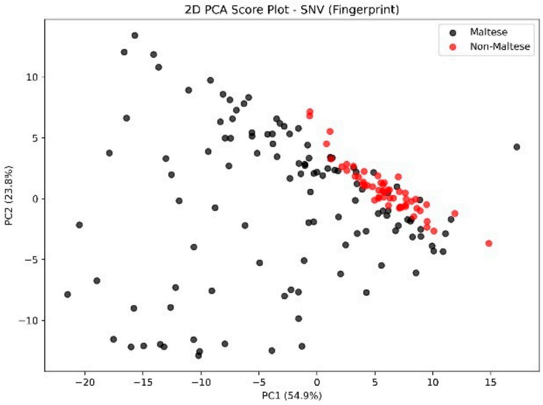
2D PCA Score Plot - SNV (Fingerprint)
Maltese
Non-Maltese
PC1 (54.9%)
PC2 (23.8%)

Figure S5: PCA loading plots of the fingerprint region (1800–850 cm^{-1}) for all applied spectral transformations. Rows correspond to preprocessing methods: 1st Derivative, 2nd Derivative, Deresolve, Detrend, Median Filter, Multiplicative Scatter Correction (MSC), Orthogonal Signal Correction (OSC), Quantile Normalization, Raw, Standard Normal Variate (SNV), and SNV+Detrend

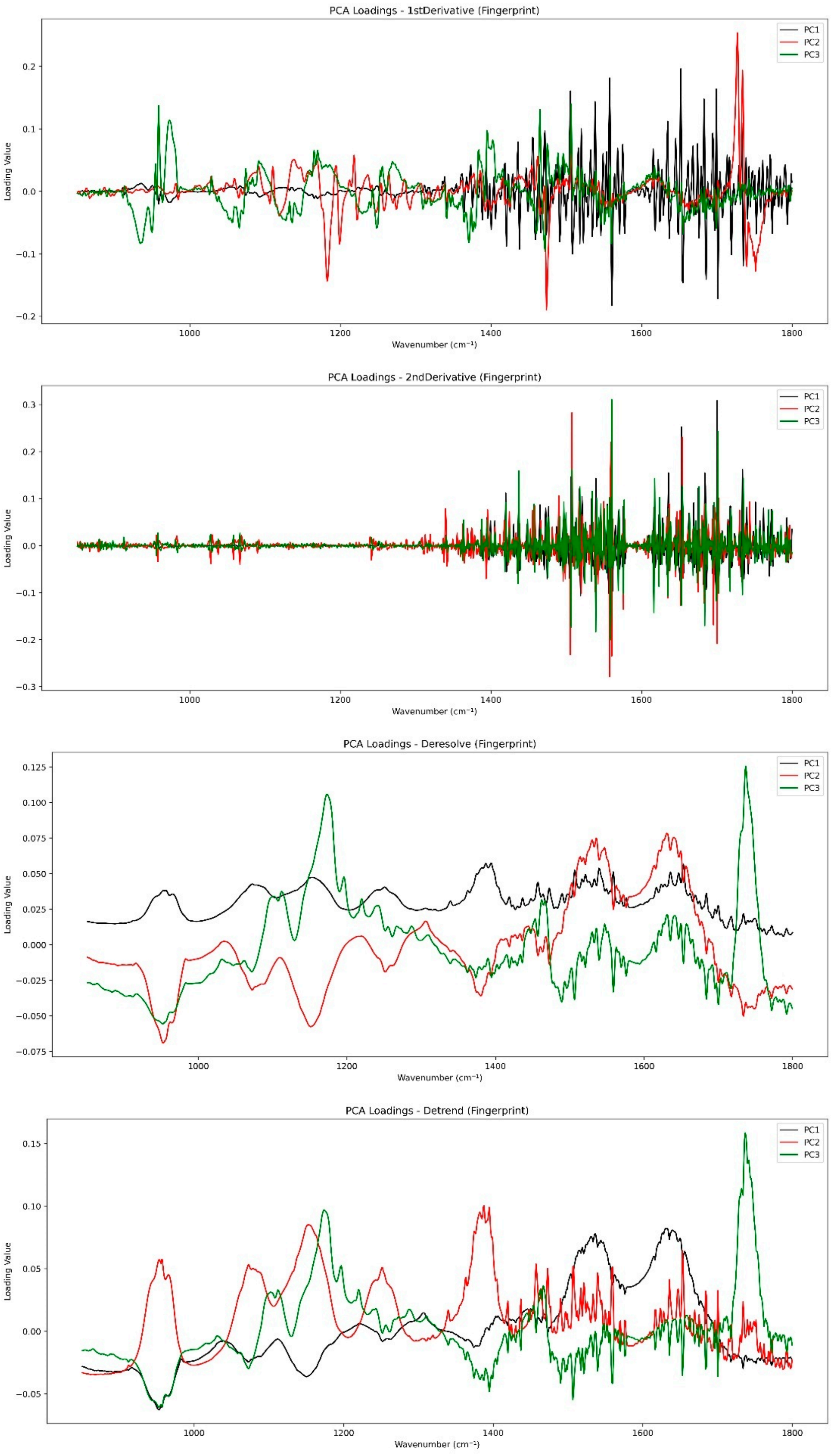

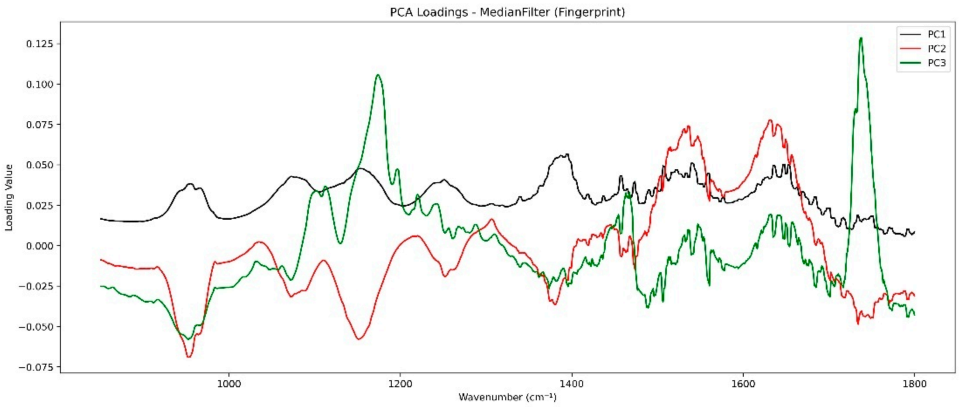
PCA Loadings - MedianFilter (Fingerprint)
Loading Value
Wavenumber (cm⁻¹)
PC1
PC2
PC3
0.125
0.100
0.075
0.050
0.025
0.000
−0.025
−0.050
−0.075
1000
1200
1400
1600
1800

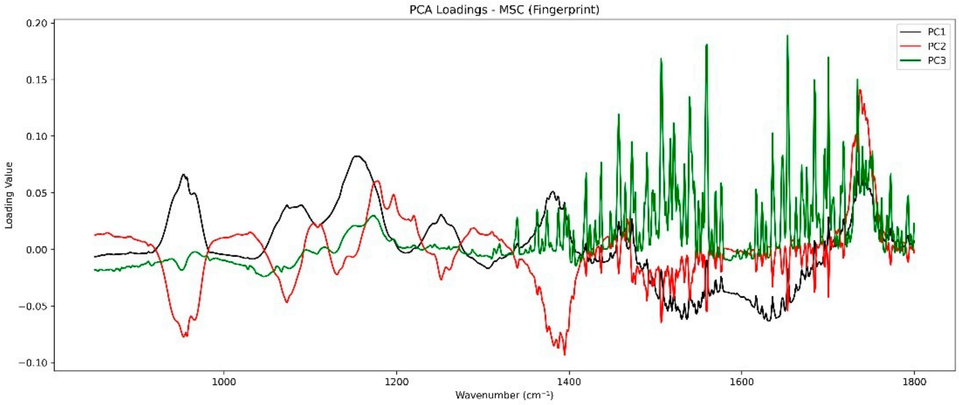
PCA Loadings - MSC (Fingerprint)
Loading Value
Wavenumber (cm⁻¹)
PC1
PC2
PC3
0.20
0.15
0.10
0.05
0.00
−0.05
−0.10
1000
1200
1400
1600
1800

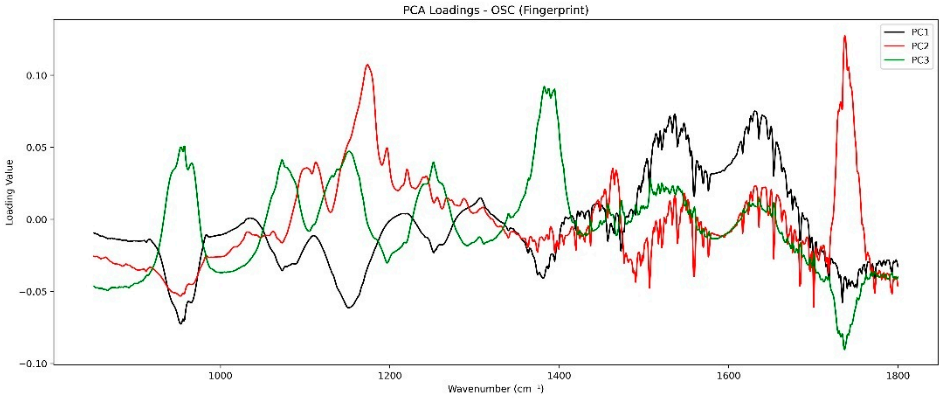
PCA Loadings - OSC (Fingerprint)
Loading Value
Wavenumber (cm⁻¹)
PC1
PC2
PC3
0.10
0.05
0.00
−0.05
−0.10
1000
1200
1400
1600
1800

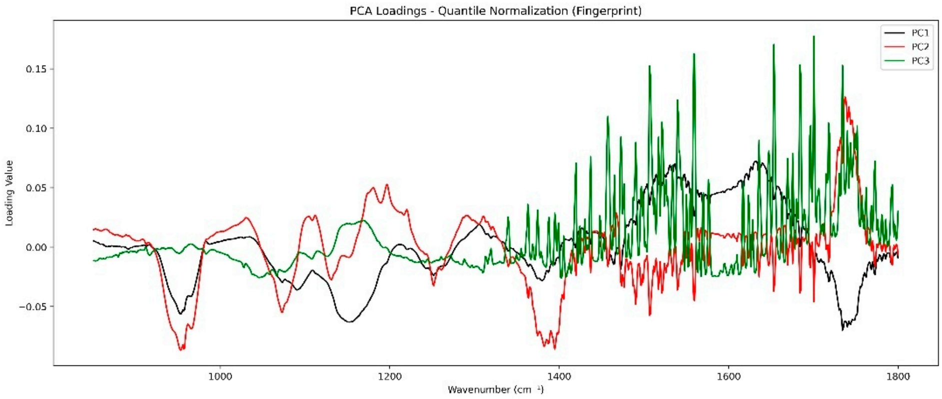
PCA Loadings - Quantile Normalization (Fingerprint)
Loading Value
Wavenumber (cm⁻¹)
PC1
PC2
PC3
0.15
0.10
0.05
0.00
−0.05
1000
1200
1400
1600
1800

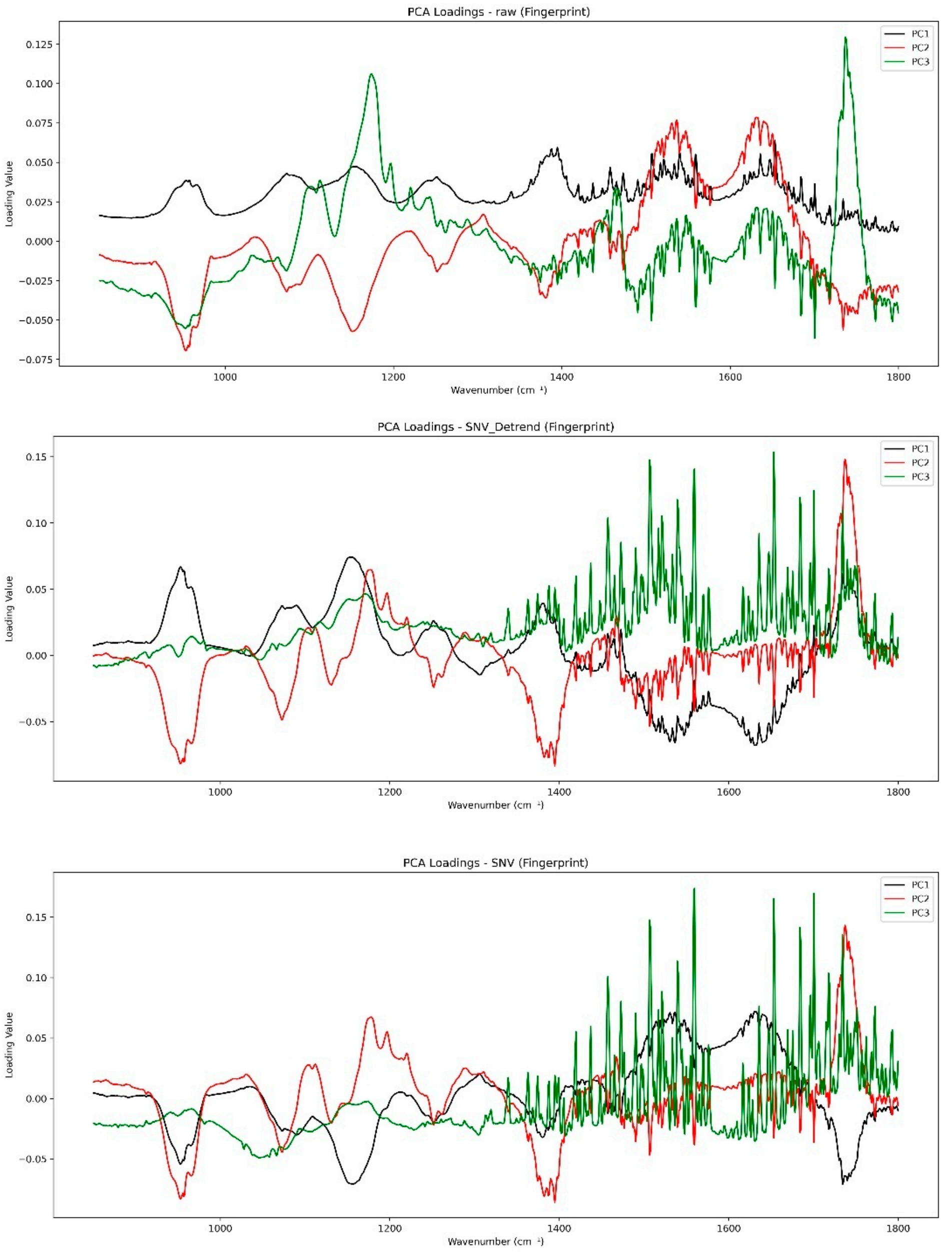

PCA Loadings - raw (Fingerprint)
Loading Value
Wavenumber (cm⁻¹)
PC1
PC2
PC3
0.125
0.100
0.075
0.050
0.025
0.000
−0.025
−0.050
−0.075
1000
1200
1400
1600
1800
PCA Loadings - SNV_Detrend (Fingerprint)
Loading Value
Wavenumber (cm⁻¹)
PC1
PC2
PC3
0.15
0.10
0.05
0.00
−0.05
1000
1200
1400
1600
1800
PCA Loadings - SNV (Fingerprint)
Loading Value
Wavenumber (cm⁻¹)
PC1
PC2
PC3
0.15
0.10
0.05
0.00
−0.05
1000
1200
1400
1600
1800

Figure S6: . Outlier analysis using Hotelling's T^2 plots for all spectral transformations comparing the whole spectrum (right panels) and the fingerprint region (left panels). Rows correspond to different preprocessing methods applied to the ATR-FTIR pork dataset: 1st Derivative, 2nd Derivative, Deresolve, Detrend, Median Filter, MSC, OSC, Quantile Normalization, Raw, SNV, and SNV+Detrend.

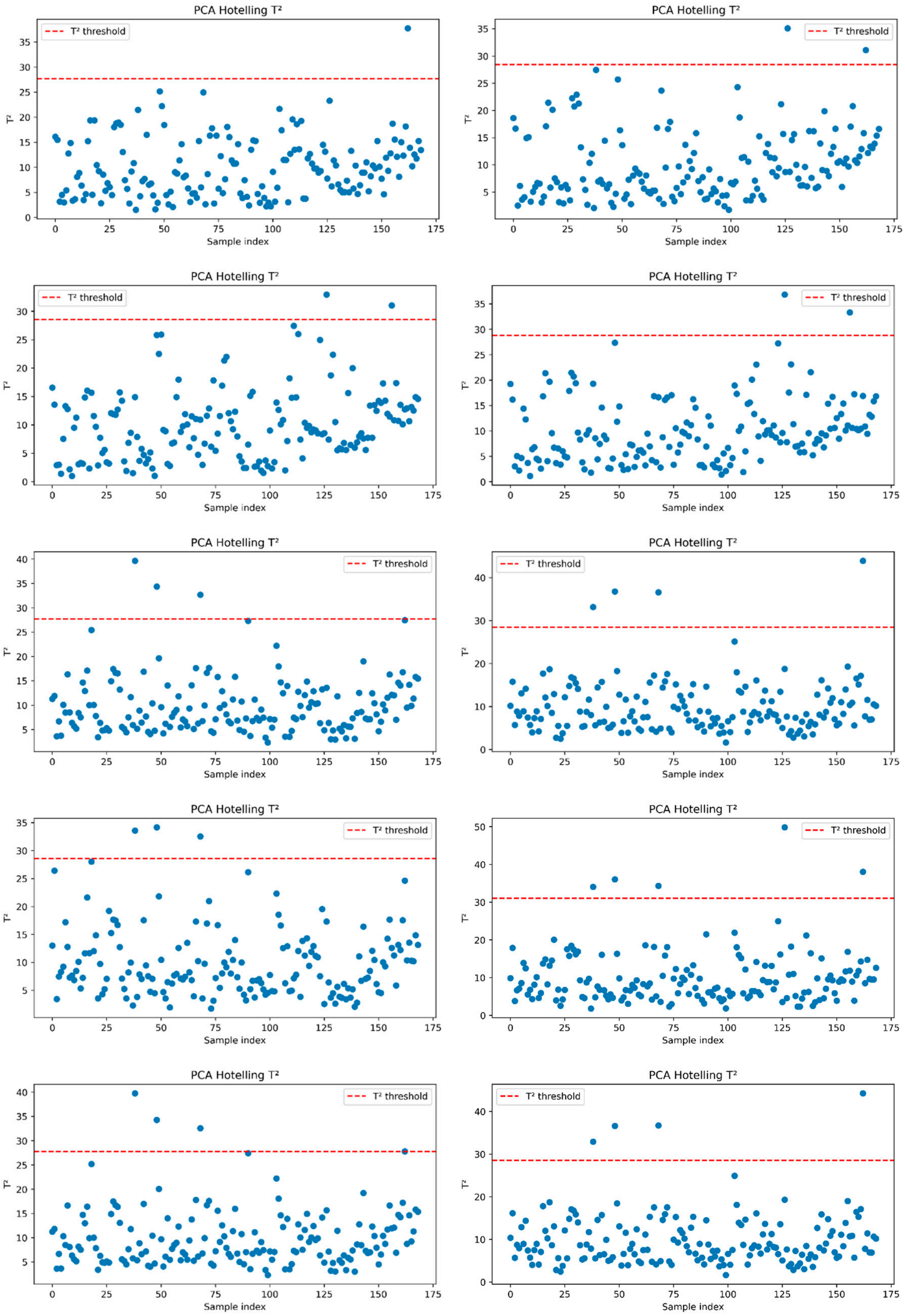

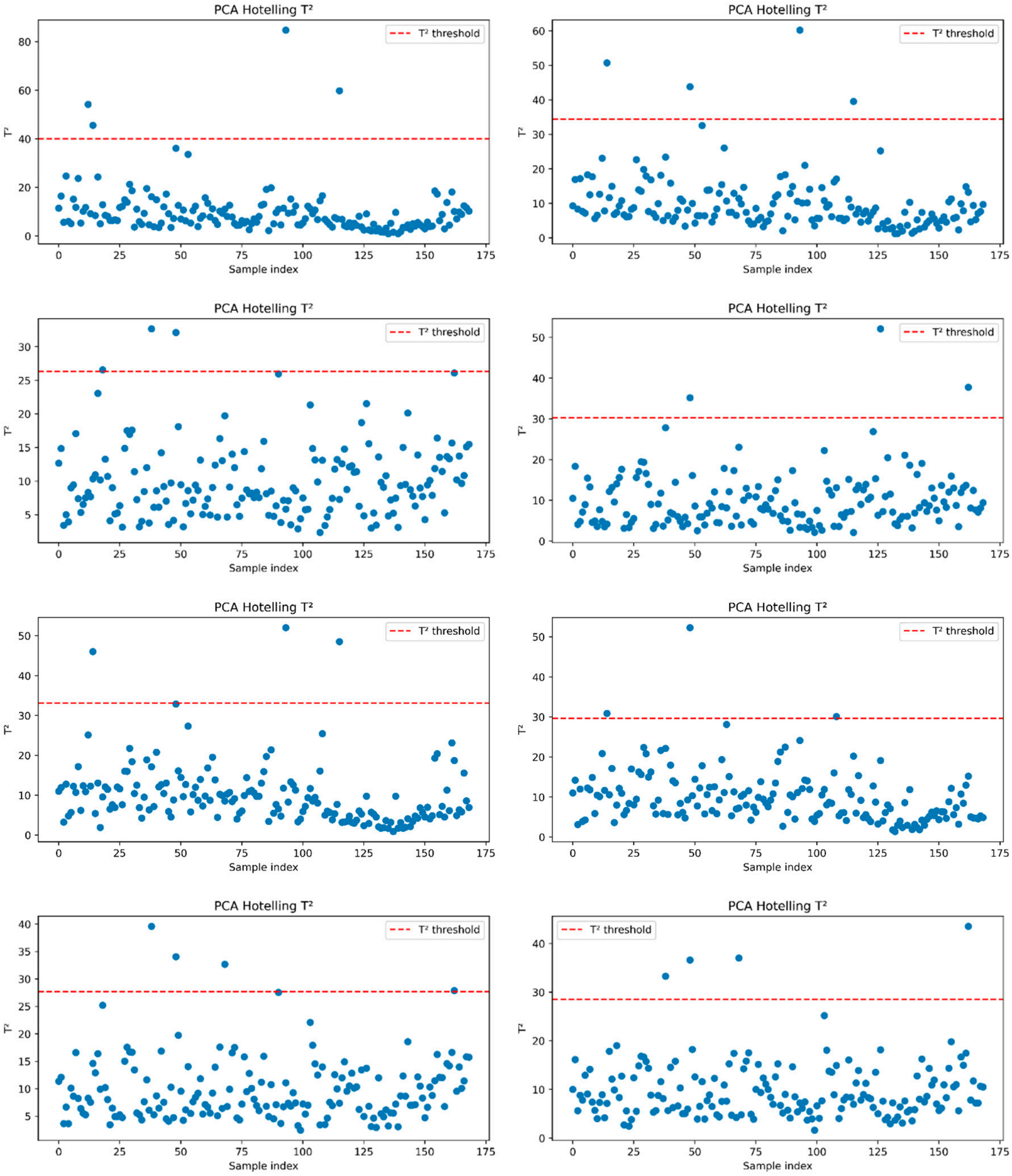
PCA Hotelling T²
T² threshold
T²
Sample index

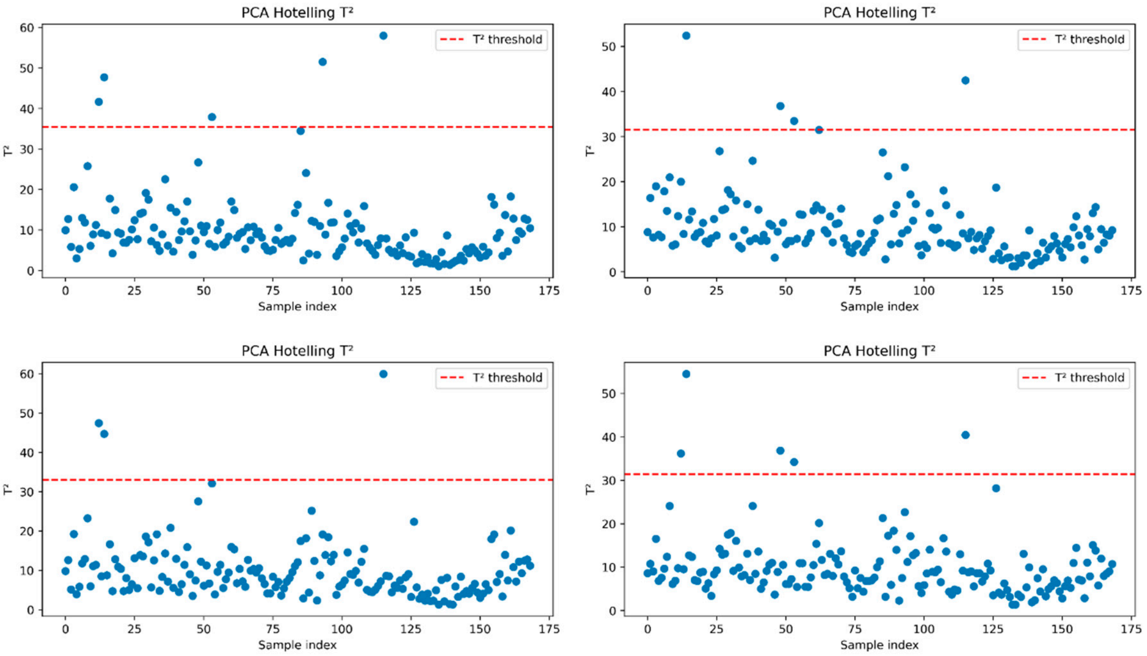
PCA Hotelling T^2
T^2 threshold
T^2
Sample index
PCA Hotelling T^2
T^2 threshold
T^2
Sample index
PCA Hotelling T^2
T^2 threshold
T^2
Sample index
PCA Hotelling T^2
T^2 threshold
T^2
Sample index

Figure S7: . Q resdiual plots for all spectral transformations comparing the whole spectrum (right panels) and the fingerprint region (left panels). Rows correspond to different preprocessing methods applied to the ATR-FTIR pork dataset: 1st Derivative, 2nd Derivative, Deresolve, Detrend, Median Filter, MSC, OSC, Quantile Normalization, Raw, SNV, and SNV+Detrend.

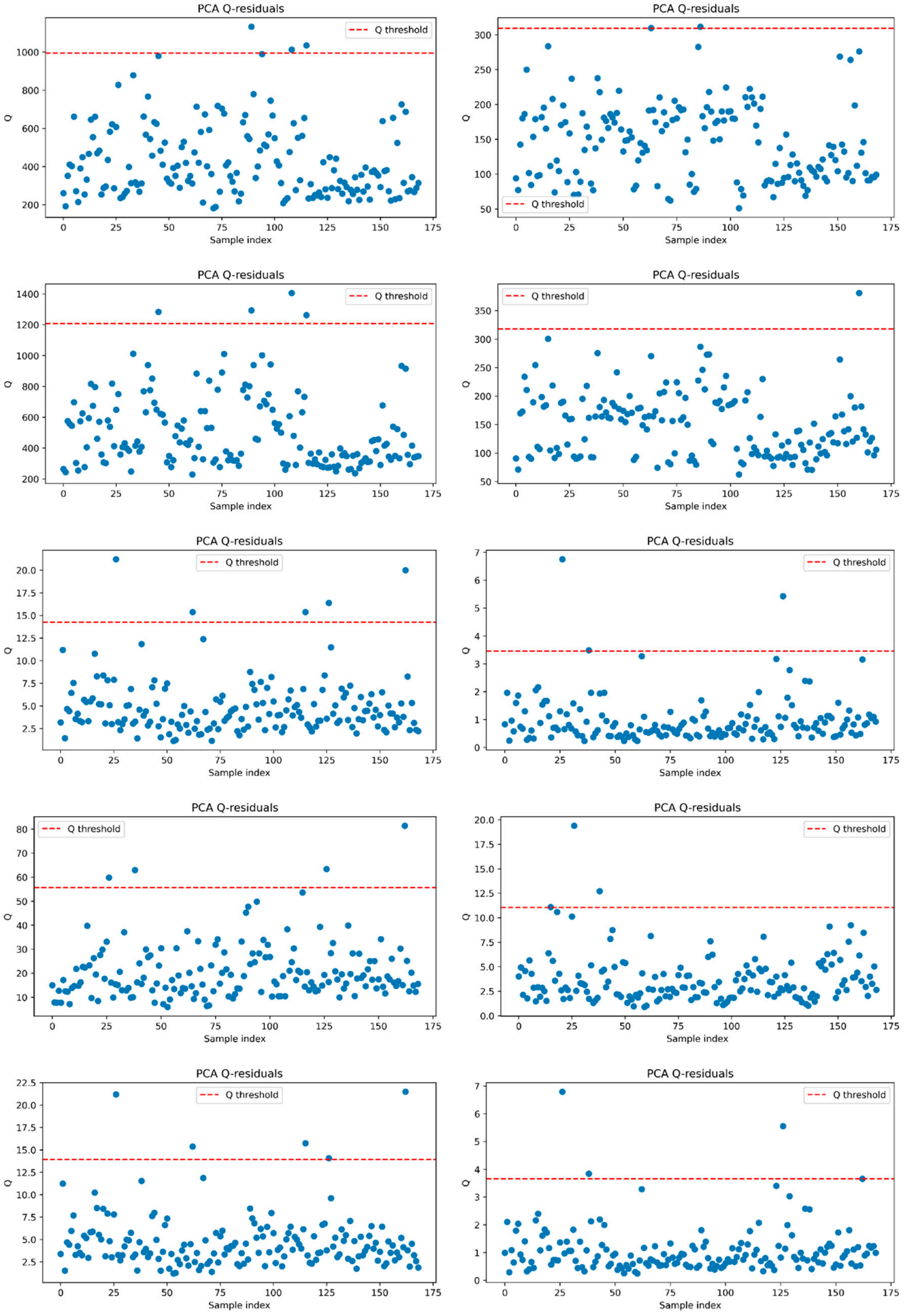

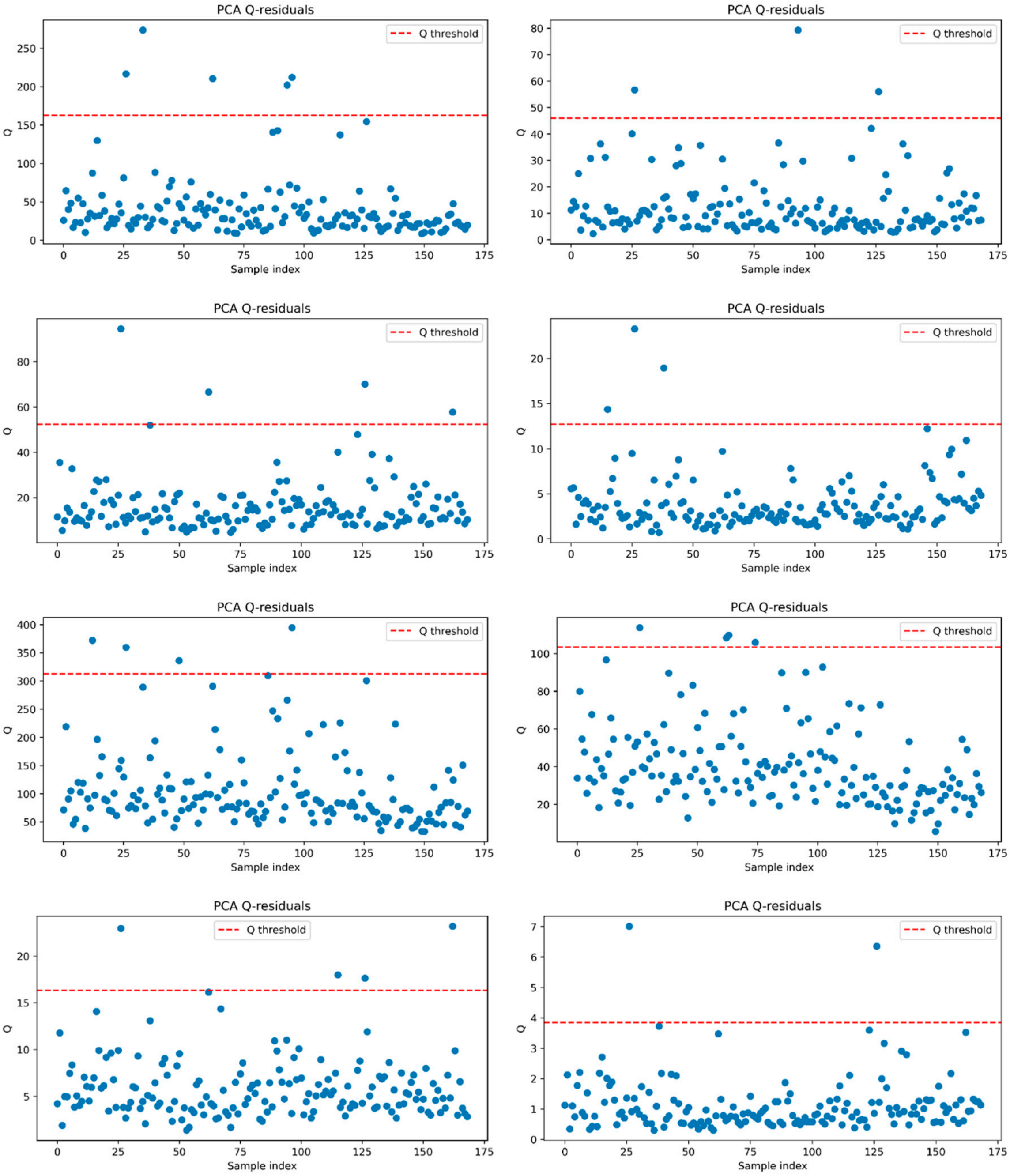
PCA Q-residuals
Q threshold
Q
Sample index
PCA Q-residuals
Q threshold
Q
Sample index
PCA Q-residuals
Q threshold
Q
Sample index
PCA Q-residuals
Q threshold
Q
Sample index
PCA Q-residuals
Q threshold
Q
Sample index
PCA Q-residuals
Q threshold
Q
Sample index
PCA Q-residuals
Q threshold
Q
Sample index
PCA Q-residuals
Q threshold
Q
Sample index

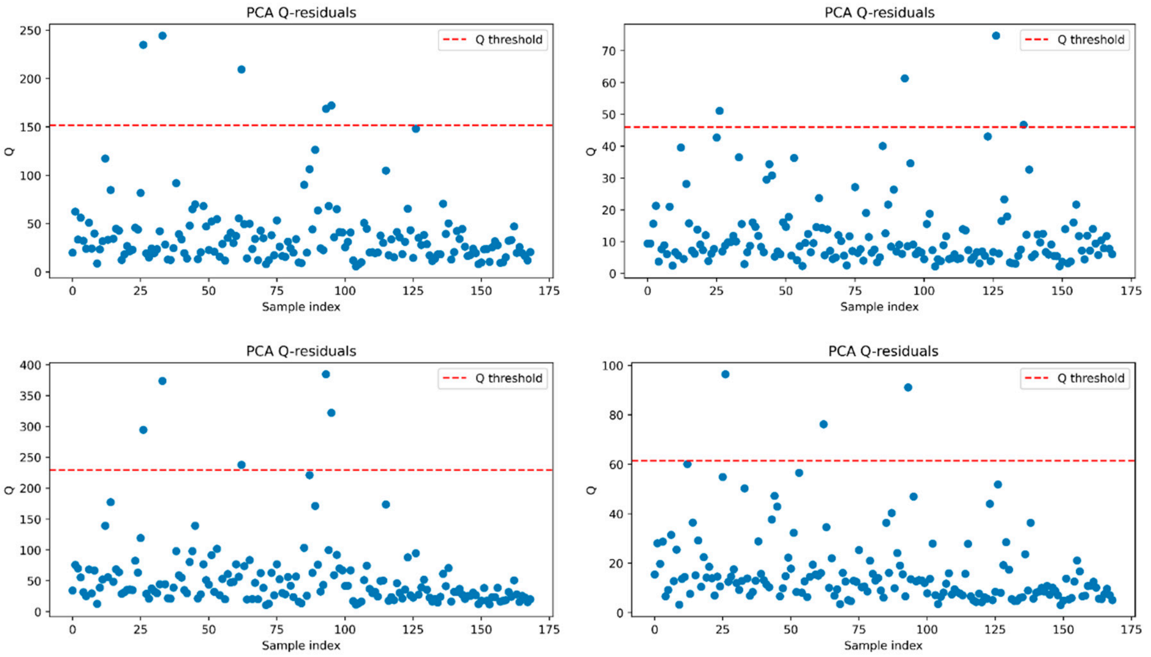
PCA Q-residuals
Q threshold
Q
Sample index
PCA Q-residuals
Q threshold
Q
Sample index
PCA Q-residuals
Q threshold
Q
Sample index
PCA Q-residuals
Q threshold
Q
Sample index

Figure S8: . Coomans plots (Q-residuals vs Hotelling's T^2) for all spectral transformations, comparing the *whole spectrum* (right panels) and the *fingerprint region* (left panels). Rows correspond to different preprocessing methods applied to the ATR-FTIR pork dataset: 1st Derivative, 2nd Derivative, Deresolve, Detrend, Median Filter, Multiplicative Scatter Correction (MSC), Orthogonal Signal Correction (OSC), Quantile Normalization, Raw, Standard Normal Variate (SNV), and SNV+Detrend.

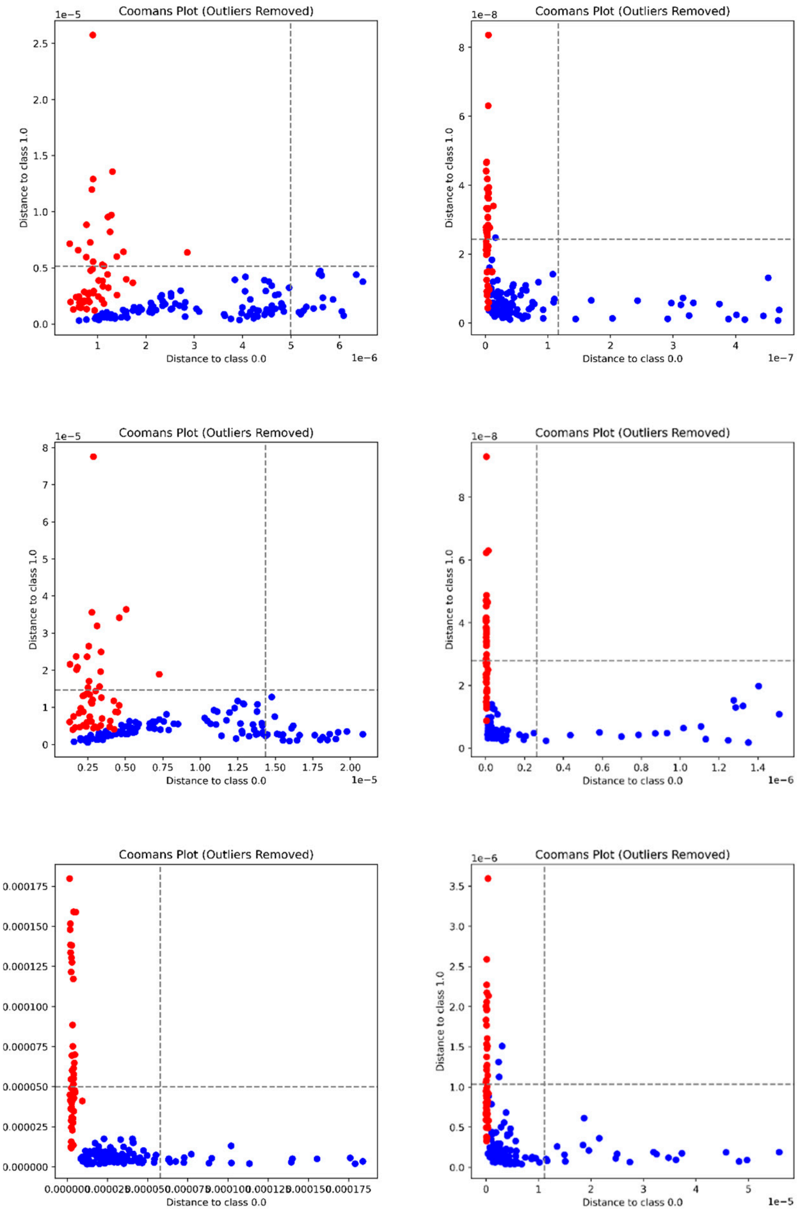

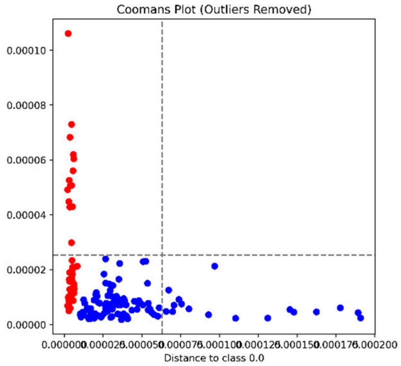
Coomans Plot (Outliers Removed)
Distance to class 0.0
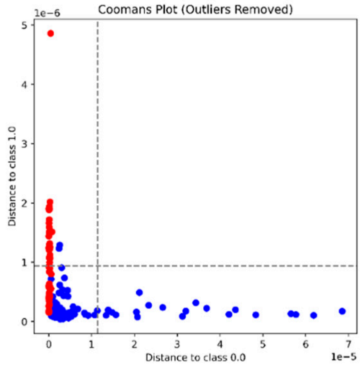
Coomans Plot (Outliers Removed)
Distance to class 1.0
Distance to class 0.0
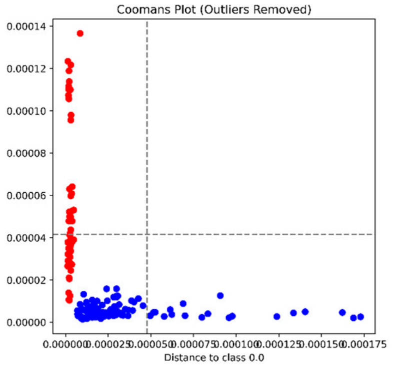
Coomans Plot (Outliers Removed)
Distance to class 0.0
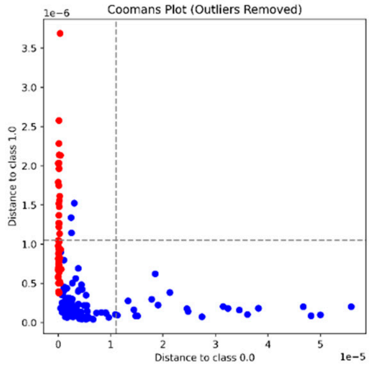
Coomans Plot (Outliers Removed)
Distance to class 1.0
Distance to class 0.0
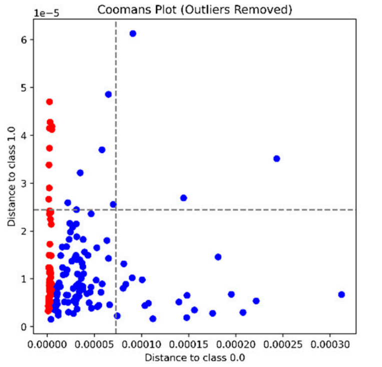
Coomans Plot (Outliers Removed)
Distance to class 1.0
Distance to class 0.0
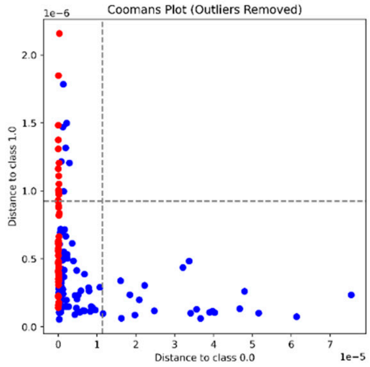
Coomans Plot (Outliers Removed)
Distance to class 1.0
Distance to class 0.0
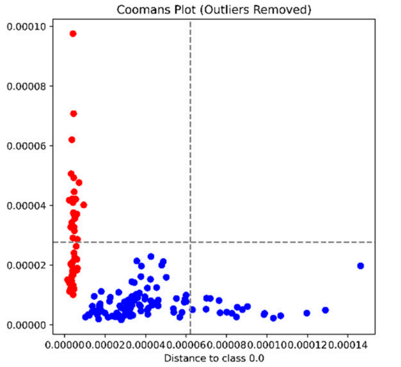
Coomans Plot (Outliers Removed)
Distance to class 0.0
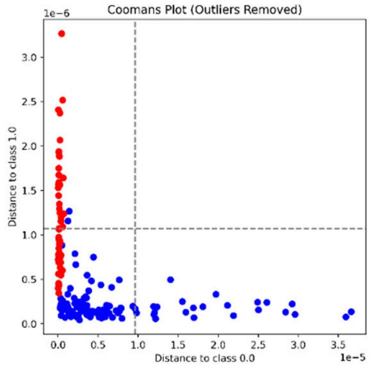
Coomans Plot (Outliers Removed)
Distance to class 1.0
Distance to class 0.0

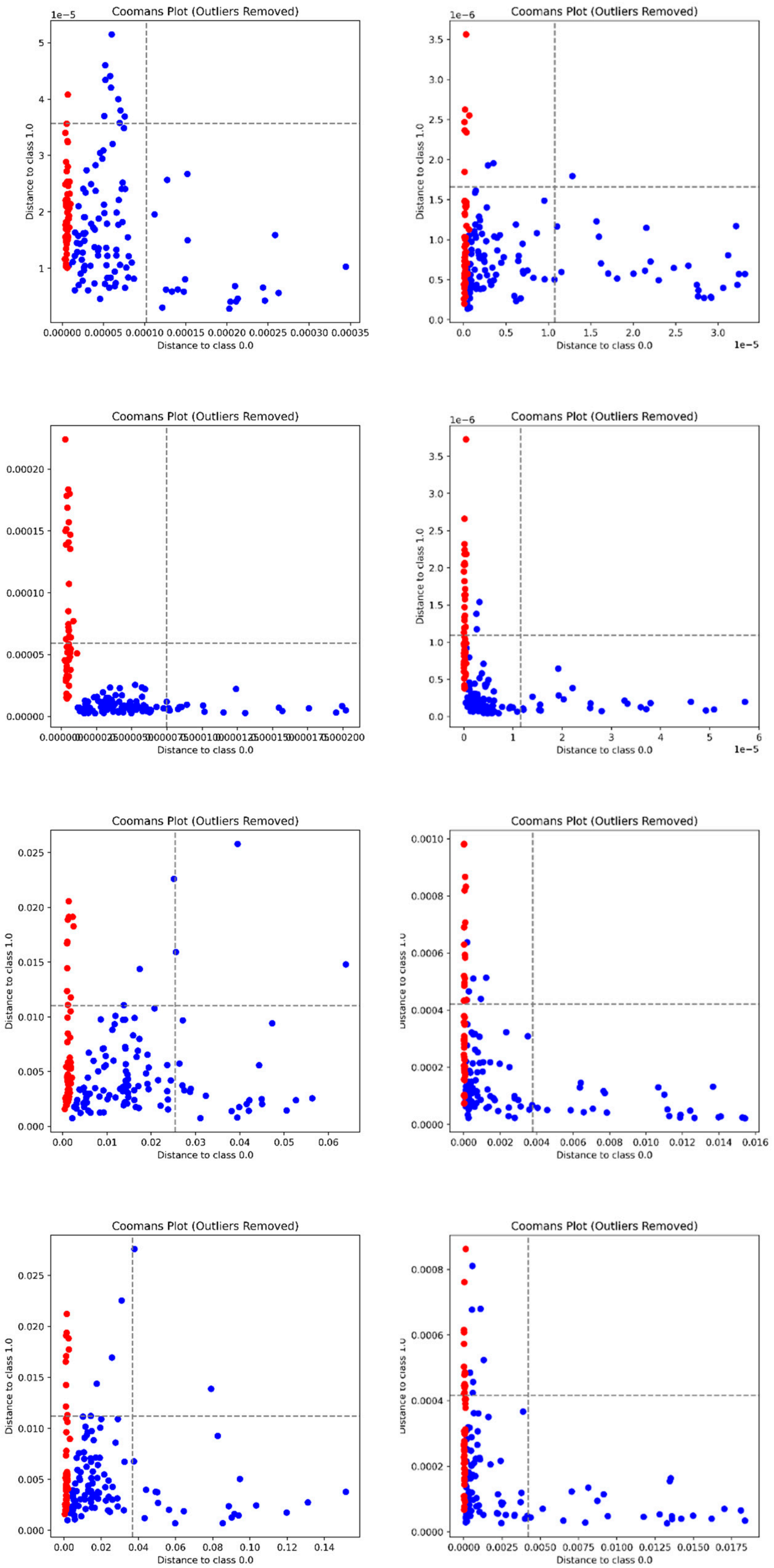

Coomans Plot (Outliers Removed)
Distance to class 1.0
Distance to class 0.0
Coomans Plot (Outliers Removed)
Distance to class 1.0
Distance to class 0.0
Coomans Plot (Outliers Removed)
Distance to class 0.0
Coomans Plot (Outliers Removed)
Distance to class 1.0
Distance to class 0.0
Coomans Plot (Outliers Removed)
Distance to class 1.0
Distance to class 0.0
Coomans Plot (Outliers Removed)
Distance to class 0.0
Coomans Plot (Outliers Removed)
Distance to class 1.0
Distance to class 0.0
Coomans Plot (Outliers Removed)
Distance to class 0.0

Figure S9. SIMCA classification performance metrics (Accuracy and Specificity) comparing the *whole spectrum* and the *fingerprint region* across different spectral transformations: 1st Derivative, 2nd Derivative, Deresolve, Detrend, Multiplicative Scatter Correction (MSC), Median Filter, Orthogonal Signal Correction (OSC), Quantile Normalization, Standard Normal Variate (SNV), SNV+Detrend, and Raw. Bars represent classification accuracy (blue = whole spectrum; orange = fingerprint) and specificity (green = whole spectrum; pink = fingerprint).

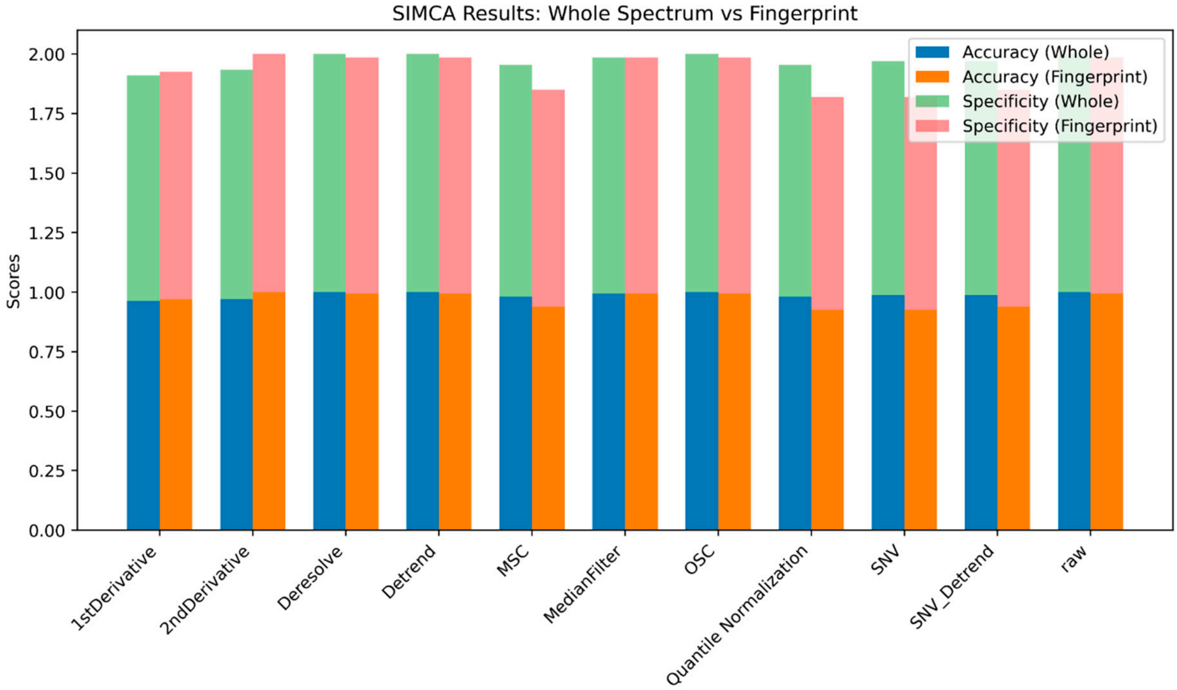

Figure S10: . PLS-DA score plots for all spectral transformations, comparing the *whole spectrum* (right panels) and the *fingerprint region* (left panels). Rows correspond to different preprocessing methods applied to the ATR-FTIR pork dataset: 1st Derivative, 2nd Derivative, Deresolve, Detrend, Median Filter, Multiplicative Scatter Correction (MSC), Orthogonal Signal Correction (OSC), Quantile Normalization, Raw, Standard Normal Variate (SNV), and SNV+Detrend.

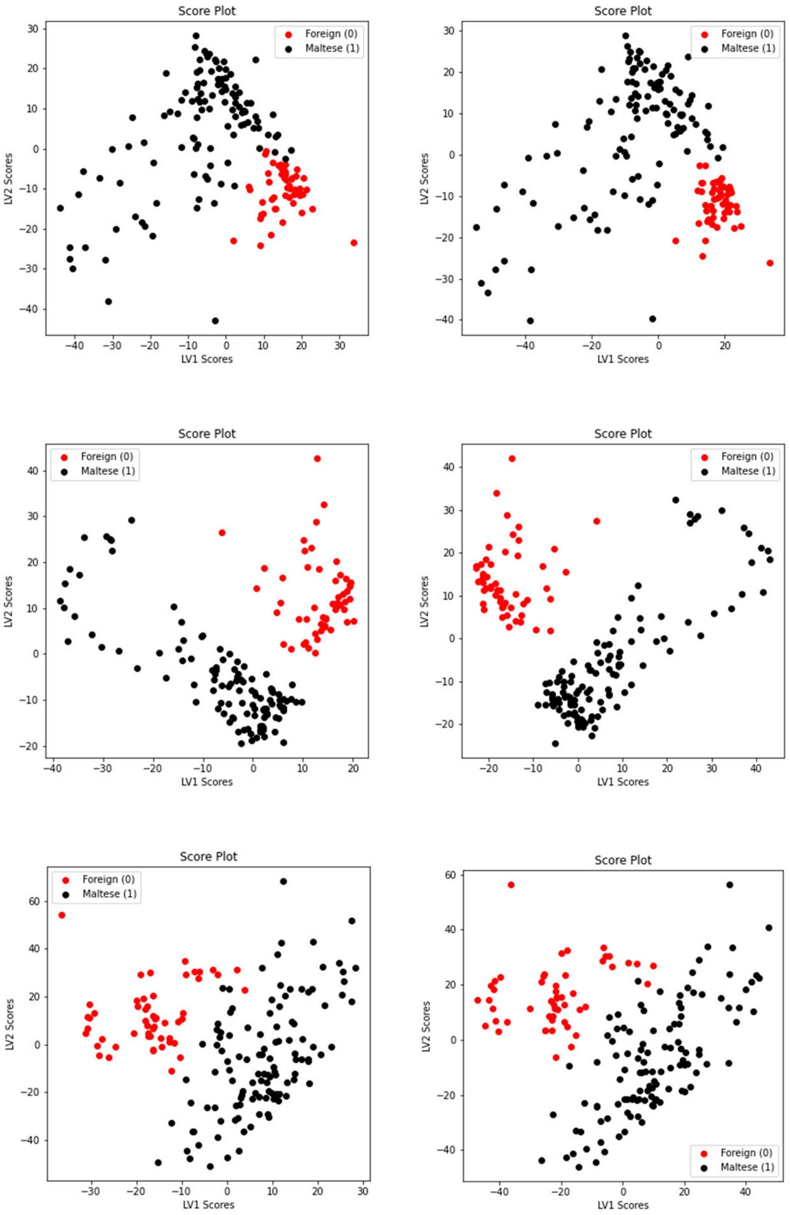

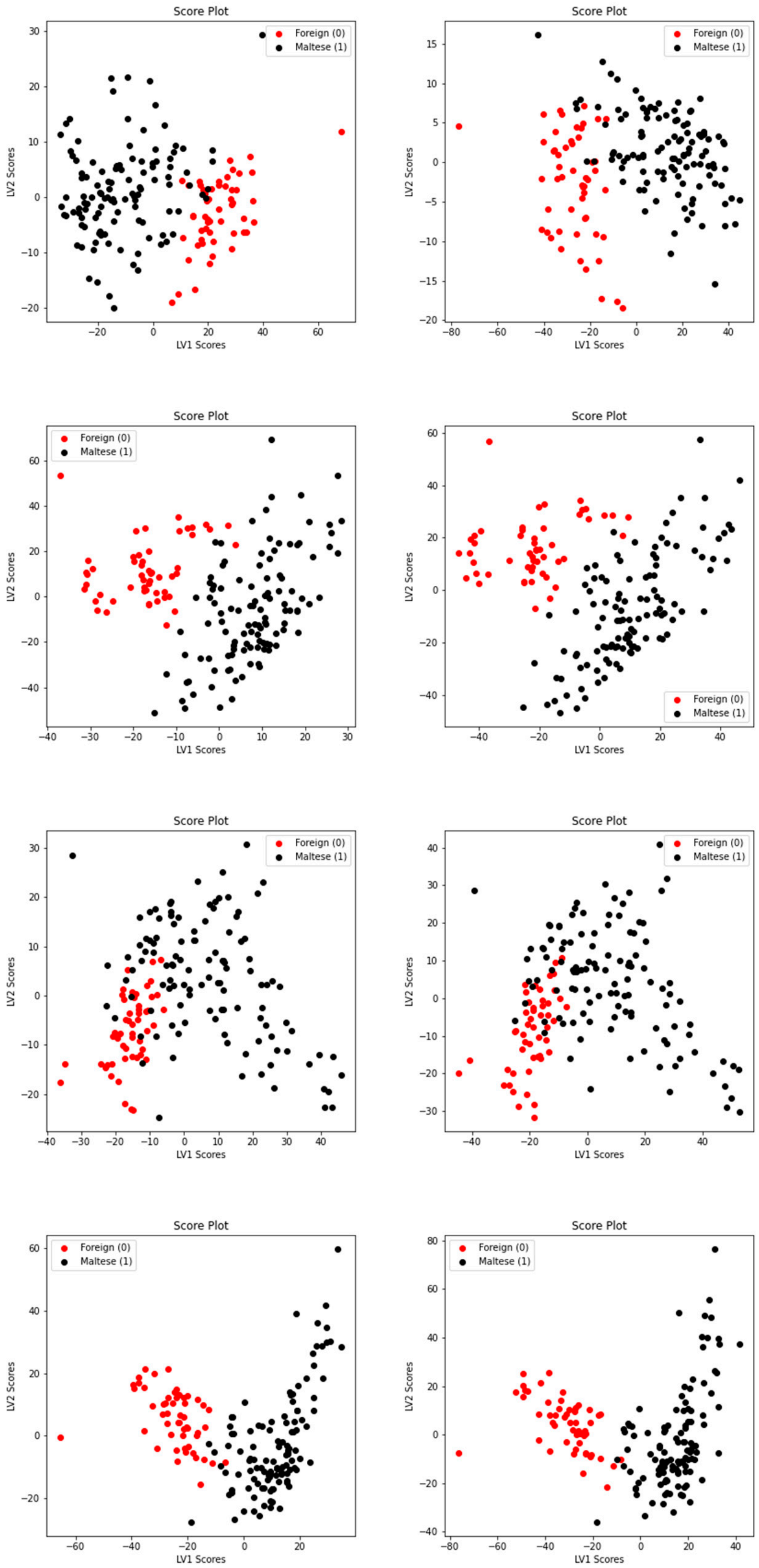
Score Plot
Foreign (0)
Maltese (1)
LV1 Scores
LV2 Scores

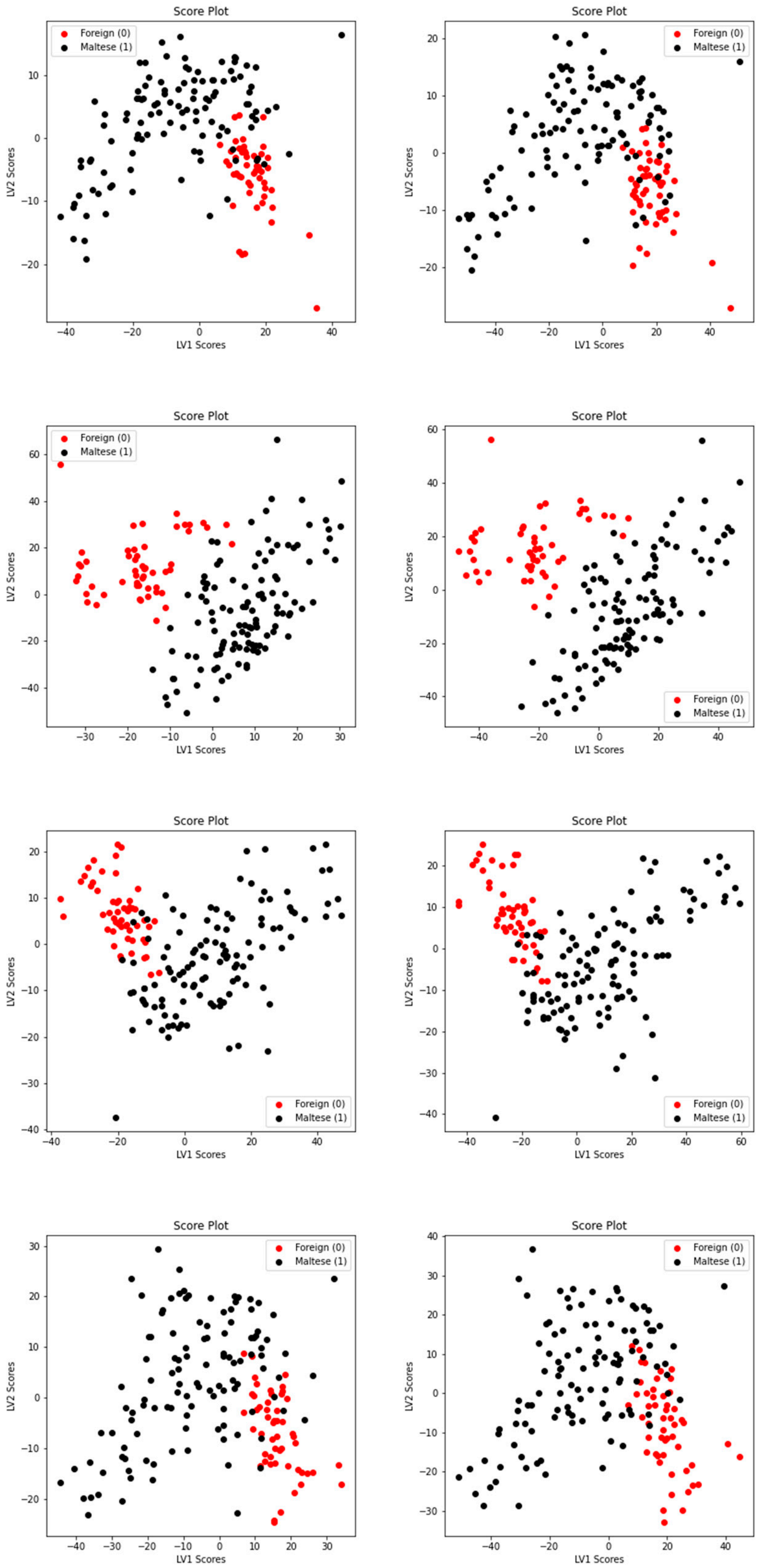
Score Plot
Foreign (0)
Maltese (1)
LV1 Scores
LV2 Scores
Score Plot
Foreign (0)
Maltese (1)
LV1 Scores
LV2 Scores
Score Plot
Foreign (0)
Maltese (1)
LV1 Scores
LV2 Scores
Score Plot
Foreign (0)
Maltese (1)
LV1 Scores
LV2 Scores
Score Plot
Foreign (0)
Maltese (1)
LV1 Scores
LV2 Scores
Score Plot
Foreign (0)
Maltese (1)
LV1 Scores
LV2 Scores
Score Plot
Foreign (0)
Maltese (1)
LV1 Scores
LV2 Scores
Score Plot
Foreign (0)
Maltese (1)
LV1 Scores
LV2 Scores

Figure S11: β-regression coefficient plots of the full spectrum (4000–400 cm⁻¹) for all applied spectral transformations. Rows correspond to preprocessing methods: 1st Derivative, 2nd Derivative, Deresolve, Detrend, Median Filter, Multiplicative Scatter Correction (MSC), Orthogonal Signal Correction (OSC), Quantile Normalization, Raw, Standard Normal Variate (SNV), and SNV+Detrend.

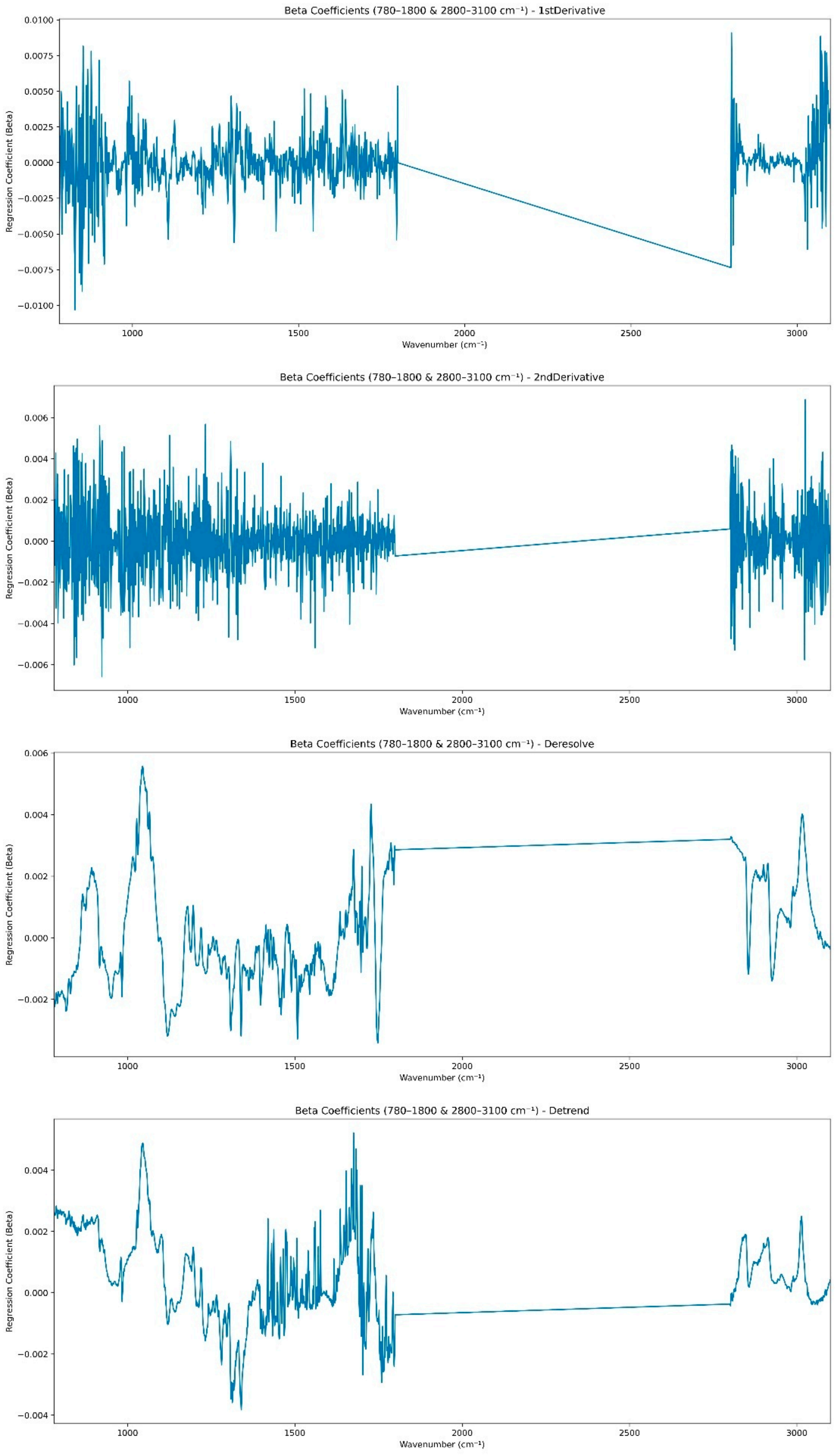

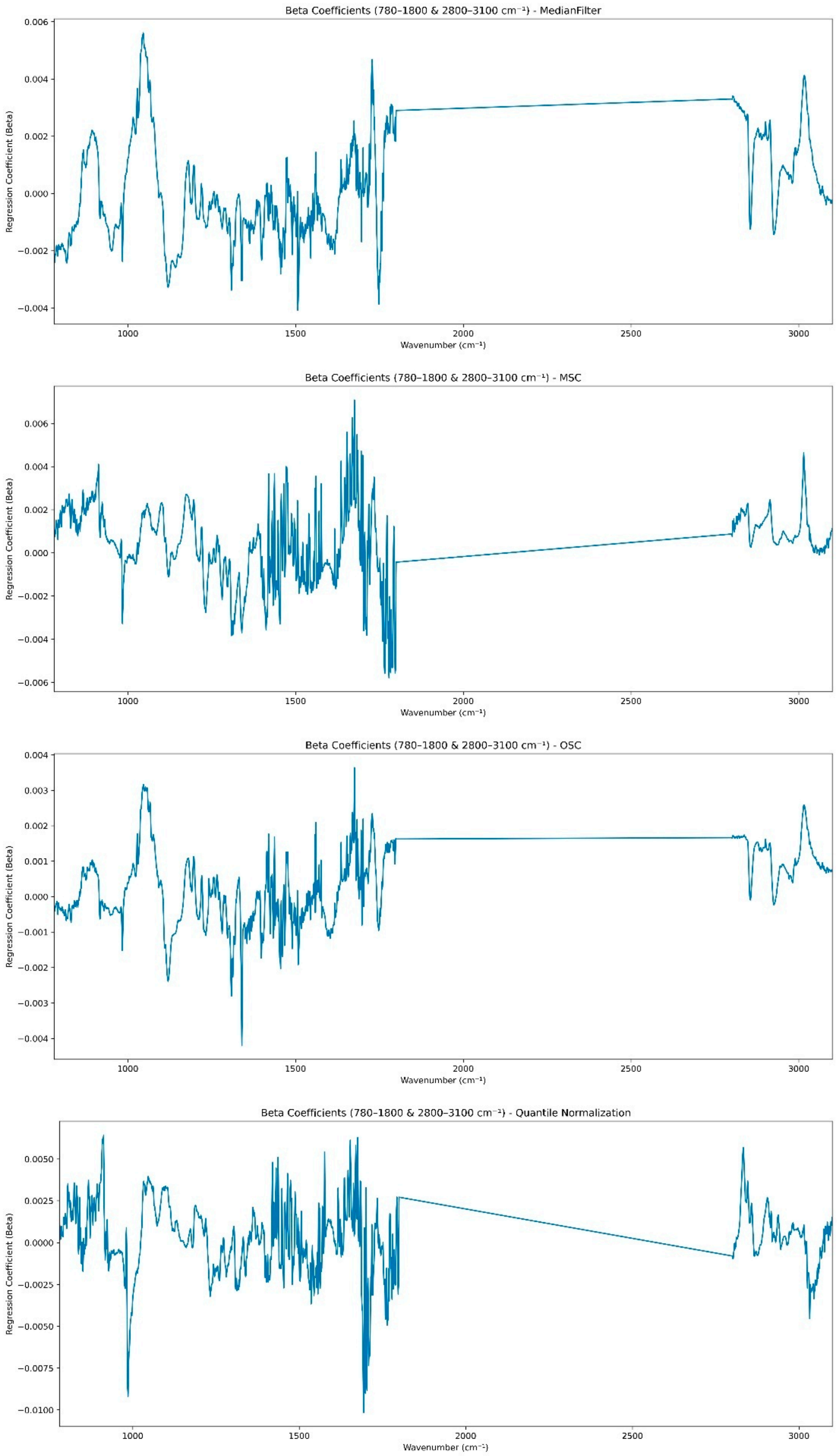
Beta Coefficients (780–1800 & 2800–3100 cm⁻¹) - MedianFilter
Regression Coefficient (Beta)
Wavenumber (cm⁻¹)
Beta Coefficients (780–1800 & 2800–3100 cm⁻¹) - MSC
Regression Coefficient (Beta)
Wavenumber (cm⁻¹)
Beta Coefficients (780–1800 & 2800–3100 cm⁻¹) - OSC
Regression Coefficient (Beta)
Wavenumber (cm⁻¹)
Beta Coefficients (780–1800 & 2800–3100 cm⁻¹) - Quantile Normalization
Regression Coefficient (Beta)
Wavenumber (cm⁻¹)

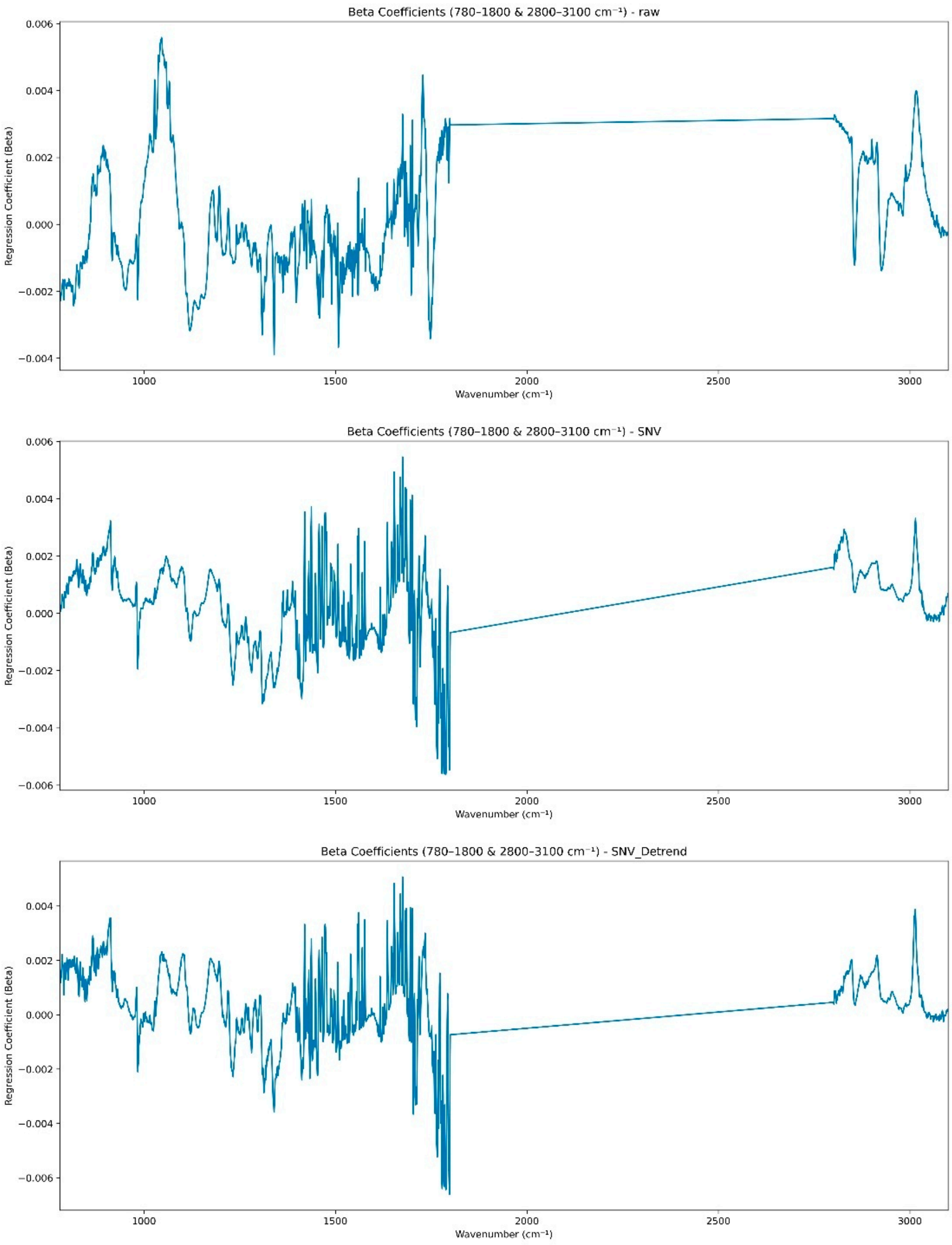

Beta Coefficients (780–1800 & 2800–3100 cm⁻¹) - raw
Regression Coefficient (Beta)
Wavenumber (cm⁻¹)
0.006
0.004
0.002
0.000
−0.002
−0.004
1000
1500
2000
2500
3000
Beta Coefficients (780–1800 & 2800–3100 cm⁻¹) - SNV
Regression Coefficient (Beta)
Wavenumber (cm⁻¹)
0.006
0.004
0.002
0.000
−0.002
−0.004
−0.006
1000
1500
2000
2500
3000
Beta Coefficients (780–1800 & 2800–3100 cm⁻¹) - SNV_Detrend
Regression Coefficient (Beta)
Wavenumber (cm⁻¹)
0.004
0.002
0.000
−0.002
−0.004
−0.006
1000
1500
2000
2500
3000

Figure S12: β-regression coefficient plots of the fingerprint region for all applied spectral transformations. Rows correspond to preprocessing methods: 1st Derivative, 2nd Derivative, Deresolve, Detrend, Median Filter, Multiplicative Scatter Correction (MSC), Orthogonal Signal Correction (OSC), Quantile Normalization, Raw, Standard Normal Variate (SNV), and SNV+Detrend.

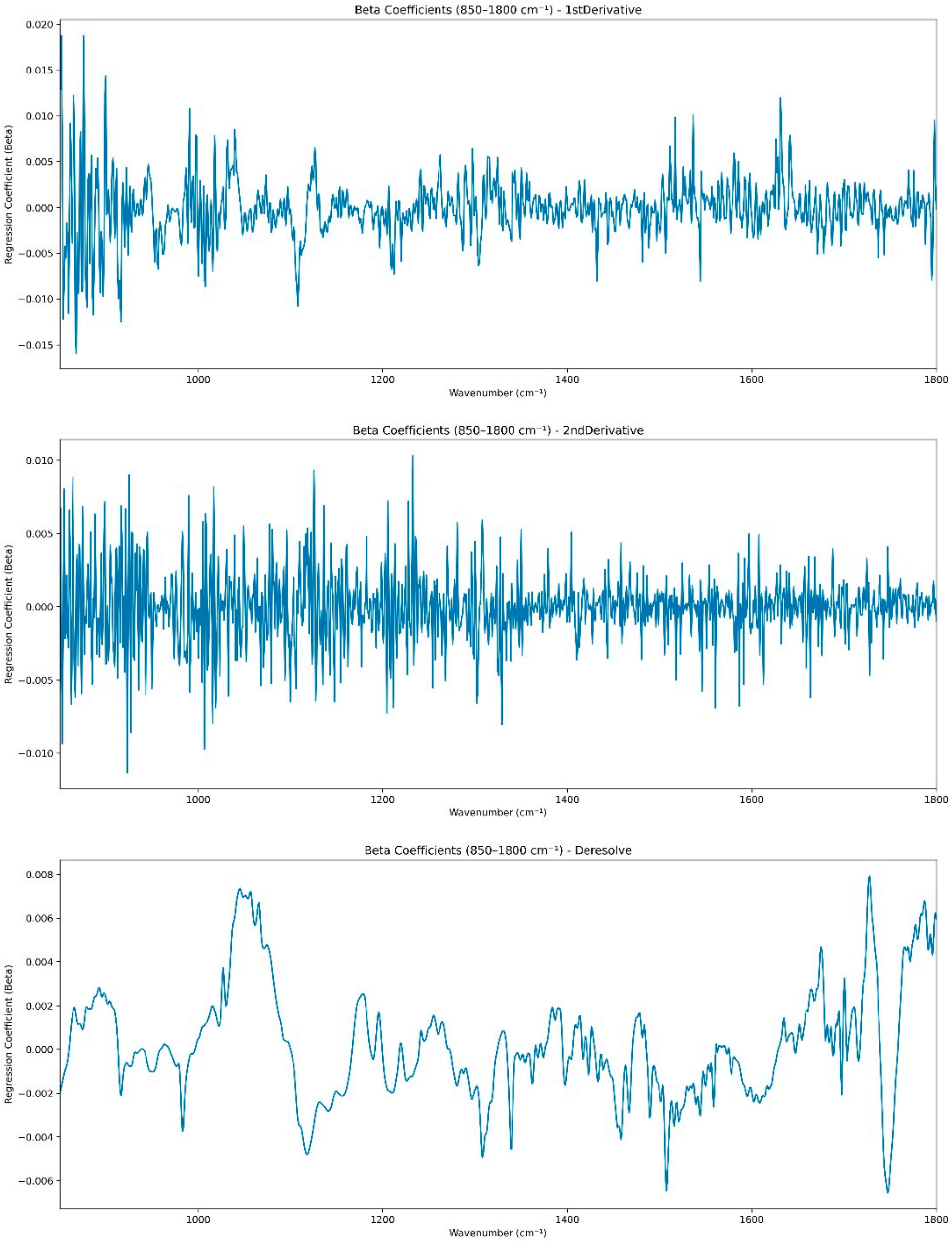

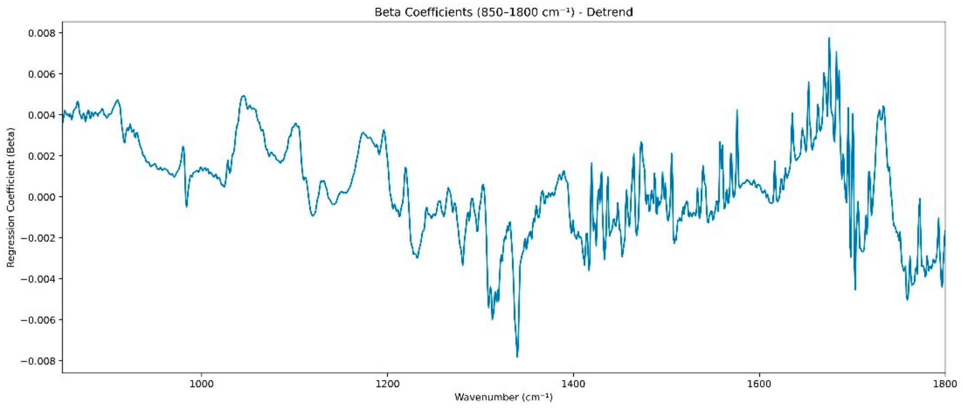
Beta Coefficients (850–1800 cm⁻¹) - Detrend
Regression Coefficient (Beta)
Wavenumber (cm⁻¹)
0.008
0.006
0.004
0.002
0.000
−0.002
−0.004
−0.006
−0.008
1000
1200
1400
1600
1800

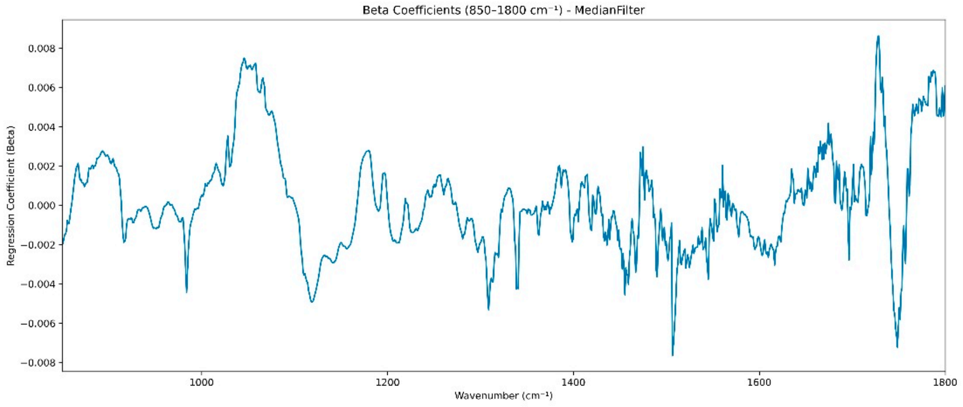
Beta Coefficients (850–1800 cm⁻¹) - MedianFilter
Regression Coefficient (Beta)
Wavenumber (cm⁻¹)
0.008
0.006
0.004
0.002
0.000
−0.002
−0.004
−0.006
−0.008
1000
1200
1400
1600
1800

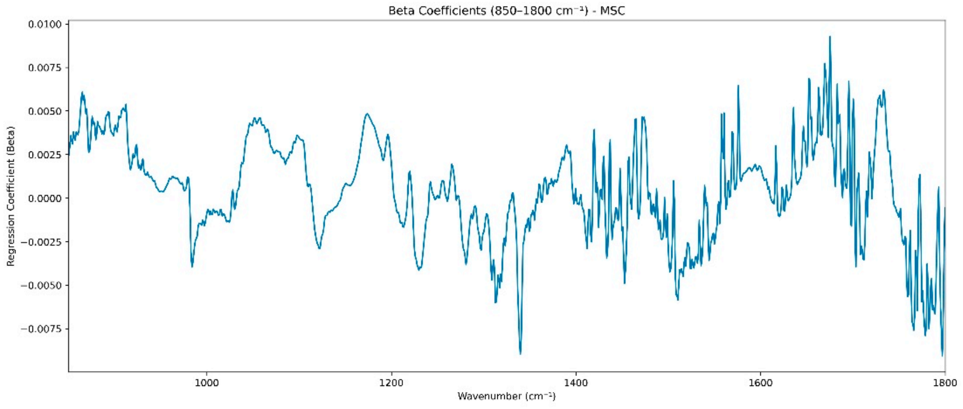
Beta Coefficients (850–1800 cm⁻¹) - MSC
Regression Coefficient (Beta)
Wavenumber (cm⁻¹)
0.0100
0.0075
0.0050
0.0025
0.0000
−0.0025
−0.0050
−0.0075
1000
1200
1400
1600
1800

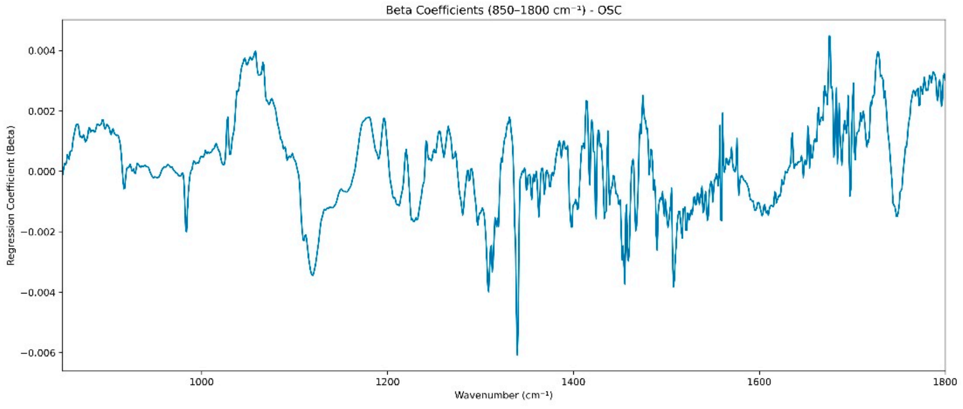
Beta Coefficients (850–1800 cm⁻¹) - OSC
Regression Coefficient (Beta)
Wavenumber (cm⁻¹)
0.004
0.002
0.000
−0.002
−0.004
−0.006
1000
1200
1400
1600
1800

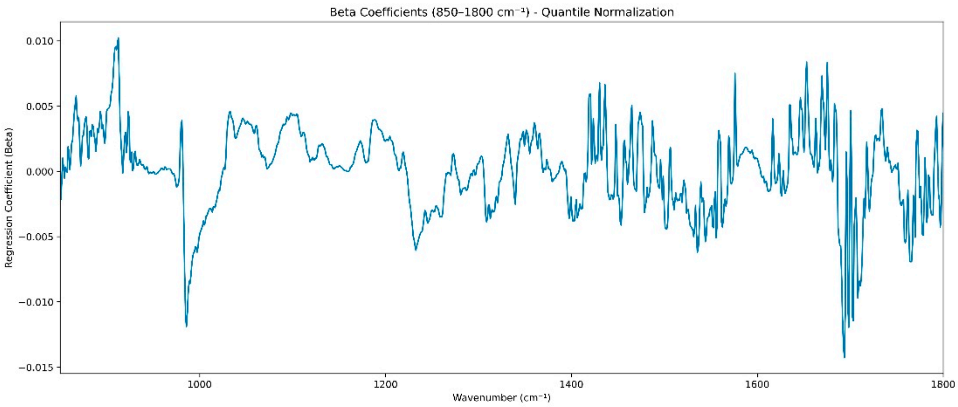
Beta Coefficients (850–1800 cm⁻¹) - Quantile Normalization
Regression Coefficient (Beta)
0.010
0.005
0.000
−0.005
−0.010
−0.015
1000
1200
1400
1600
1800
Wavenumber (cm⁻¹)

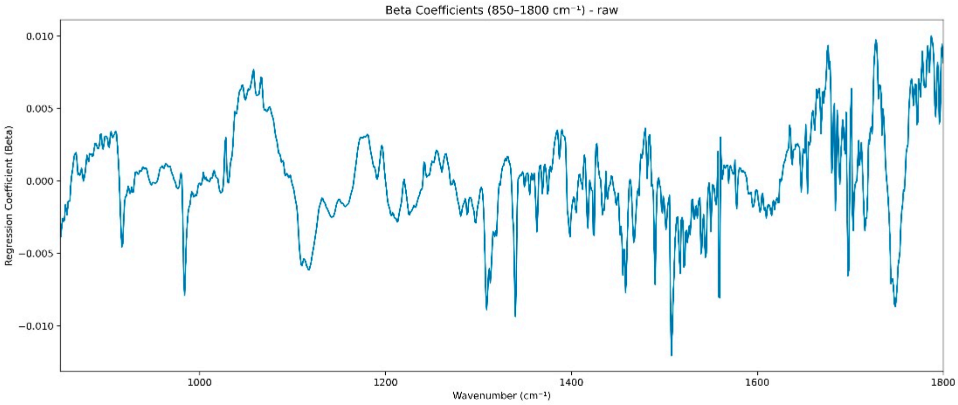
Beta Coefficients (850–1800 cm⁻¹) - raw
Regression Coefficient (Beta)
0.010
0.005
0.000
−0.005
−0.010
1000
1200
1400
1600
1800
Wavenumber (cm⁻¹)

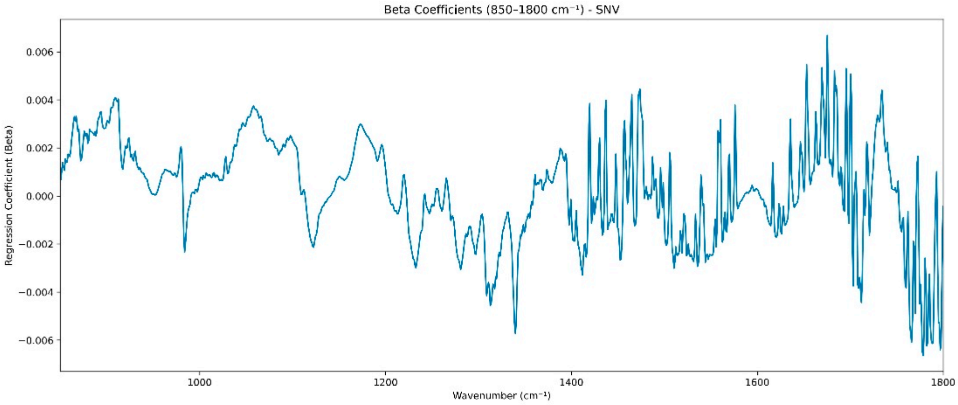
Beta Coefficients (850–1800 cm⁻¹) - SNV
Regression Coefficient (Beta)
0.006
0.004
0.002
0.000
−0.002
−0.004
−0.006
1000
1200
1400
1600
1800
Wavenumber (cm⁻¹)

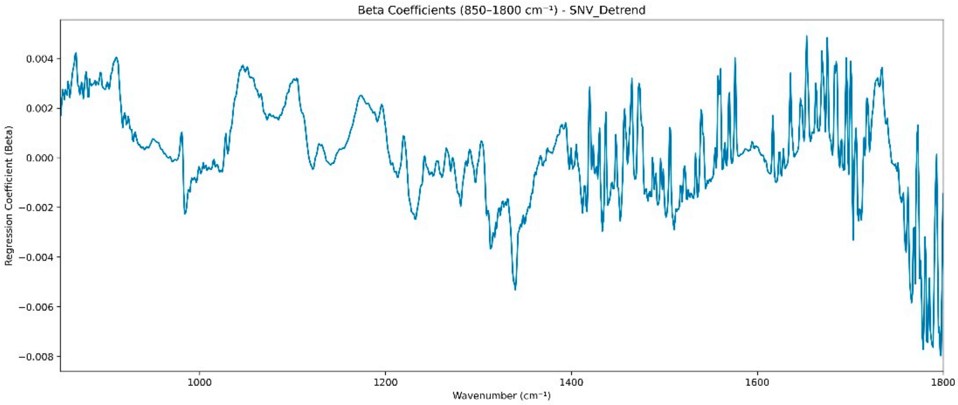
Beta Coefficients (850–1800 cm⁻¹) - SNV_Detrend
Regression Coefficient (Beta)
0.004
0.002
0.000
−0.002
−0.004
−0.006
−0.008
1000
1200
1400
1600
1800
Wavenumber (cm⁻¹)

Figure S13. VIP plots (blue) of the fingerprint region for all applied spectral transformations. Rows correspond to preprocessing methods: 1st Derivative, 2nd Derivative, Deresolve, Detrend, Median Filter, Multiplicative Scatter Correction (MSC), Orthogonal Signal Correction (OSC), Quantile Normalization, Raw, Standard Normal Variate (SNV), and SNV+Detrend for the local (Black) and foreign (Red)

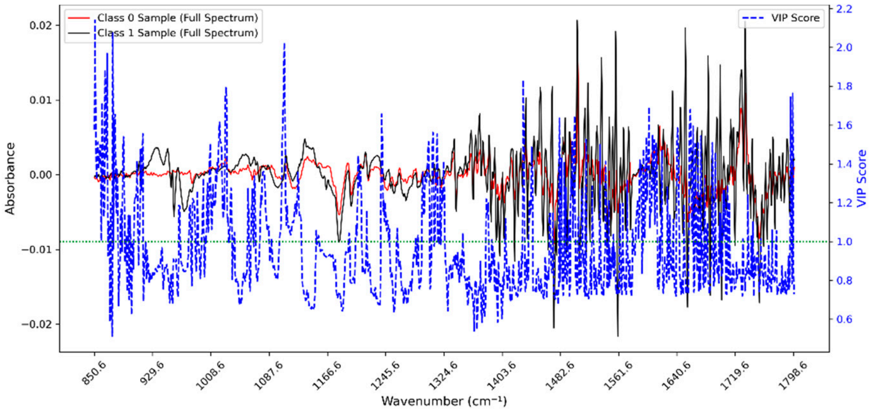

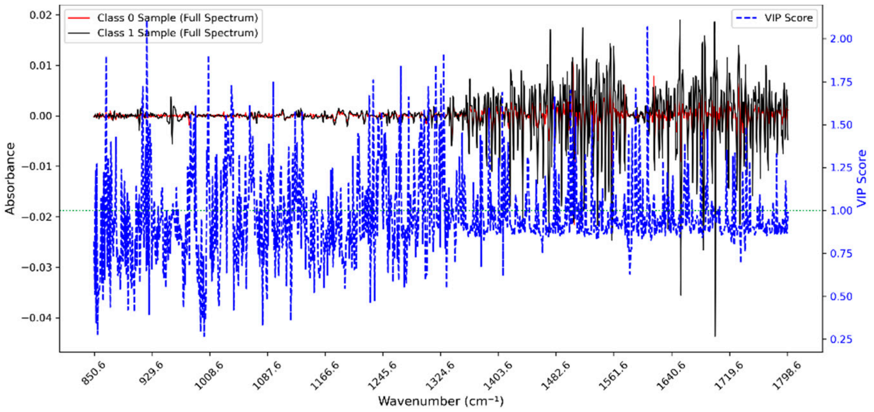

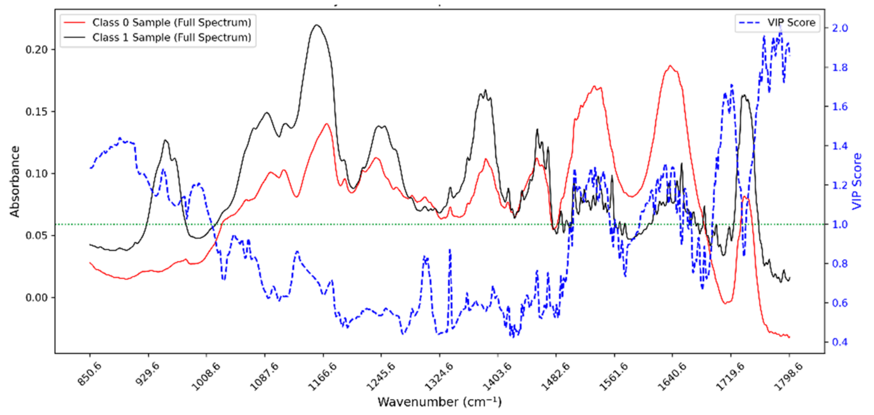

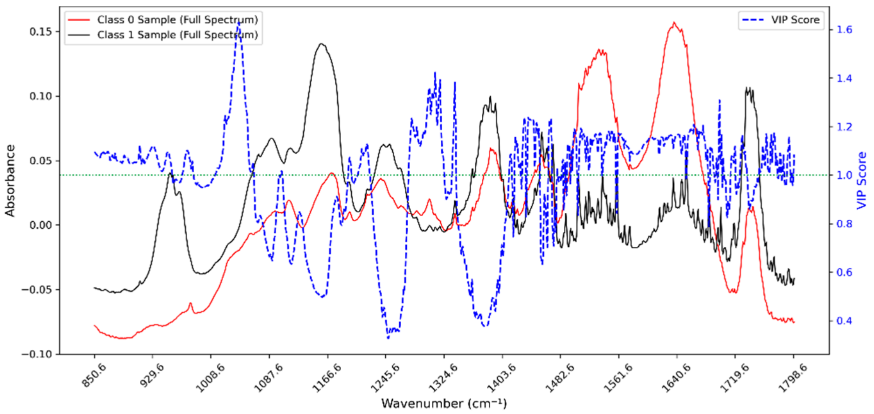
Class 0 Sample (Full Spectrum)
Class 1 Sample (Full Spectrum)
VIP Score
Absorbance
VIP Score
Wavenumber (cm⁻¹)

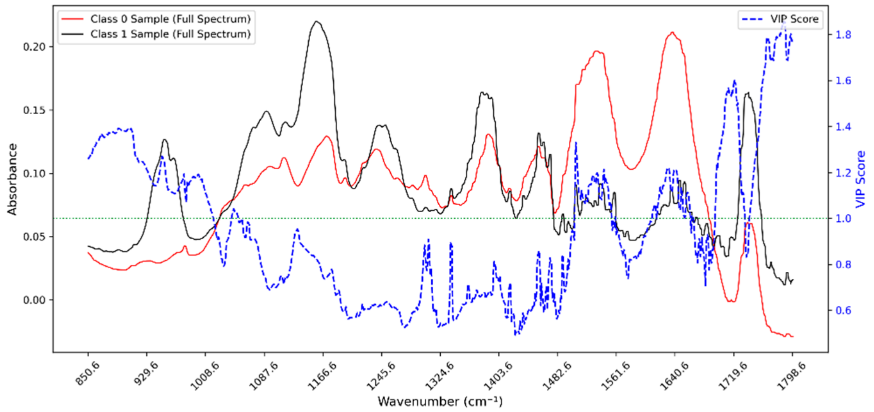
Class 0 Sample (Full Spectrum)
Class 1 Sample (Full Spectrum)
VIP Score
Absorbance
VIP Score
Wavenumber (cm⁻¹)

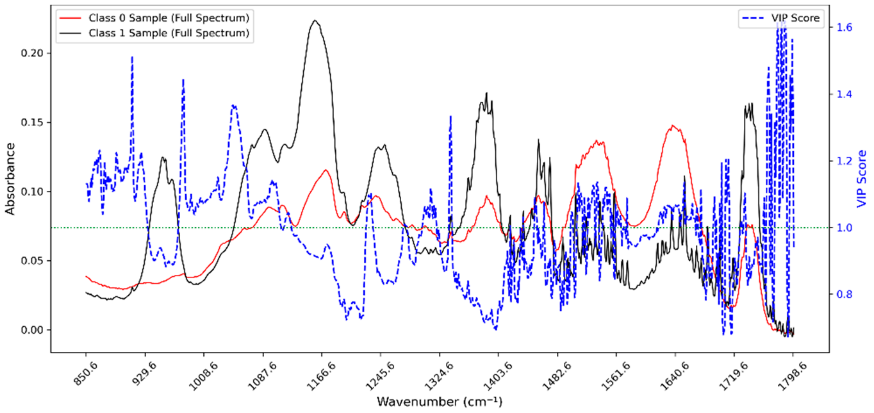
Class 0 Sample (Full Spectrum)
Class 1 Sample (Full Spectrum)
VIP Score
Absorbance
VIP Score
Wavenumber (cm⁻¹)

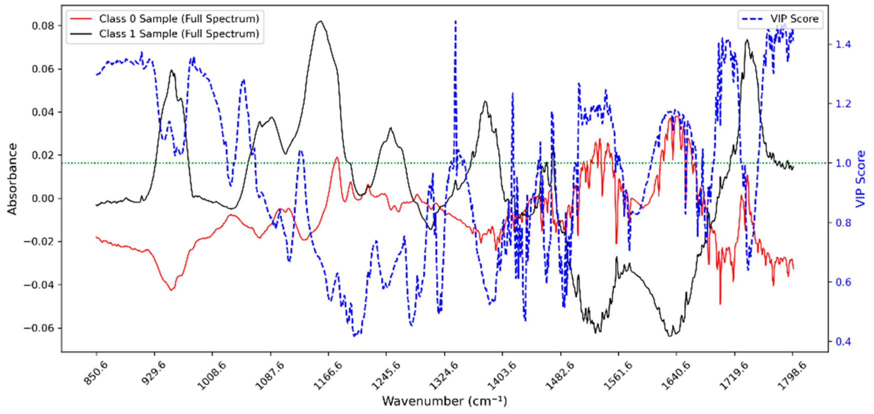
Class 0 Sample (Full Spectrum)
Class 1 Sample (Full Spectrum)
VIP Score
Absorbance
VIP Score
Wavenumber (cm⁻¹)

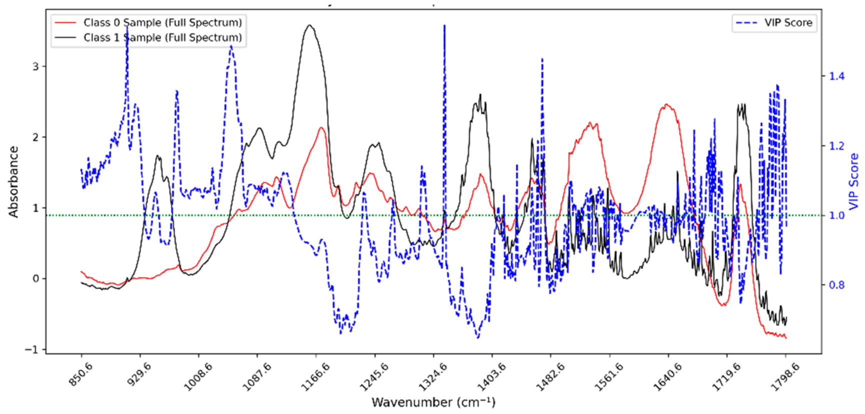
Class 0 Sample (Full Spectrum)
Class 1 Sample (Full Spectrum)
VIP Score
Absorbance
VIP Score
Wavenumber (cm⁻¹)
850.6
929.6
1008.6
1087.6
1166.6
1245.6
1324.6
1403.6
1482.6
1561.6
1640.6
1719.6
1798.6

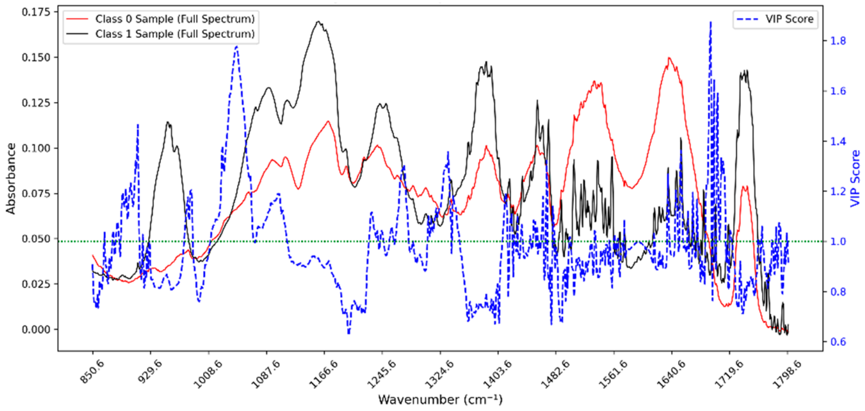
Class 0 Sample (Full Spectrum)
Class 1 Sample (Full Spectrum)
VIP Score
Absorbance
VIP Score
Wavenumber (cm⁻¹)
850.6
929.6
1008.6
1087.6
1166.6
1245.6
1324.6
1403.6
1482.6
1561.6
1640.6
1719.6
1798.6

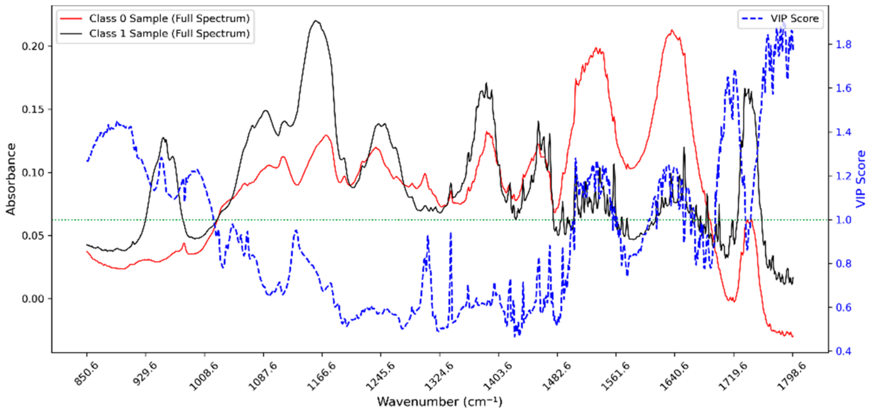
Class 0 Sample (Full Spectrum)
Class 1 Sample (Full Spectrum)
VIP Score
Absorbance
VIP Score
Wavenumber (cm⁻¹)
850.6
929.6
1008.6
1087.6
1166.6
1245.6
1324.6
1403.6
1482.6
1561.6
1640.6
1719.6
1798.6

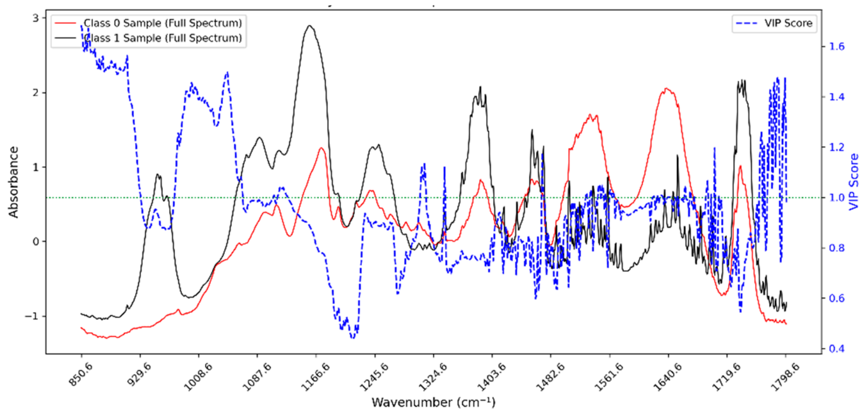
Class 0 Sample (Full Spectrum)
Class 1 Sample (Full Spectrum)
VIP Score
Absorbance
VIP Score
Wavenumber (cm⁻¹)
850.6
929.6
1008.6
1087.6
1166.6
1245.6
1324.6
1403.6
1482.6
1561.6
1640.6
1719.6
1798.6

Figure S14: . Top 30 feature importance plots for all spectral transformations, comparing the *whole spectrum* (left panels) and the *fingerprint region* (right panels). Rows correspond to different preprocessing methods applied to the ATR-FTIR pork dataset: 1st Derivative, 2nd Derivative, Deresolve, Detrend, Median Filter, Multiplicative Scatter Correction (MSC), Orthogonal Signal Correction (OSC), Quantile Normalization, Raw, Standard Normal Variate (SNV), and SNV+Detrend.

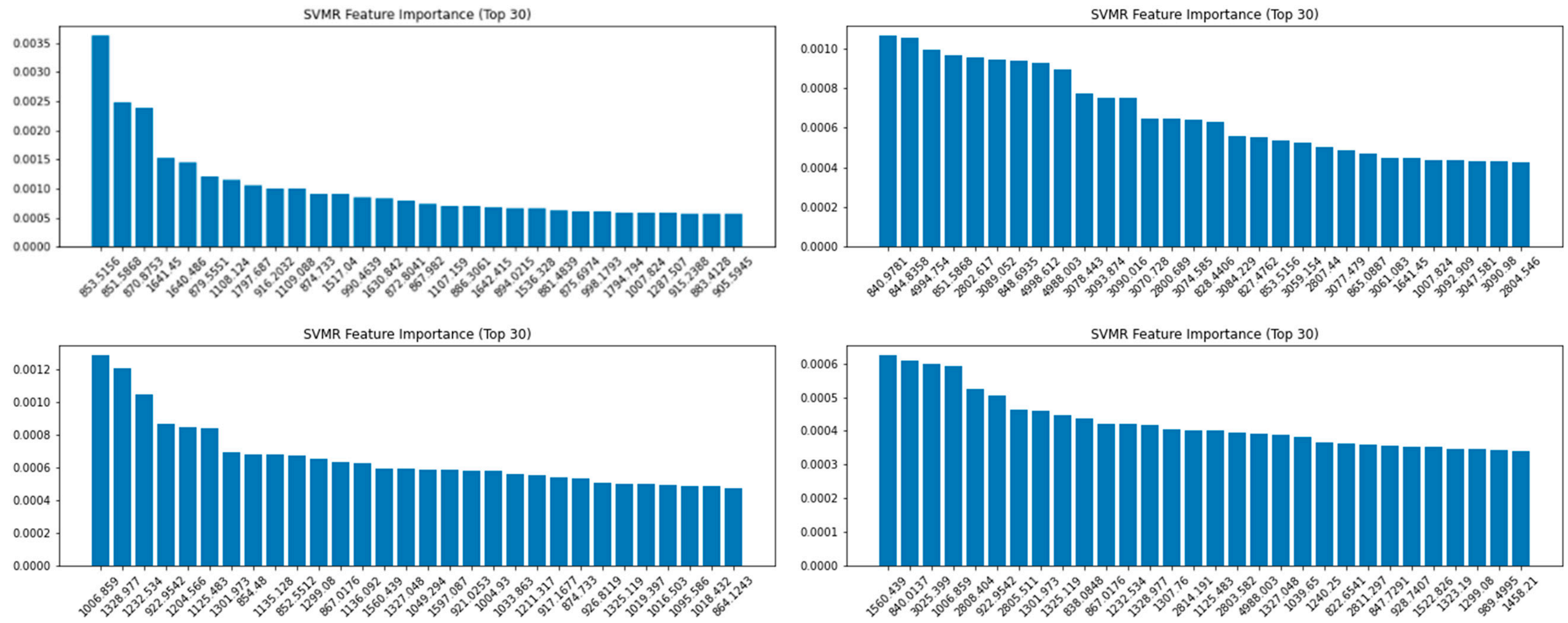

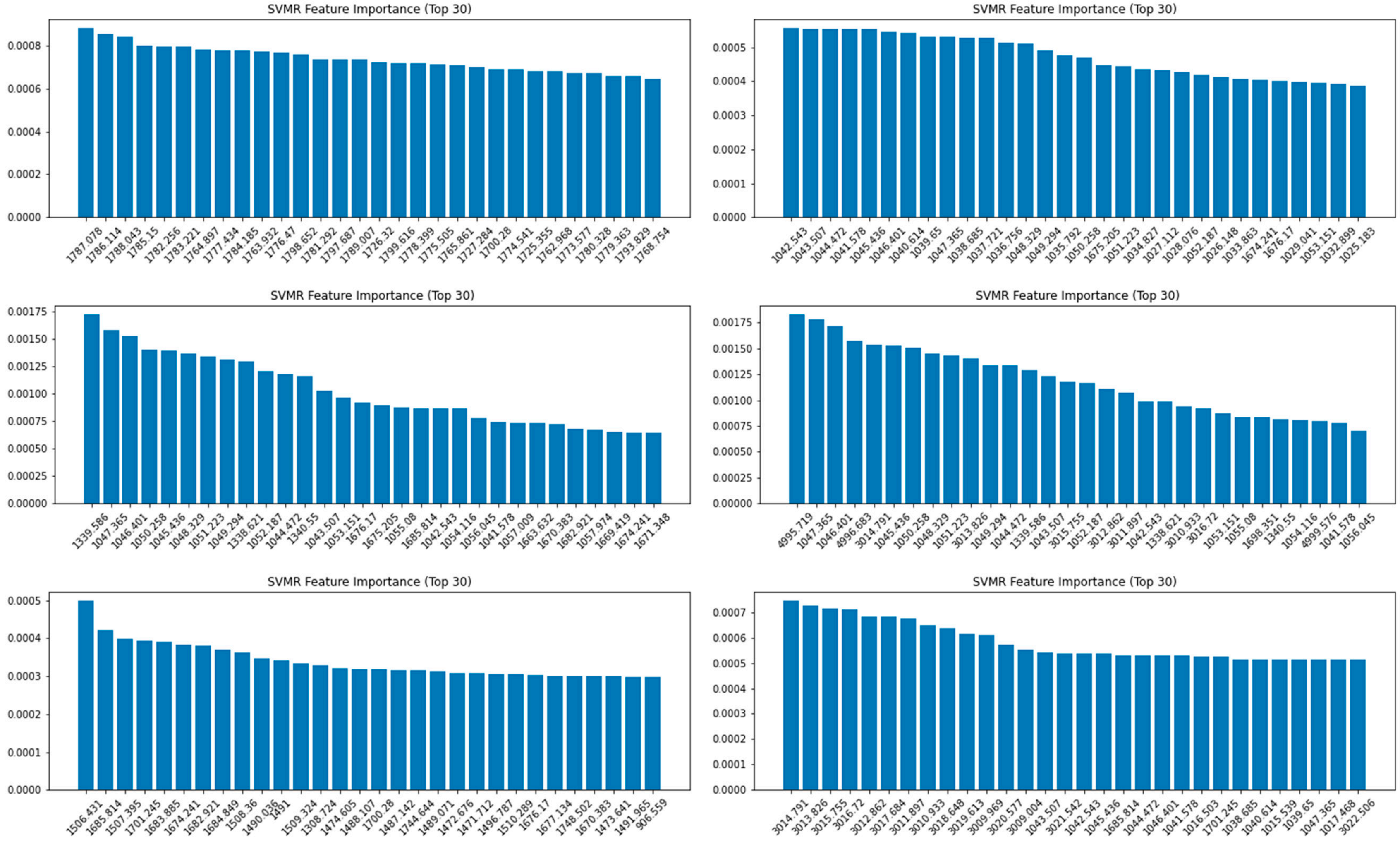
SVMR Feature Importance (Top 30)
0.0000 0.0002 0.0004 0.0006 0.0008
1787.078 1786.114 1788.043 1785.15 1782.256 1783.221 1764.897 1777.434 1784.185 1763.932 1776.47 1798.652 1781.292 1797.687 1789.007 1726.32 1799.616 1778.399 1775.505 1765.861 1727.284 1700.28 1774.541 1725.355 1762.968 1773.577 1780.328 1779.363 1793.829 1768.754
SVMR Feature Importance (Top 30)
0.0000 0.0001 0.0002 0.0003 0.0004 0.0005
1042.543 1043.507 1044.472 1041.578 1045.436 1046.401 1040.614 1039.65 1047.365 1038.685 1037.721 1036.756 1048.329 1049.294 1035.792 1050.258 1675.205 1051.223 1034.827 1027.112 1028.076 1052.187 1026.148 1033.863 1674.241 1676.17 1029.041 1053.151 1032.899 1025.183
SVMR Feature Importance (Top 30)
0.00000 0.00025 0.00050 0.00075 0.00100 0.00125 0.00150 0.00175
1339.586 1047.365 1046.401 1050.258 1045.436 1048.329 1051.223 1049.294 1338.621 1052.187 1044.472 1340.55 1043.507 1053.151 1676.17 1675.205 1055.08 1685.814 1042.543 1054.116 1056.045 1041.578 1057.009 1663.632 1670.383 1682.921 1057.974 1669.419 1674.241 1671.348
SVMR Feature Importance (Top 30)
0.00000 0.00025 0.00050 0.00075 0.00100 0.00125 0.00150 0.00175
4995.719 1047.365 1046.401 4996.683 3014.791 1045.436 1050.258 1048.329 1051.223 3013.826 1049.294 1044.472 1339.586 1043.507 3015.755 1052.187 3012.862 3011.897 1042.543 1338.621 3010.933 3016.72 1053.151 1055.08 1698.351 1340.55 1054.116 4999.576 1041.578 1056.045
SVMR Feature Importance (Top 30)
0.0000 0.0001 0.0002 0.0003 0.0004 0.0005
1506.431 1685.814 1507.395 1701.245 1683.885 1674.241 1682.921 1684.849 1508.36 1490.036 1491 1509.324 1308.724 1474.605 1488.107 1700.28 1487.142 1744.644 1489.071 1472.676 1471.712 1496.787 1510.289 1676.17 1677.134 1748.502 1670.383 1473.641 1491.965 906.559
SVMR Feature Importance (Top 30)
0.0000 0.0001 0.0002 0.0003 0.0004 0.0005 0.0006 0.0007
3014.791 3013.826 3015.755 3016.72 3012.862 3017.684 3011.897 3010.933 3018.648 3019.613 3009.969 3020.577 3009.004 1043.507 3021.542 1042.543 1045.436 1685.814 1044.472 1046.401 1041.578 1016.503 1701.245 1038.685 1040.614 1015.539 1039.65 1047.365 1017.468 3022.506

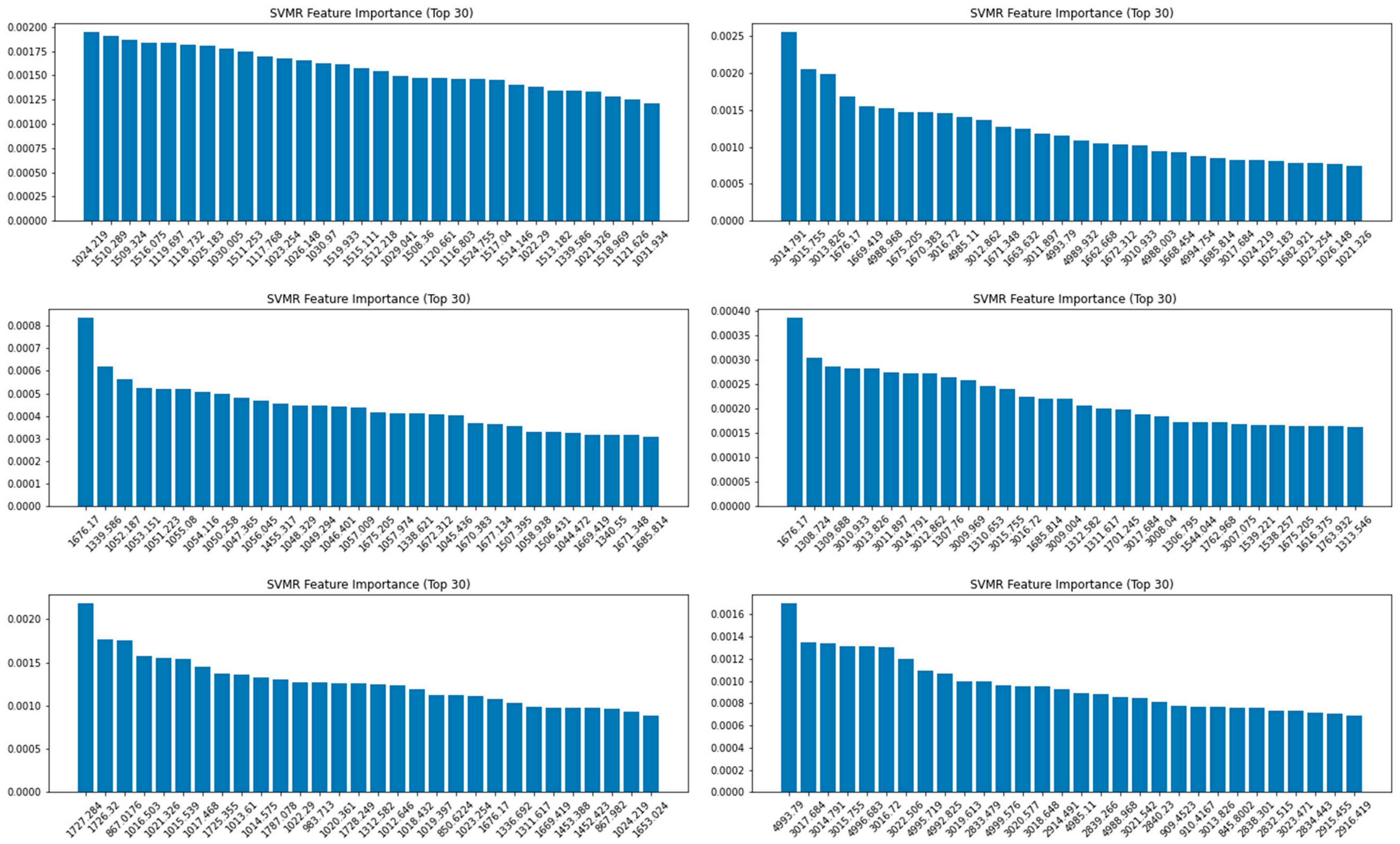
SVMR Feature Importance (Top 30)
1024.219
1510.289
1509.324
1516.075
1119.697
1118.732
1025.183
1030.005
1511.253
1117.768
1023.254
1026.148
1030.97
1519.933
1515.111
1512.218
1029.041
1508.36
1120.661
1116.803
1524.755
1517.04
1514.146
1022.29
1513.182
1339.586
1021.326
1518.969
1121.626
1031.934
SVMR Feature Importance (Top 30)
3014.791
3015.755
3013.826
1676.17
1669.419
4988.968
1675.205
1670.383
3016.72
4985.11
3012.862
1671.348
1663.632
3011.897
4993.79
4989.932
1662.668
1672.312
3010.933
4988.003
1668.454
4994.754
1685.814
3017.684
1024.219
1025.183
1682.921
1023.254
1026.148
1021.326
SVMR Feature Importance (Top 30)
1676.17
1339.586
1052.187
1053.151
1051.223
1055.08
1054.116
1050.258
1047.365
1056.045
1455.317
1048.329
1049.294
1046.401
1057.009
1675.205
1057.974
1338.621
1672.312
1045.436
1670.383
1677.134
1507.395
1058.938
1506.431
1044.472
1669.419
1340.55
1671.348
1685.814
SVMR Feature Importance (Top 30)
1676.17
1308.724
1309.688
3010.933
3013.826
3011.897
3014.791
3012.862
1307.76
3009.969
1310.653
3015.755
3016.72
1685.814
3009.004
1312.582
1311.617
1701.245
3017.684
3008.04
1306.795
1544.044
1762.968
3007.075
1539.221
1538.257
1675.205
1616.375
1763.932
1313.546
SVMR Feature Importance (Top 30)
1727.284
1726.32
867.0176
1016.503
1021.326
1015.539
1017.468
1725.355
1013.61
1014.575
1787.078
1022.29
983.713
1020.361
1728.249
1312.582
1012.646
1018.432
1019.397
850.6224
1023.254
1676.17
1336.692
1311.617
1669.419
1453.388
1452.423
867.982
1024.219
1653.024
SVMR Feature Importance (Top 30)
4993.79
3017.684
3014.791
3015.755
4996.683
3016.72
3022.506
4995.719
4992.825
3019.613
2833.479
4999.576
3020.577
3018.648
2914.491
4985.11
2839.266
4988.968
3021.542
2840.23
909.4523
910.4167
3013.826
845.8002
2838.301
2832.515
3023.471
2834.443
2915.455
2916.419

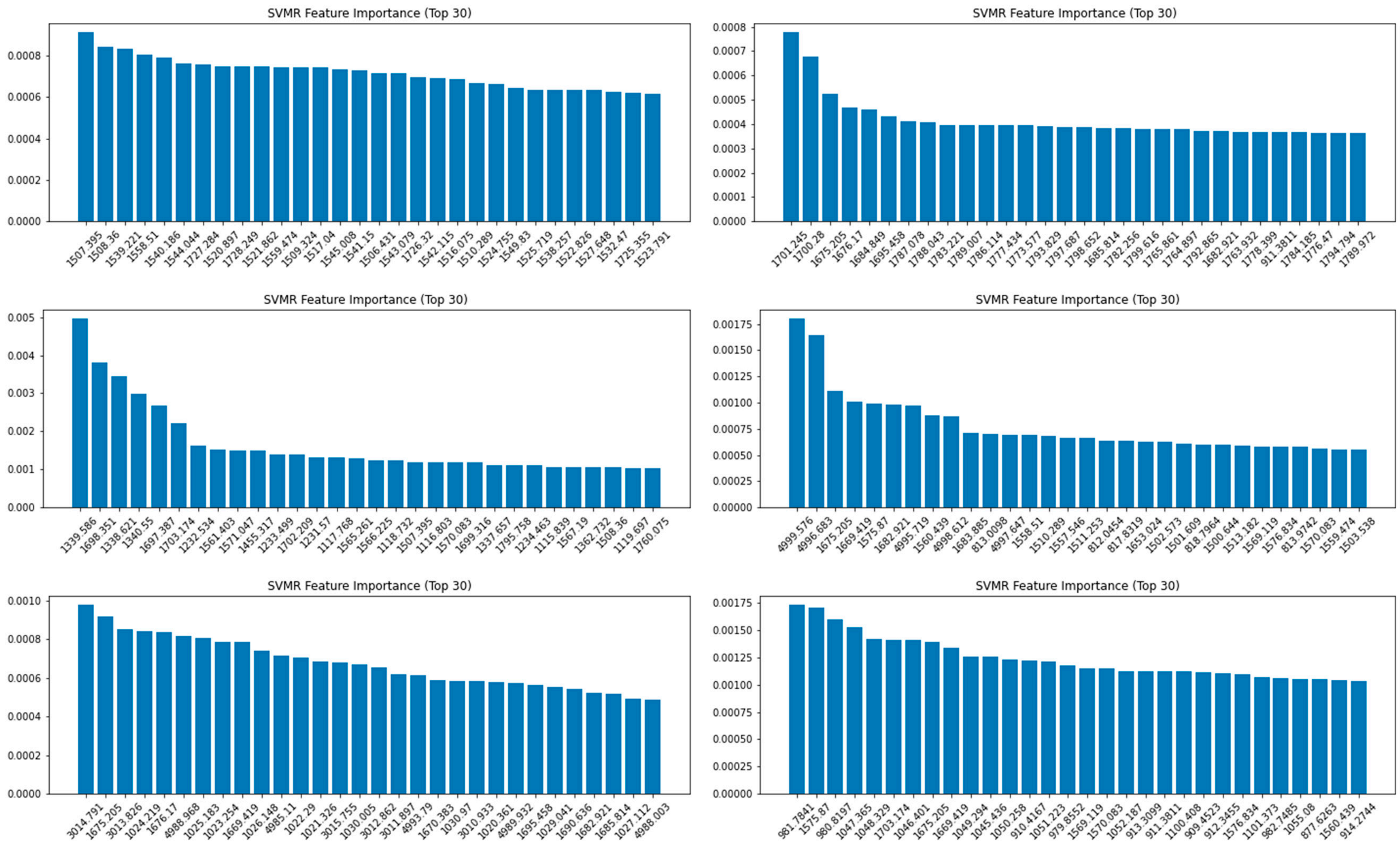
SVMR Feature Importance (Top 30)
0.0000 0.0002 0.0004 0.0006 0.0008
1507.395 1508.36 1539.221 1558.51 1540.186 1544.044 1727.284 1520.897 1728.249 1521.862 1559.474 1509.324 1517.04 1545.008 1541.15 1506.431 1543.079 1726.32 1542.115 1516.075 1510.289 1524.755 1549.83 1525.719 1538.257 1522.826 1527.648 1532.47 1725.355 1523.791
SVMR Feature Importance (Top 30)
0.0000 0.0001 0.0002 0.0003 0.0004 0.0005 0.0006 0.0007 0.0008
1701.245 1700.28 1675.205 1676.17 1684.849 1695.458 1787.078 1788.043 1783.221 1789.007 1786.114 1777.434 1773.577 1793.829 1797.687 1798.652 1685.814 1782.256 1799.616 1765.861 1764.897 1792.865 1682.921 1763.932 1778.399 911.3811 1784.185 1776.47 1794.794 1789.972
SVMR Feature Importance (Top 30)
0.000 0.001 0.002 0.003 0.004 0.005
1339.586 1698.351 1338.621 1340.55 1697.387 1703.174 1232.534 1561.403 1571.047 1455.317 1233.499 1702.209 1231.57 1117.768 1565.261 1566.225 1118.732 1507.395 1116.803 1570.083 1699.316 1337.657 1795.758 1234.463 1115.839 1567.19 1362.732 1508.36 1119.697 1760.075
SVMR Feature Importance (Top 30)
0.00000 0.00025 0.00050 0.00075 0.00100 0.00125 0.00150 0.00175
4999.576 4996.683 1675.205 1669.419 1575.87 1682.921 4995.719 1560.439 4998.612 1683.885 813.0098 4997.647 1558.51 1510.289 1557.546 1511.253 812.0454 817.8319 1653.024 1502.573 1501.609 818.7964 1500.644 1513.182 1569.119 1576.834 813.9742 1570.083 1559.474 1503.538
SVMR Feature Importance (Top 30)
0.0000 0.0002 0.0004 0.0006 0.0008 0.0010
3014.791 1675.205 3013.826 1024.219 1676.17 4988.968 1025.183 1023.254 1669.419 1026.148 4985.11 1022.29 1021.326 3015.755 1030.005 3012.862 3011.897 4993.79 1670.383 1030.97 3010.933 1020.361 4989.932 1695.458 1029.041 1690.636 1682.921 1685.814 1027.112 4988.003
SVMR Feature Importance (Top 30)
0.00000 0.00025 0.00050 0.00075 0.00100 0.00125 0.00150 0.00175
981.7841 1575.87 980.8197 1047.365 1048.329 1703.174 1046.401 1675.205 1669.419 1049.294 1045.436 1050.258 910.4167 1051.223 979.8552 1569.119 1570.083 1052.187 913.3099 911.3811 1100.408 909.4523 912.3455 1576.834 1101.373 982.7485 1055.08 877.6263 1560.439 914.2744

Figure S15. Confusion matrices for classification of Maltese and non-Maltese pork using ANN models with different preprocessing methods: Rows correspond to different preprocessing methods applied to the ATR-FTIR pork dataset: 1st Derivative, 2nd Derivative, Deresolve, Detrend, Median Filter, Multiplicative Scatter Correction (MSC), Orthogonal Signal Correction (OSC), Quantile Normalization, Raw, Standard Normal Variate (SNV), and SNV+Detrend. Left panels represent results from excluded rows (external validation), while right panels represent results from training datasets. Correct classifications are indicated along the diagonal, with misclassifications off-diagonal.

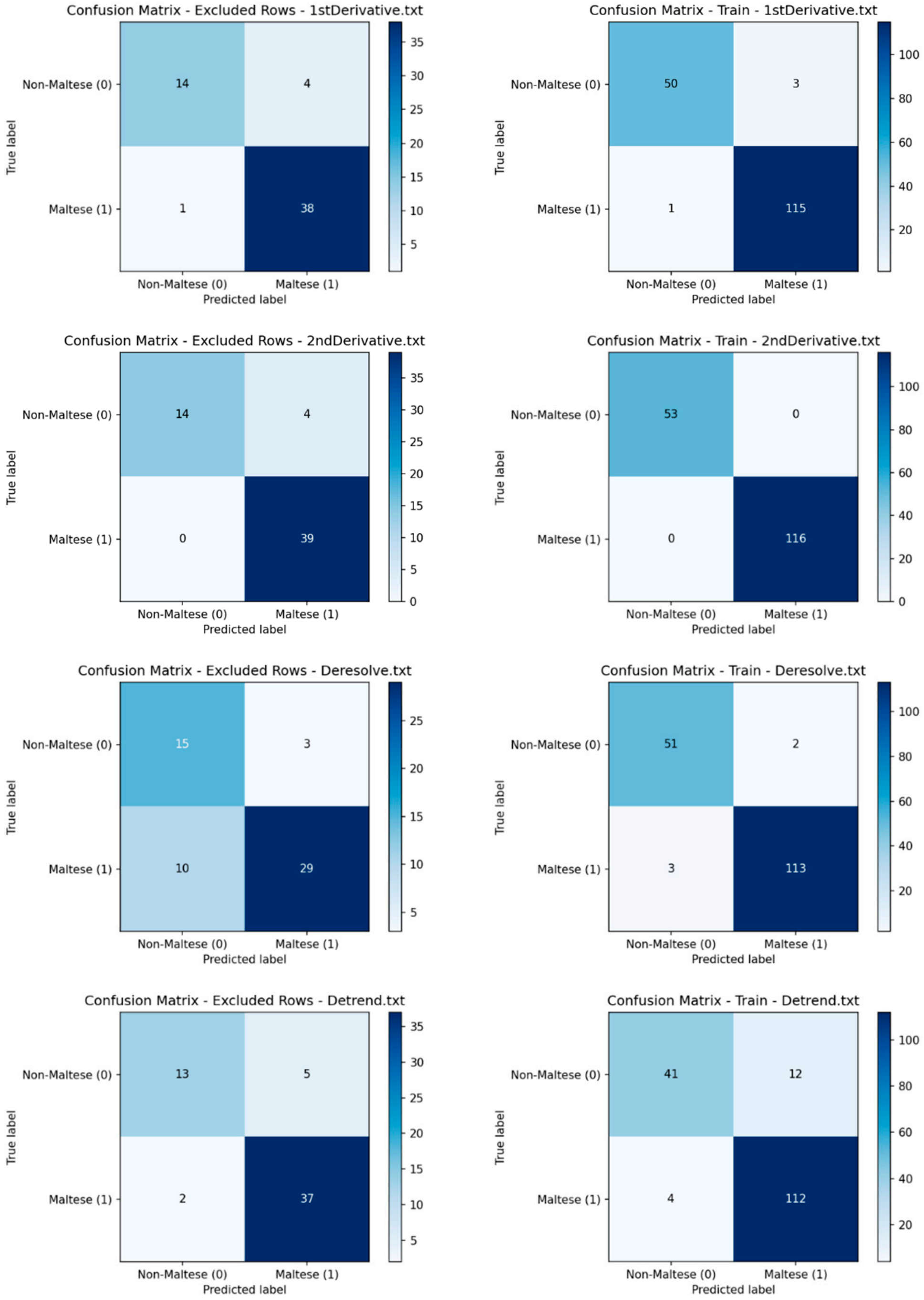

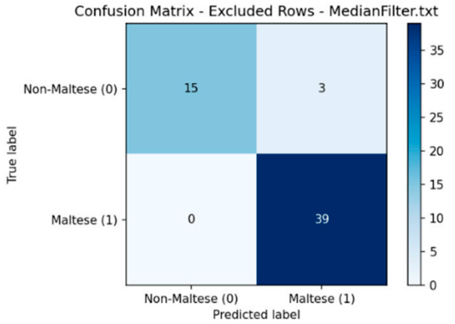
Confusion Matrix - Excluded Rows - MedianFilter.txt
True label
Non-Maltese (0)
Maltese (1)
15
3
0
39
Non-Maltese (0)
Maltese (1)
Predicted label
35 30 25 20 15 10 5 0

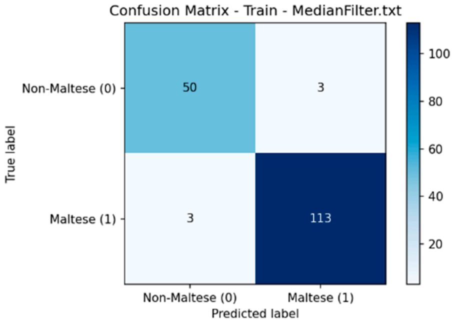
Confusion Matrix - Train - MedianFilter.txt
True label
Non-Maltese (0)
Maltese (1)
50
3
3
113
Non-Maltese (0)
Maltese (1)
Predicted label
100 80 60 40 20

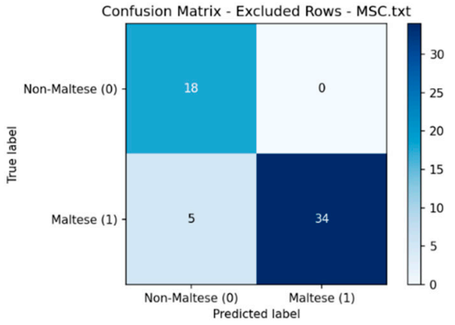
Confusion Matrix - Excluded Rows - MSC.txt
True label
Non-Maltese (0)
Maltese (1)
18
0
5
34
Non-Maltese (0)
Maltese (1)
Predicted label
30 25 20 15 10 5 0

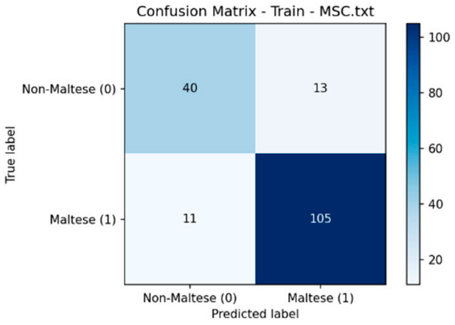
Confusion Matrix - Train - MSC.txt
True label
Non-Maltese (0)
Maltese (1)
40
13
11
105
Non-Maltese (0)
Maltese (1)
Predicted label
100 80 60 40 20

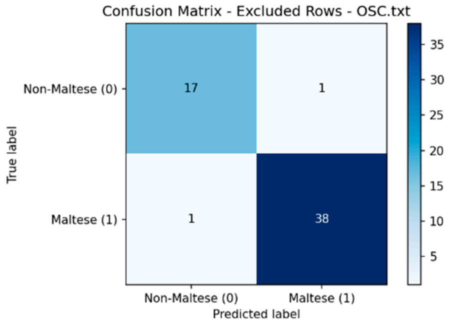
Confusion Matrix - Excluded Rows - OSC.txt
True label
Non-Maltese (0)
Maltese (1)
17
1
1
38
Non-Maltese (0)
Maltese (1)
Predicted label
35 30 25 20 15 10 5

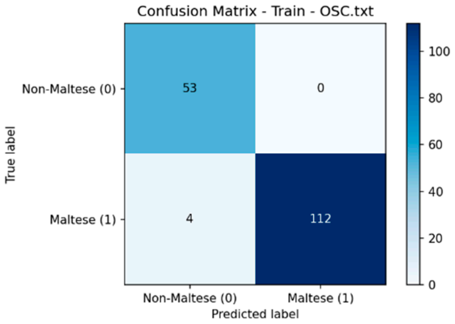
Confusion Matrix - Train - OSC.txt
True label
Non-Maltese (0)
Maltese (1)
53
0
4
112
Non-Maltese (0)
Maltese (1)
Predicted label
100 80 60 40 20 0

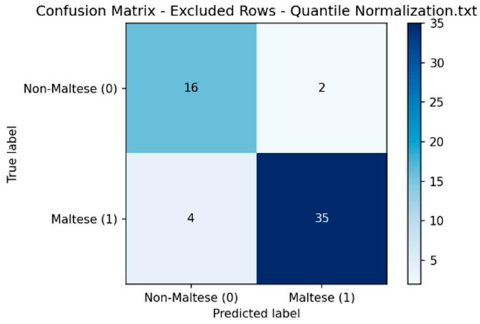
Confusion Matrix - Excluded Rows - Quantile Normalization.txt
True label
Non-Maltese (0)
Maltese (1)
16
2
4
35
Non-Maltese (0)
Maltese (1)
Predicted label
35 30 25 20 15 10 5

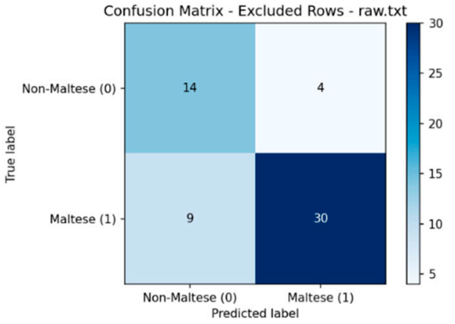
Confusion Matrix - Excluded Rows - raw.txt
True label
Non-Maltese (0)
Maltese (1)
14
4
9
30
Non-Maltese (0)
Maltese (1)
Predicted label
30 25 20 15 10 5

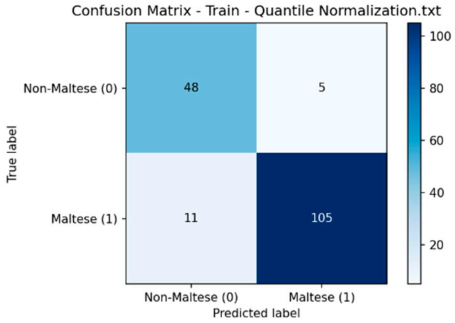
Confusion Matrix - Train - Quantile Normalization.txt
True label
Non-Maltese (0)
Maltese (1)
48
5
11
105
Non-Maltese (0)
Maltese (1)
Predicted label
100 80 60 40 20

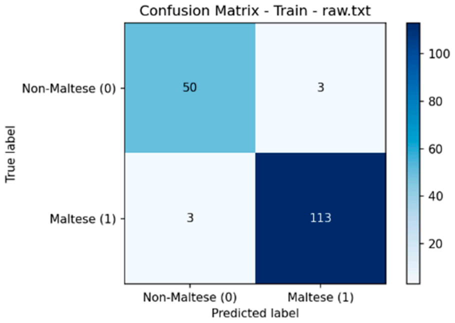
Confusion Matrix - Train - raw.txt
True label
Non-Maltese (0)
Maltese (1)
50
3
3
113
Non-Maltese (0)
Maltese (1)
Predicted label
100 80 60 40 20

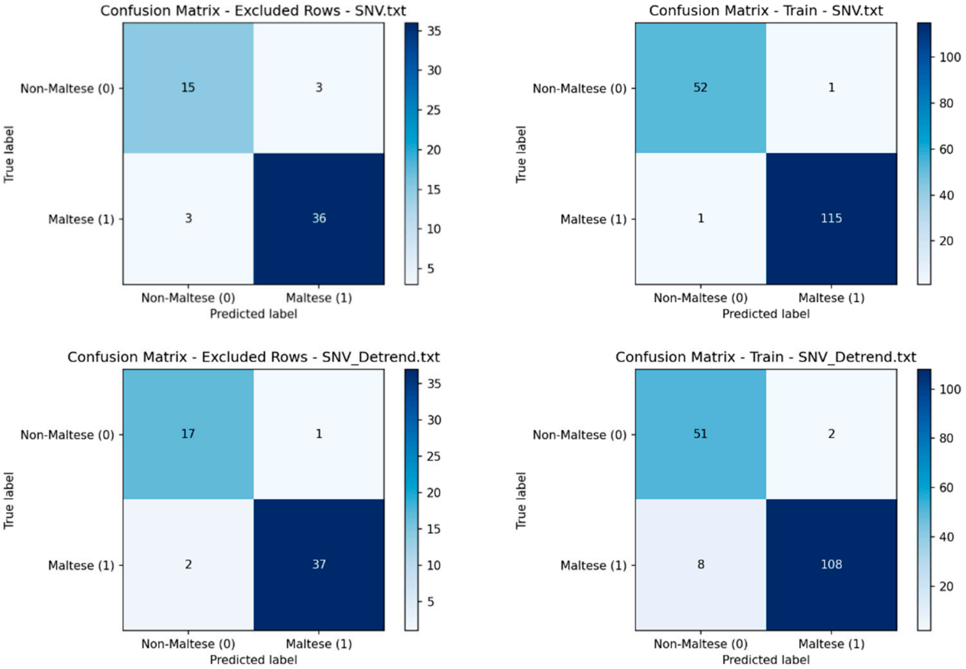

Confusion Matrix - Excluded Rows - SNV.txt
Non-Maltese (0)
Maltese (1)
True label
Predicted label
15
3
3
36
35
30
25
20
15
10
5
Confusion Matrix - Train - SNV.txt
Non-Maltese (0)
Maltese (1)
True label
Predicted label
52
1
1
115
100
80
60
40
20
Confusion Matrix - Excluded Rows - SNV_Detrend.txt
Non-Maltese (0)
Maltese (1)
True label
Predicted label
17
1
2
37
35
30
25
20
15
10
5
Confusion Matrix - Train - SNV_Detrend.txt
Non-Maltese (0)
Maltese (1)
True label
Predicted label
51
2
8
108
100
80
60
40
20

Figure S16. Receiver Operating Characteristic (ROC) curves for classification of Maltese and non-Maltese pork using ANN models with different preprocessing methods: 1st Derivative, 2nd Derivative, Deresolve, Detrend, Median Filter, Multiplicative Scatter Correction (MSC), Orthogonal Signal Correction (OSC), Quantile Normalization, Raw, Standard Normal Variate (SNV), and SNV+Detrend. Left panels represent excluded rows (external validation), while right panels represent training datasets. The area under the curve (AUC) is shown for each model, with values close to 1.0 indicating strong classification performance.

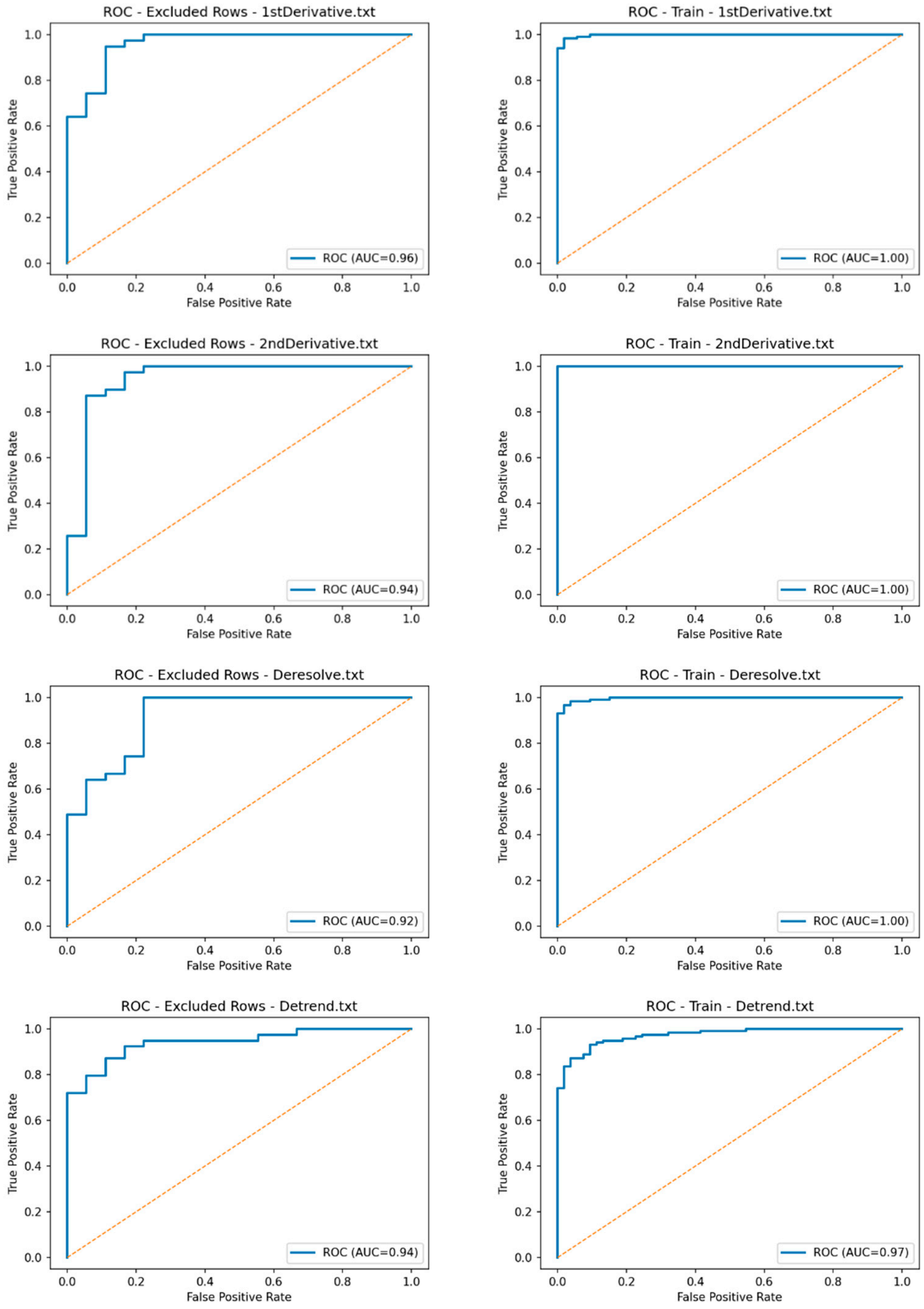

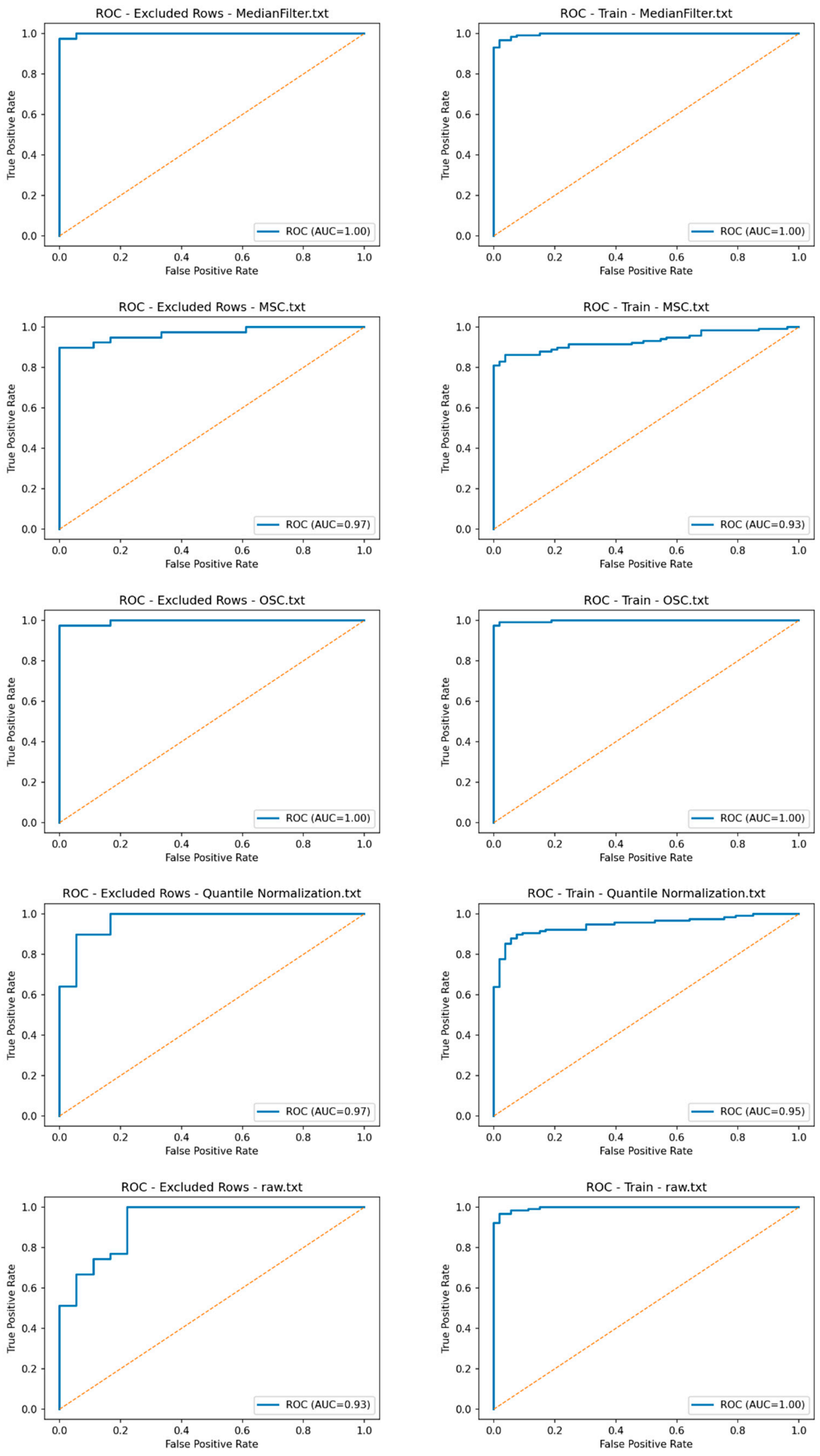
ROC - Excluded Rows - MedianFilter.txt
ROC (AUC=1.00)
ROC - Train - MedianFilter.txt
ROC (AUC=1.00)
ROC - Excluded Rows - MSC.txt
ROC (AUC=0.97)
ROC - Train - MSC.txt
ROC (AUC=0.93)
ROC - Excluded Rows - OSC.txt
ROC (AUC=1.00)
ROC - Train - OSC.txt
ROC (AUC=1.00)
ROC - Excluded Rows - Quantile Normalization.txt
ROC (AUC=0.97)
ROC - Train - Quantile Normalization.txt
ROC (AUC=0.95)
ROC - Excluded Rows - raw.txt
ROC (AUC=0.93)
ROC - Train - raw.txt
ROC (AUC=1.00)
True Positive Rate
False Positive Rate
0.0
0.2
0.4
0.6
0.8
1.0

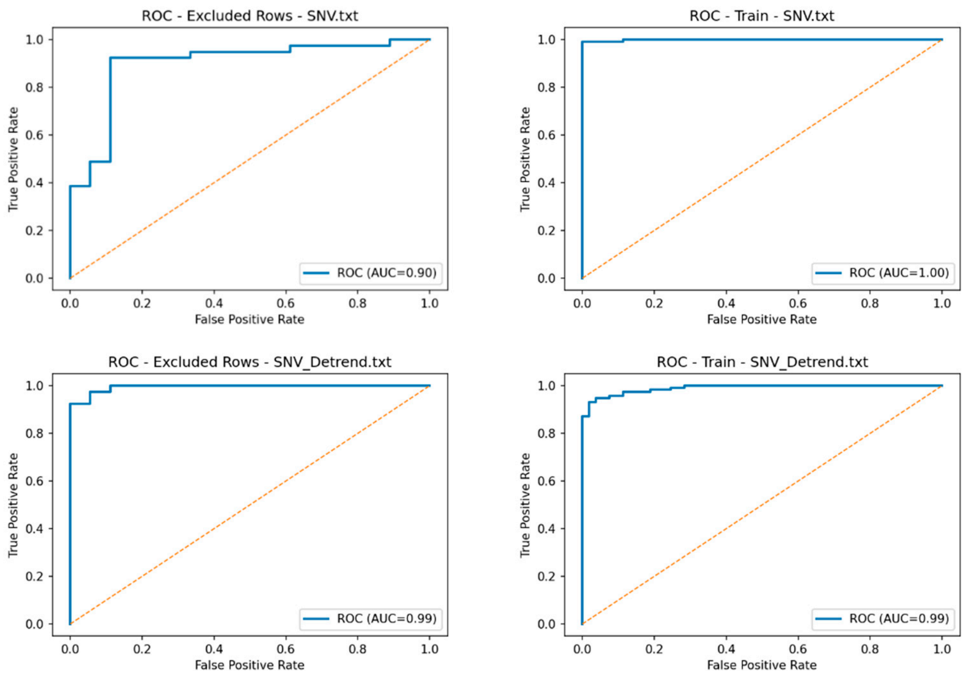
ROC - Excluded Rows - SNV.txt
ROC (AUC=0.90)
ROC - Train - SNV.txt
ROC (AUC=1.00)
ROC - Excluded Rows - SNV_Detrend.txt
ROC (AUC=0.99)
ROC - Train - SNV_Detrend.txt
ROC (AUC=0.99)
True Positive Rate
False Positive Rate
0.0
0.2
0.4
0.6
0.8
1.0

Figure S17. Region of Importance (ROI) plots showing the most influential wavenumber regions for discrimination between Maltese and non-Maltese pork. The black dashed line represents the normalized ATR-FTIR spectrum, while the red trace indicates the ROI importance values identified by chemometric modelling with different preprocessing methods: 1st Derivative, 2nd Derivative, Deresolve, Detrend, Median Filter, Multiplicative Scatter Correction (MSC), Orthogonal Signal Correction (OSC), Quantile Normalization, Raw, Standard Normal Variate (SNV), and SNV+Detrend. Shaded areas highlight spectral intervals consistently selected as informative across preprocessing strategies.

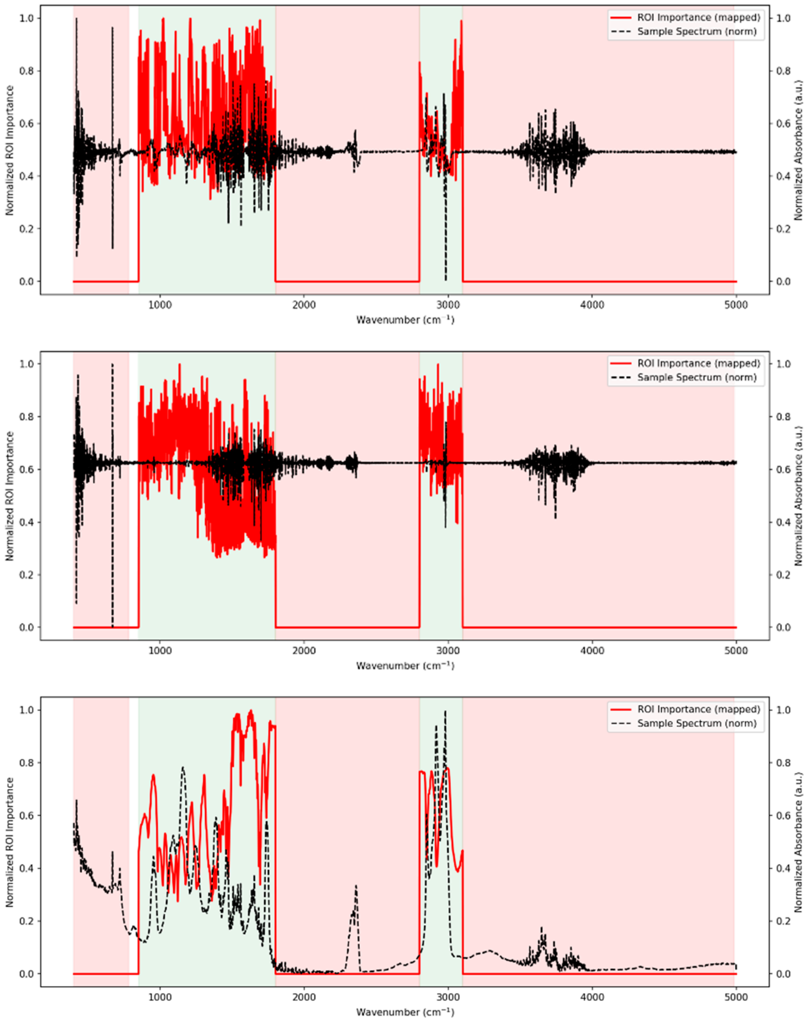

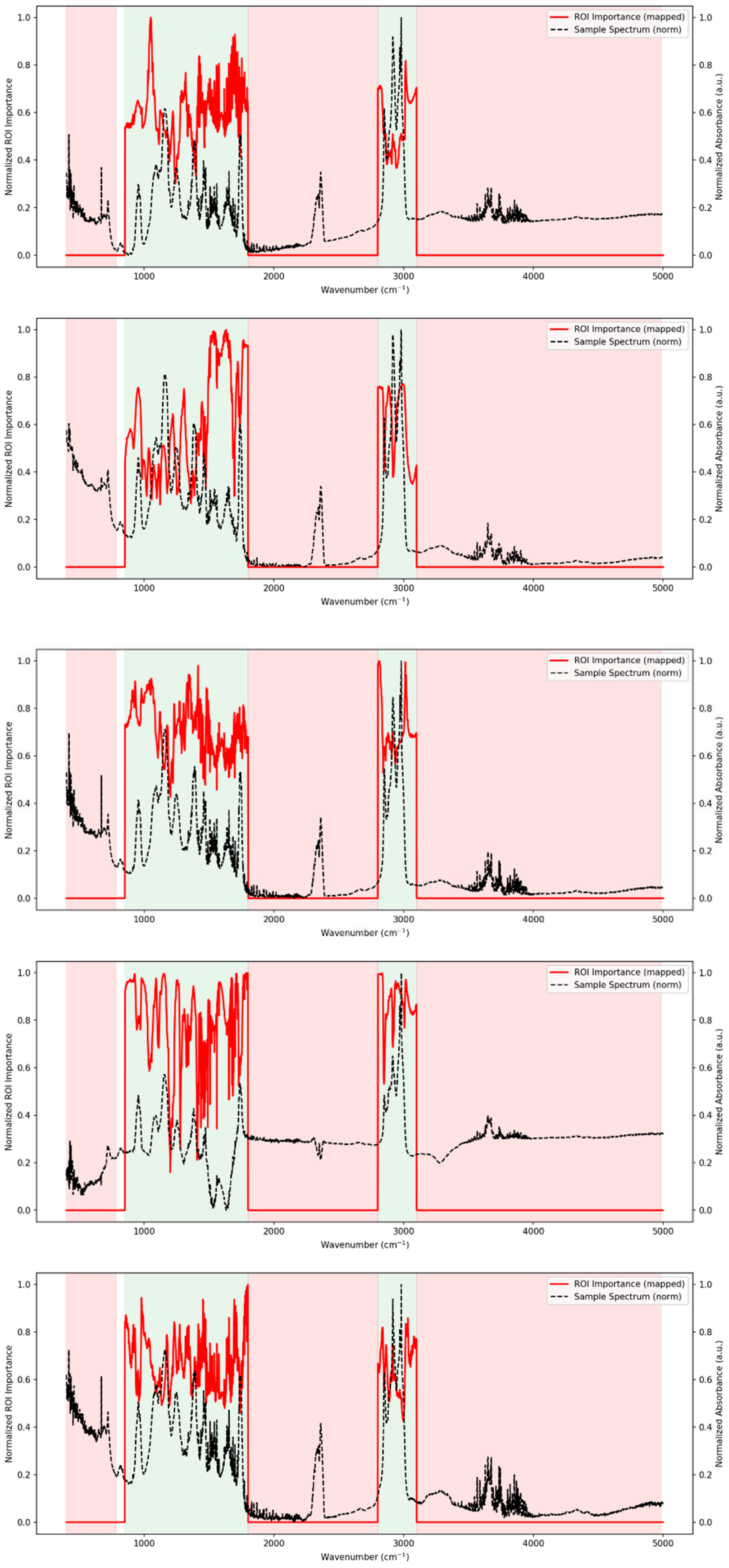
ROI Importance (mapped)
Sample Spectrum (norm)
Normalized ROI Importance
Normalized Absorbance (a.u.)
Wavenumber (cm^{-1})
1000
2000
3000
4000
5000
0.0
0.2
0.4
0.6
0.8
1.0

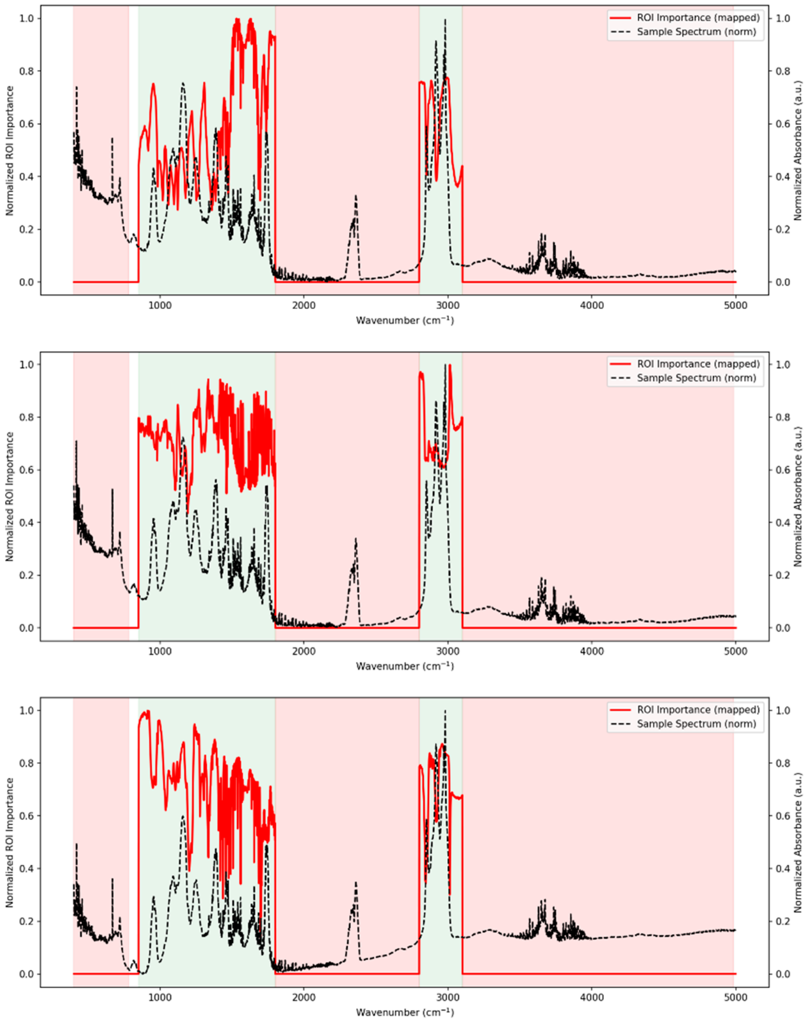

ROI Importance (mapped)
Sample Spectrum (norm)
Normalized ROI Importance
Normalized Absorbance (a.u.)
Wavenumber (cm^{-1})
ROI Importance (mapped)
Sample Spectrum (norm)
Normalized ROI Importance
Normalized Absorbance (a.u.)
Wavenumber (cm^{-1})
ROI Importance (mapped)
Sample Spectrum (norm)
Normalized ROI Importance
Normalized Absorbance (a.u.)
Wavenumber (cm^{-1})
